# TRIB3 promotes malignancy of head and neck squamous cell carcinoma via inhibiting ferroptosis

**DOI:** 10.1038/s41419-024-06472-5

**Published:** 2024-03-01

**Authors:** Li Chen, Wanzun Lin, Haojiong Zhang, Shikai Geng, Ziyu Le, Fangzhu Wan, Qingting Huang, Huaiyuan Chen, Xingyu Liu, Jiade J. Lu, Lin Kong

**Affiliations:** 1 Department of Radiation Oncology, Shanghai Proton and Heavy Ion Center, Fudan University Cancer Hospital, Shanghai, 201321 China; 2 Shanghai Key Laboratory of Radiation Oncology (20dz2261000), Shanghai, 201321 China; 3 Shanghai Engineering Research Center of Proton and Heavy Ion Radiation Therapy, Shanghai, 201321 China

**Keywords:** Tumour biomarkers, Cancer prevention

## Abstract

Tribbles pseudokinase 3 (TRIB3) has been identified recently as a novel oncogene in several cancers. Still, further extensive research is imperative to elucidate its function and the molecular mechanisms underlying its involvement in the progression of head and neck squamous cell carcinoma (HNSCC). In our study, we found that TRIB3 silencing significantly promoted cell death by inducing ferroptosis. The interaction of TRIB3 with Transcription Factor 4 (TCF4) and β-catenin created a heterotrimeric complex, which directly interacts with the ALOXE3 promoter, detrimentally impacting its activation. The consequential partial neutralization of ferroptosis induced by TRIB3 deficiency is observed through the implementation of ALOXE3 knockdown. Furthermore, the study demonstrated that the molecular inhibitor hesperidin, targeting TRIB3, not only reduced cell malignancy but also induced ferroptosis, thereby suppressing tumor growth. Overall, our findings unequivocally validate the proposition that TRIB3 deficiency precipitates the iron death mechanism, thereby indicating that the strategic targeting of TRIB3 could emerge as an innovative therapeutic strategy for HNSCC.

## Introduction

Head and neck squamous cell carcinoma (HNSCC) stands as the sixth most malignancy worldwide, with approximately 890,000 new cases and 450,000 reported fatalities annually [1]. Despite the availability of diverse treatment modalities for HNSCC, significant progress in survival rates has been realized over the last three decades. Consequently, the imperative lies in the discovery of novel treatment modalities and the development of effective strategies for addressing HNSCC. Notably, ferroptosis has recently emerged as a distinctive form of cell death [2]. It unveils a promising avenue for anticancer therapy. Furthermore, contemporary research underscores the pivotal role of ferroptosis in repressing the malignant phenotype of HNSCC and retarding disease progression [3]. Thus, an in-depth exploration of the underlying mechanisms in HNSCC becomes instrumental in harnessing ferroptosis as a potential therapeutic approach for the condition.

Within the realm of Drosophila tribbles gene homologs, the mammalian domain boasts three counterparts, among which is the noteworthy Tribbles pseudokinase 3 (TRIB3) [4, 5]. Remarkably, TRIB3 has gained prominence for its involvement in restraining mitotic processes within embryonic and germ cells. As a stress sensor, TRIB3 stands responsive to alterations in the cellular microenvironment and various stress-inducing factors, including insulin, glucose deficiency, and the accumulation of misfolded proteins within the endoplasmic reticulum [6]. Furthermore, TRIB3’s engagement extends to dire maladies and chronic inflammation, as it interfaces with a spectrum of intracellular signaling pathways. These encompass ubiquitin ligase, autophagy, proteasomal degradation systems, transcription factors, and constituents of pivotal signaling networks, such as JAG1/Notch, TGF-β, and MAPK-ERK [7]. With mounting recognition, TRIB3 has assumed a role as a promoter of tumorigenesis across diverse cancer types [8–12]. Nevertheless, its specific contribution to HNSCC oncogenesis remains predominantly uncharted territory.

Our investigation has unveiled the pivotal role of TRIB3 in driving the initiation and progression of HNSCC by the inhibition of ferroptosis. TRIB3 plays a central role within a crucial heterotrimeric complex, as it interacts with β-catenin-TCF4, facilitating direct binding to the arachidonate lipoxygenase 3 (ALOXE3) promoter region. This interaction, in turn, results in the suppression of ALOXE3 expression and the subsequent hindrance of ferroptosis. Acknowledging the significance of TRIB3 in the context of ferroptosis, we have developed a highly selective and potent TRIB3 inhibitor, hesperidin. Hesperidin exhibits robust antitumor properties in HNSCC. These findings illuminate a promising therapeutic approach focused on targeting TRIB3 to mitigate the progression of HNSCC.

## Materials and methods

### Data acquisition and processing

Clinical Proteomic Tumor Analysis Consortium (CPTAC, https://proteomics.cancer.gov/programs/cptac, *n* = 162), Cancer Genome Atlas (TCGA, http://cancergenome.nih.gov; *n* = 501) and Gene Expression Omnibus (GEO, https://www.ncbi.nlm.nih.gov/geo/, accession number: GSE41613; *n* = 97) aided in the retrieval of 760 samples with relevant clinicopathological data and RNA-seq profiles.

### Cell lines and transfection

HNSCC cell lines (Tca8113, Cal27, Tu686, SCC9, and FaDu) and normal oral keratinocytes (NOK) cells were purchased form American Type Culture Collection (ATCC, USA), and tested for negative mycoplasma contamination using Mycoplasma Detection Kit (vazyme, China). Cal27, SCC9 and FaDu cell lines were authenticated by STR analysis (Suzhou, China). Tca8113, Cal27, Tu686, and SCC9 cell cultures were performed in Dulbecco’s Modified Eagle Medium (DMEM). FaDu cell culture was conducted in Minimum Essential Medium (MEM). Both mediums were supplemented with 10,000 U/mL of penicillin-streptomycin and 10% fetal bovine serum. NOK cell culturing was conducted in a keratinocyte serum-free medium (KSFM) enriched with rEGF. HNSCC cells were transfected with TRIB3/shTRIB3 lentiviral vectors (Genechem, Shanghai, China) and TCF4/shTCF4/shALOXE3 lentivirus plasmid vector (Genechem, China) with the aid of Lipofectamine®3000 (Invitrogen, Carlsbad, CA, USA) for subsequent analyses for 48 h. These cells subjected to culture in an incubator maintained at 37 °C and 5% CO_2_.

### Colony formation assay

First, 6-well plates were used to seed 1000 cells in order to perform colony formation assay. Colonies were colored with crystal violet staining solution (Beyotime, Shanghai, China) and fixed with 4% paraformaldehyde after 2 weeks. A colony counting machine (GelCount, Oxford, UK) was utilized to determine colony counts and capture the images.

### Cell viability assay

Cell counting Kit-8 (CCK8 kit, Doijindo, Kyushu, Japan) aided in measuring cell viability. In addition, 96-well plates were employed to seed cells. Media containing 10% CCK8 reagent replaced the cell culture media after undergoing proper treatment. The plate was incubated for 1 h at 37 °C. Finally, cell viability was quantified by measuring the OD value at 450 nm using a microplate reader (FLUOstar Omega, Offenburg, Germany).

### EdU incorporation assay

Execution of an EdU incorporation experiment was conducted with the aid of the BeyoClick EdU Cell Proliferation Kit (Beyotime, China). It was performed as recommended by the manufacturer. Finally, a confocal laser scanning microscope (Carl Zeiss, Oberkochene, Germany) was utilized to perform fluorescence imaging.

### Cell death assay

Cell collection was followed by resuspension in 100 μL of binding buffer, and subsequently, 5 μL of propidium iodide (PI) (BD Biosciences, Franklin Lakes, NJ,USA) was added. Incubation was carried out at room temperature in a light-protected environment for a duration of 5 min, and then analyze using a flow cytometer (Beckman Coulter, California, USA).

### Soft agar colony formation assay

Prepare the foundational layer by amalgamating 750 μL of 1.2% agarose with DMEM medium containing 20% FBS, and gently dispense this mixture into a 6-well culture plate. Allow the agarose to solidify into a stable base. Next, craft the upper layer by blending 200 μL of a 0.7% agarose solution with an additional 200 μL of medium containing 5000 cells; introduce this compound into the 6-well plate once the foundational layer has congealed. Following this, supplement with 2 mL of medium and incubate for 14 days. Afterward, apply crystal violet staining and capture images.

### Transwell migration and invasion assays

Migration and invasion assays were executed using Transwell chambers (Millipore, Billerica, USA). In the migration assay, 2.5 × 10^4^ cells were seeded into the upper chamber in serum-free culture medium, while the lower chamber was filled with DMEM medium containing 10% FBS. For the invasion assay, the chamber was pre-coated with Matrigel (BD Biosciences, USA), with subsequent procedures mirroring those of the migration assay. Following 48 h of cellular migration or invasion, the cells in the upper chamber were gently removed using a cotton swab, fixed, and then stained with crystal violet. The quantification of migrated or invaded cells was performed by counting them under an inverted optical microscope.

### Apoptosis assay

FITC Annexin V Apoptosis Detection Kit I (BD Pharmingen, USA) aided in identifying apoptotic cells. Cell collection was followed by resuspension in 100 μL of binding buffer, and added with 5 μL of PI and FITC Annexin V. Incubation at room temperature in the absence of light was conducted for 15 min. A flow cytometer calculated the percentage of apoptotic cells.

### Real-time quantitative PCR (qPCR)

First, total RNA was extracted from cells by employing an RNeasy kit (Qiagen, Dusseldorf, Germany). Later on, the PrimeScript® RT reagent Kit (Takara, Dalian, China) was employed to perform the reverse transcription. The resulting complementary DNA template was mixed with qPCR primers. Thus, quantitative real-time qPCR was conducted on the Applied Biosystems QuantStudio 5 Real-Time qPCR System (Applied Biosystems, California, USA) by TaKaRa SYBR® Premix Ex Taq™. For quantification of gene expression, the 2^−ΔΔCt^ method was used with expression normalized to β-actin.

### Western blotting and immunofluorescence

For western blotting, cells were treated with phenylmethylsulfonyl fluoride (PMSF) (Beyotime, China) and RIPA buffer for total protein. The Nuclear and Cytoplasmic Protein Extraction Kit (Beyotime, China) was utilized to separate cells for protein extraction following the guidelines of the manufacturer. The BCA Assay Kit (Beyotime, China) was utilized to measure the concentration of proteins. Sodium dodecyl-sulfate polyacrylamide gel electrophoresis (SDS-PAGE) separated the 20 μg of protein sample, which was then blotted on a polyvinylidene fluoride (PVDF) membrane. The membrane was then blocked at room temperature for 1 h. Incubation at 4 °C with primary antibodies overnight and incubation for 1 h with the HRP-conjugated antibodies at room temperature. Bio-Rad system (Hercules, California, USA) was utilized to visualize the bands according to the provided protocols. Following are the antibodies utilized for Western blotting and their concentrations: TRIB3 (Abcam, Cambridge, UK, ab75846, 1:10000), β-catenin (Cell Signaling Technology, Boston, USA, 8480, 1:1,000), TCF4 (Cell Signaling Technology, 2569, 1:1,000), ALOXE3 (ABclonal, Wuhan, China, A8245,1:2,000), Histone-H3(Cell Signaling Technology, 60932, 1:1,000), β-Tubulin (Cell Signaling Technology, 2146, 1:1,000), β-actin (Cell Signaling Technology, 93473, 1:1,000), Anti-rat IgG (Cell Signaling Technology, 98164, 1:1,000).

For immunofluorescence experiments, cells were seeded in confocal dishes. They were initially fixed with 4% paraformaldehyde for 15 min, followed by permeabilization with 0.5% Triton X-100 for 10 min and subsequent blocking for 1 h. Primary antibodies were then added and incubated overnight at 4 °C. After PBS washing, cells were incubated with goat anti-mouse and goat anti-rat secondary antibodies at room temperature for 1 h, followed by DAPI staining for nuclear visualization. Images were observed under a Zeiss LSM510 confocal microscope. Following are the antibodies utilized for immunofluorescence and their concentrations: TRIB3 (Proteintech, China, 13300-1-AP, 1:100), β-catenin (Cell Signaling Technology, 2677 S, 1:200), TCF4 (NOVUS, H00006925-M03, 10 μg/ml), Anti-rat IgG (abcam, ab15007, 1:1,000), Anti-mouse IgG (abcam, ab150115, 1:1,000).

### Lipid peroxidation assay

A day before treatment, the cells were seeded in 6-well plates in triplicate. They were pretreated with or without drugs. After 48 h, the cell culture medium in each well was replaced with fresh medium containing 5 μM BODIPY 581/591 C11 dye (Invitrogen, USA), and the incubation was carried out at 37 °C for 30 min. Afterward, cell suspensions were obtained, and the levels of lipid peroxidation were assessed using a flow cytometer.

### Malondialdehyde (MDA) assay

Cells were seeded in 6-well plates in triplicate the day before treatment. They were pretreated with or without drugs. After 48 h, lipid Peroxidation MDA Assay Kit (Beyotime, China) aided in determining relative MDA levels in cells. Lastly, to calculate MDA level using a microplate reader at the absorbance in 532 nm.

### Detection of intracellular Fe

A day prior to treatment, 6-well plates were used to seed cells in triplicate. They were pretreated with or without drugs. After 48 h, the cell culture medium in each well was replaced with fresh medium containing 1 µmol/L FerroOrange solution (Dojindo, China), and the incubation was carried out at 37 °C for 30 min. Afterward, cell suspensions were obtained, and the levels of Fe^2+^ were assessed using a flow cytometer.

### Transmission electron microscopy (TEM)

Firstly, Cal27 cells were fixed with 2.5% glutaraldehyde and 0.1 M cacodylate buffer for 2 h. Then they were trypsinized and cleaned with cold PBS. Next, cells were exposed to 0.1 M cacodylate and 1% osmium tetroxide. Different concentrations of ethanol aided in dehydrating the fixed cells. Cells were then embedded in epoxy resin. Hitachi TEM system was employed to observe cell ultrastructures and obtain images at 80 kV.

### Histology and immunohistochemistry (IHC) analysis

Collection and fixation of tumor samples were conducted for a night in 10% neutral-buffered formalin. Tumors were rinsed with PBS. Then they were transferred to 70% ethanol for embedding, sectioning, and eosin and hematoxylin staining. The sections were hydrated, deparaffinized, and boiled in sodium citrate buffer for antigen retrieval to perform IHC staining. Incubation was then conducted with anti-goat secondary and primary antibodies. At first, they were colored with diaminobenzidine and then again stained in hematoxylin. Microscope aided in the capture of images.

### Wound healing

Briefly, cells were plated in 6-well plates to form a full monolayer. A wound was formed by scratching with a 200 μL pipette tip. The intercellular distance was calculated at 0 h and 48 h. Following is the formula to measure the wound healing ratio: migration distance / primary intercellular distance × 100%.

### RNA-sequencing (RNA-seq) experiment and analysis

HNSCC cells aided in extracting total RNA. Following the standard Illumina RNA-seq instructions, the cDNA library was prepared. A differential expression study of RNAs among two different groups was conducted by the DESeq2 program. The genes/transcripts with absolute fold change ≥2 and FDR < 0.05 were considered to be expressed differentially. The studies of Gene Ontology (GO) and the Kyoto Encyclopedia of Genes and Genomes (KEGG) aided in assessing functional enrichment for defining the biological activities of differential expression genes (DEGs). Comparison between gene set enrichment in low and high-risk groups of MSigDB Collection (c5.go.v2023.1.Hs.symbols.gmt and c2.cp.kegg.v7.4.symbols.gmt) was conducted by Gene Set Enrichment Analysis (GSEA). With the threshold of FDR < 0.05, the estimated *t*-test was subjected to FDR correction.

### Co-immunoprecipitation (Co-IP)

After incubating the cell lysate with Protein A/G Plus-Agarose and the specified antibody overnight at 4 °C, the precipitate was washed 5 times with PBS. Then boiled for 5 min in dodecyl-sulfate sample buffer. Immunoblot with specific antibodies and SDS-PAGE aided in resolving the co-precipitates.

### Glutathione S-transferase pull-down (GST Pull-down)

The purified TCF4 protein or β-catenin were co-incubated overnight at 4 °C with GST or GST-TRIB3 fusion proteins, in the presence of glutathione-sepharose beads. The expression of GST fusion proteins was subsequently confirmed through SDS-PAGE and Coomassie Blue staining.

### Chromatin immunoprecipitation (ChIP)

SimpleChIP Enzymatic Chromatin IP Kit (Cell Signaling Technology, MA) was used in executing the chromatin immunoprecipitation analysis as recommended by the manufacturer. The quantification of the resulting precipitated DNA samples was done by qPCR.

### Dual-luciferase reporter (DLR) assays

24-well plates were employed for seeding Cal27 cells at 1 × 10^5^ cells/well. ALOXE3 promoter reporter plasmid and pProUTR-Reporter-Ctrl plasmid were introduced together into each well using Lipofectamine 2000, either with or without the additional TCF4 overexpression plasmid. After 48 h, luciferase activities were assessed using a dual-luciferase reporter assay kit (Promega, Wisconsin, USA) as per the manufacturer’s instructions. The measurements were taken with the aid of a Hybrid Multi-Mode Microplate Reader SYNERGY H1 (BioTek, Vermont, USA). For every well, the firefly luciferase activity was normalized to Renilla luciferase.

### Animal studies

Specific pathogen-free BALB/c female nude mice (6–8 weeks old) were acquired from SPF (Beijing) Biotechnology Co., Ltd. Utilization of the mice was done following the guidelines of Shanghai Proton and Heavy Ion Center Institutional Animal Care and Use Committee. The ethics committee allowed a maximum tumor size of 2000 mm^3^.

In order to assess the TRIB3 effect on tumor growth, every mouse was inoculated beneath the skin into the right flank with 5 × 10^6^ TRIB3 knockdown or control FaDu cells (*n* = 5). Evaluation of the tumor volume was conducted using the formula: volume = 0.5 × length × width^2^. The mice were administered Liproxstatin-1 intraperitoneally once a day for a duration of 5 days (50 mg/kg; Selleck, Texas, USA). To assess the impact of hesperidin on tumor growth, each mouse was inoculated with 5 × 10^6^ FaDu cells subcutaneously into the right flank. Upon reaching a tumor volume of 50–100 mm^3^, the mice were randomly placed into four groups (*n* = 6). They were treated daily with hesperidin (80 mg/kg or 40 mg/kg) or PBS respectively. After the treatment period, tumors were removed after killing the animals. Finally, tumors were weighed for IHC staining.

### Molecular docking

During the docking analysis process, the *Prep Wiz* module in the Schrödinger software package was used to prepare the protein for pre-treatment operations. The United States Food and Drug Administration (FDA) small molecule library was used in this virtual screening. All compounds were preliminarily screened by *Lipinski’s rule of five and the Veber rule* module in DS4.0 to eliminate molecules with poor drug-likeness properties. The remaining molecules were prepared using the *LigPrep* module in the Schrödinger software package. The force field was prepared using OPLS_ 2005. The protonation of molecules was operated using the *Epik* module at pH 7.4. The active site of the protein was found by using the *SiteMap* module in the Schrödinger software package. The Canvas 1.1 program was used for filtration to identify whether the screened compounds had a pan-assay interference compounds (PAINS), structure. To predict the pharmacokinetic properties of the screened compounds. The QikProp 3.2 program in the Schrödinger software package was used. It calculated the corresponding properties to preliminarily evaluate the ADME characteristics of the compound. Selection of the optimal docking condition Glide force field was conducted, and docking research was performed using TRIB3 protein files as receptors. The Glide algorithm is used to dock with HTVS, SP, and XP docking accuracy. The Screening was conducted through the FDA small molecule library, and the final screened small molecules were docked.

### Chemicals

All the inhibitors and inducers were purchased from Selleck Chemicals (USA). The Ferrostatin-1 (Ferr-1) with 5 μM, DFO with 50 μM, 3-Methyladenine (3-MA) with 5 mM, Bafilomycin A1 (BafA1) with 50 nM, Z-VAD-FMK (Z-VAD) with 10 μM, Necrostatin-1 (Nec-1) with 2 μM, Erastin with 10 μM stimulated the indicated cells for 48 h before detection, respectively.

### Statistical analysis

SPSS 20.0 and GraphPad Prism 9.0 were utilized to analyze statistical data. To compare between two groups, a two-tailed unpaired Student *t*-test was employed. One-way ANOVA was applied in case three or more groups were compared. In order to evaluate the *P* values for Kaplan–Meier survival curves, the two-tailed log-rank test was utilized. Data was reported as the mean ± standard deviation (SD). The corresponding *P* values were indicated in the figures. All the experiments were repeated at least thrice. Statistically significant differences were defined at *P* < 0.05.

## Results

### Overexpressed TRIB3 in HNSCC and poor prognosis

**Fig. 1:**
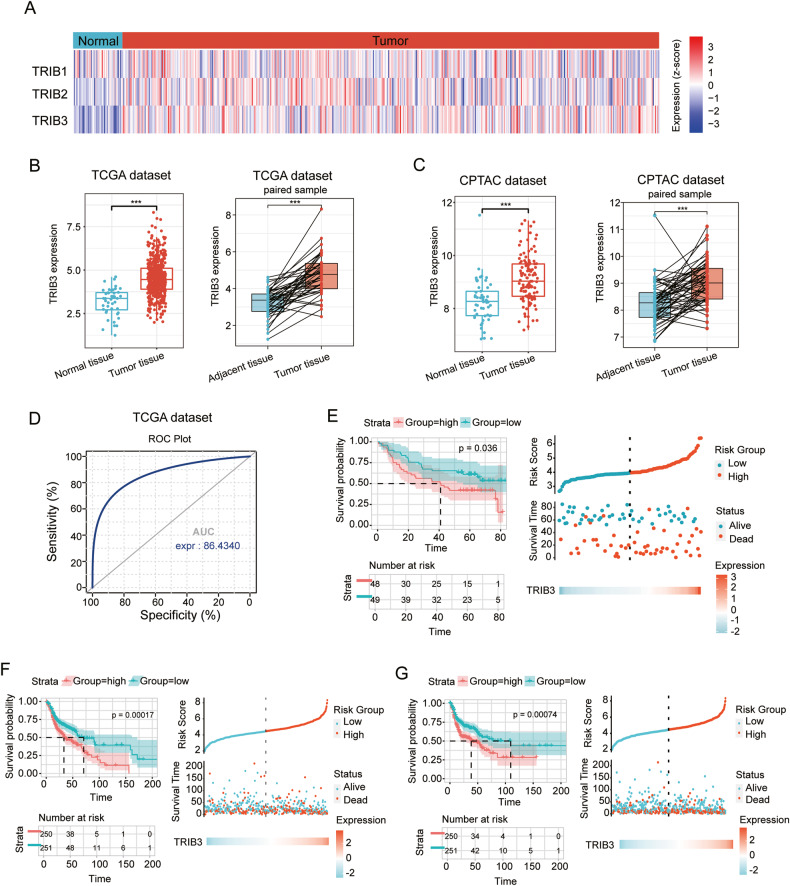
Associations between prognostic significance and TRIB3 expression in HNSCC. **A** A Heat Map of TRIB1, TRIB2, and TRIB3 gene expression in HNSCC and normal tissues. **B**, **C** Comparison analysis of TRIB3 levels among HNSCC tissues and normal tissues or adjacent tissues in the TCGA database or the CPTAC database. **D** ROC curve indicating the predictive value of TRIB3. **E** Comparison of the low and high expression of TRIB3 via Kaplan–Meier OS curve and survival status in GEO database or **F** TCGA database. **G** Comparison between the low and high expression of TRIB3 via Kaplan–Meier PFS curve and survival status in TCGA database. ***P < 0.001, **P < 0.01, *P < 0.05.

Analysis of data from TCGA dataset revealed significant overexpression of members of the TRIB pseudokinases protein family, including TRIB1, TRIB2, and notably, TRIB3, in HNSCC tissues in comparison to normal tissues (Fig. 1A, B). This dysregulation of TRIB3 in HNSCC was further confirmed using the CPTAC cohort (Fig. 1C). Subsequently, we generated a receiver operating characteristic (ROC) curve to assess the diagnostic significance of TRIB3 in HNSCC. The area under the ROC curve, which reached 86.43%, underscores the high predictive value of TRIB3 (Fig. 1D). Additionally, we investigated the predictive capacity of TRIB3 in both the GEO database and TCGA.

An analysis employing the Kaplan–Meier method unveiled a robust association between heightened TRIB3 levels and diminished progression-free survival (PFS) and overall survival (OS) in individuals afflicted with HNSCC. Notably, the group with high TRIB3 expression exhibited a significantly higher mortality rate compared to the low-expression group (Fig. 1E–G). In summation, these findings underscored the potential role of TRIB3 as a pivotal regulator in the carcinogenesis of HNSCC.

### TRIB3 silencing inhibits the malignancy of HNSCC

**Fig. 2:**
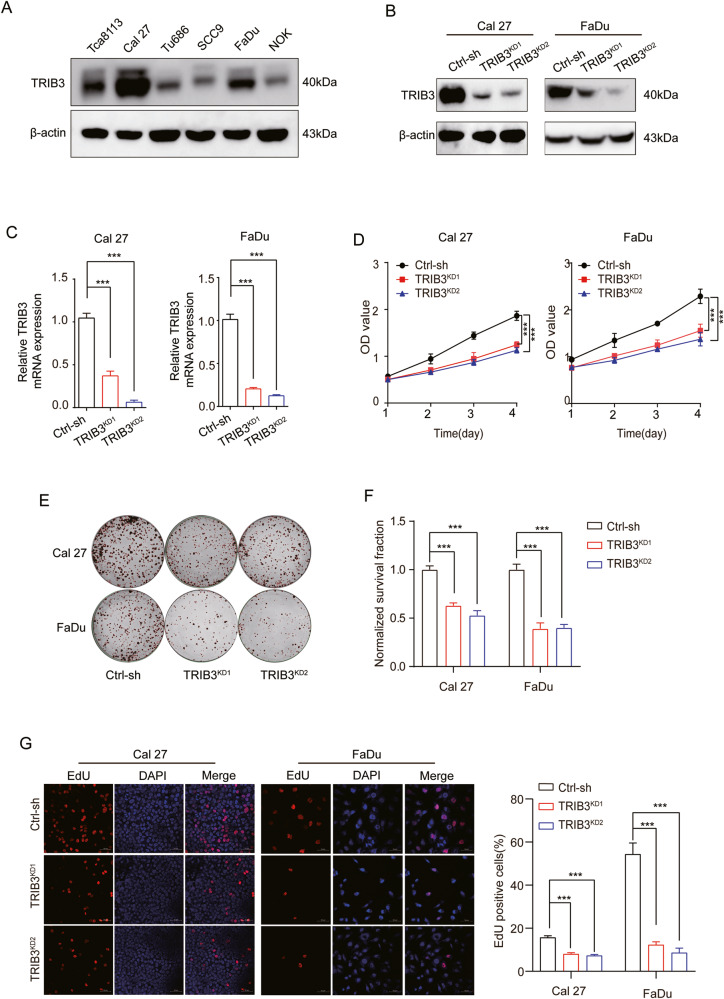
TRIB3 silencing inhibits HNSCC cell viability and proliferation. **A** Western blot to detect protein levels of TRIB3 in normal NOK cell and HNSCC cell lines. Cal27 and FaDu cells were transfected with two independent TRIB3 shRNAs. Western blot (**B**) and qPCR (**C**) to detect TRIB3 levels in TRIB3-knockdown and control cells. **D** OD450 readings were plotted over time using the CCK8 assay. **E**, **F** Findings of a clonogenic analysis. Red circles demonstrated the counted clones. **G** Visualizztion of DNA replication using EdU. Red-stained cell nuclei demonstrated DNA replication. Scale bar: 50 μm. ***P < 0.001, **P < 0.01, *P < 0.05.

To explore the potential involvement of TRIB3 in tumorigenesis and progression, we assessed the expression of TRIB3 in HNSCC cell lines in comparison to NOK cells. Our analysis confirmed elevated levels of TRIB3 protein in HNSCC cells, particularly in the FaDu and Cal27 cell lines (Fig. 2A). Subsequently, we achieved successful silencing of TRIB3 in Cal27 and FaDu cell lines, as well as overexpression of TRIB3 in the SCC9 cell line (Fig. 2B, C and Fig. S1a, b).

Consistent with the hypothesis of this study, the CCK-8 analysis revealed that TRIB3 silencing substantially inhibited the proliferation of FaDu and Cal27 cells (Fig. 2D). In contrast, TRIB3-overexpression increased the SCC9 cell proliferation (Fig. S1d). Consistently, similar observations were obtained by using EdU assays, clonogenic assays, soft agar clonal formation assays, and PI staining (Fig. 2E–G, Fig. S1c, e and Fig. S2a, b). Furthermore, we also found that knockdown TRIB3 can inhibit the migration and invasion of HNSCC cells (Fig. S2c, d). Taken together, our data collectively indicated that TRIB3 promoted cell malignancy in HNSCC, signified its potential as a promising therapeutic target.

### Promotion of ferroptosis by TRIB3 silencing in HNSCC

**Fig. 3:**
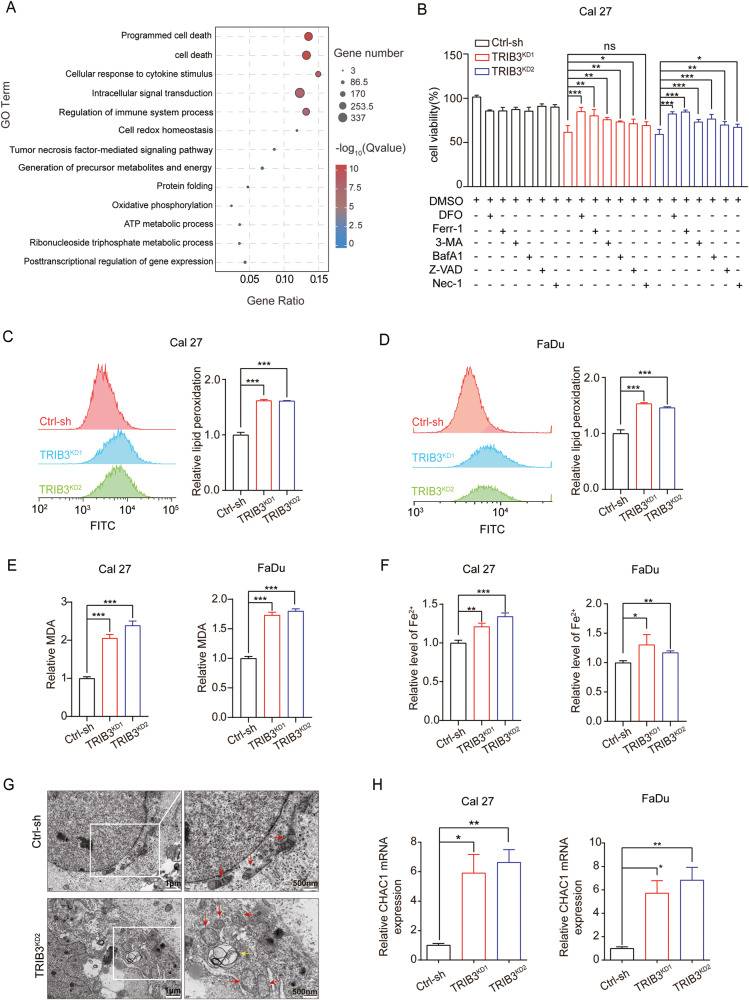
Promotion of cell death by TRIB3 silencing largely through inhibiting ferroptosis. **A** GO analysis of DEGs between Cal27 TRIB3-knockdown and control cells. **B** CCK-8 assay detected the cell viability of Cal27 TRIB3-knockdown and control cells, treated with or without cell death inhibitors. **C**, **D** Lipid peroxidation of TRIB3-knockdown and control cells. **E** MDA in TRIB3-knockdown and control cells. **F** The Fe^2+^ level in control cells and TRIB3-knockdown. **G** Transmission electron microscopy images of Cal27 TRIB3-knockdown and control cells. The red arrows demonstrate mitochondria. Yellow arrows demonstrate autophagosomes. Scale bars: right, 500 nm; left, 1 µm. **H** qPCR analysis of CHAC1 expression in control and TRIB3-knockdown cells. ***P < 0.001, **P < 0.01, *P < 0.05.

To clarify the molecular mechanism via which TRIB3 regulates cell growth in HNSCC, RNA-seq analysis was performed from control and TRIB3-knockdown cells (Fig. 3A). GO analysis revealed a significant enrichment of differentially expressed genes within the categories of ‘Programmed cell death’ and ‘Cell death.’ Further investigation was undertaken to elucidate their interrelation and identify which cell death models plays a dominant role in the regulation of cell death in TRIB3-knockdown cells. TRIB3-knockdown cells were exposed to multiple inhibitors of cell death. Compared with the cells treated with Z-VAD (an apoptosis inhibitor), Nec-1 (a necroptosis inhibitor), 3-MA and BafA1 (an autophagy inhibitor), the ferroptosis inhibitors (Ferr-1 and DFO) significantly rescued TRIB3-knockdown cells from death (Fig. 3B and Fig. S3a). Indeed, the GSEA revealed the enrichment of ferroptosis-related pathways within the TRIB3-knockdown group. These pathways encompass the epoxygenase P450 pathway and the negative regulation of lipid biosynthetic processes (Fig. S3b). Therefore, the data robustly suggested that ferroptosis denotes a dominant unit of the silencing TRIB3-induced cell death response in HNSCC cells.

Ferroptosis, a recently discovered mode of cell death, distinguishes itself from autophagy, necroptosis, and apoptosis. In our investigation, we explored ferroptosis-specific markers [13]. Initially, TRIB3 knockdown was found to be associated with elevated lipid peroxidation, Fe^2+^ and MDA generation in Cal27 and FaDu cells (Fig. 3C–F), while conversely, the overexpression of TRIB3 in SCC9 cells led to a decrease in these factors (Fig. S1g–i). Secondly, TEM analysis revealed that TRIB3 suppression resulted in the presence of shrunken mitochondria exhibiting heightened membrane density, a morphological hallmark linked to ferroptosis (Fig. 3G). Besides, ferroptosis marker genes CHAC1 was upregulated in TRIB3-knockdown cells (Fig. 3H), whereas their expression was attenuated in TRIB3-overexpressing cells (Fig. S1f).

**Fig. 4:**
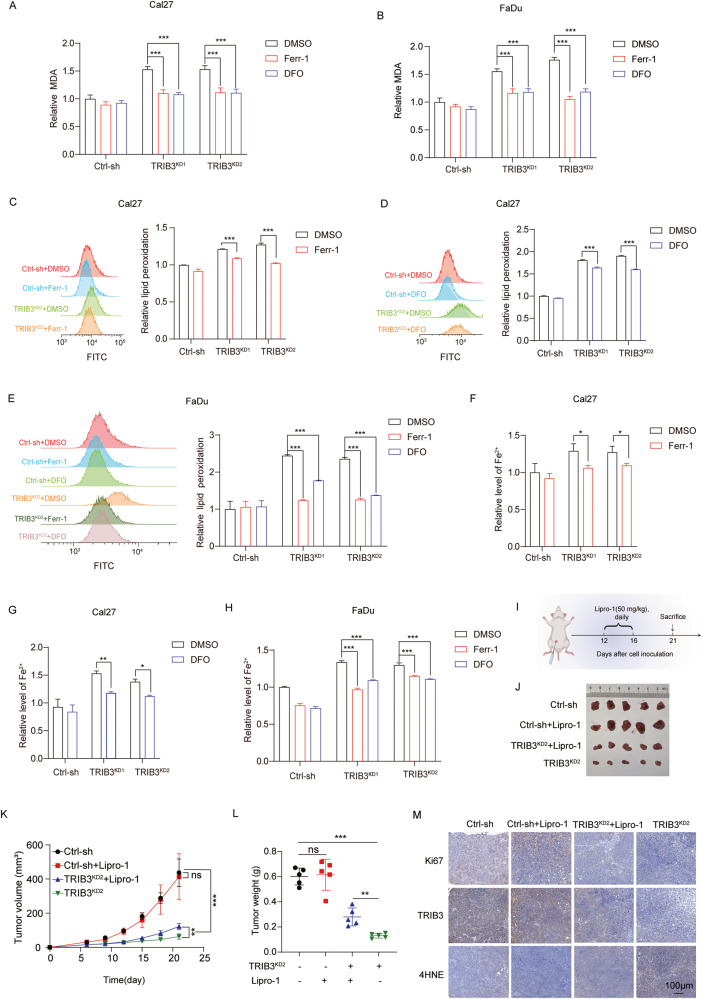
Induction of ferroptosis by TRIB3 silencing in HNSCC. **A**, **B** MDA in control and TRIB3-knockdown cells treated with Ferrostatin-1 (5 μm) or DFO (50 μm) for 48 h. **C**, **D**, **E** Lipid peroxidation in control and TRIB3-knockdown cells treated with Ferrostatin-1 (5 μm) or DFO (50 μm) for 48 h. **F**, **G**, **H** The level of Fe^2+^ in control and TRIB3-knockdown cells treated with Ferrostatin-1 (5 μm) or DFO (50 μm) for 48 h. **I** Schematic of TRIB3-knockdown subcutaneous tumors either treated with or without Liproxstatin-1. **J**, **K** Tumor growth and **L** weight in the xenograft model. **M** The expression of Ki67, TRIB3, 4-HNE using IHC assay. Scale bar: 100 μm. ***P < 0.001, **P < 0.01, *P < 0.05.

To further confirm TRIB3 silencing-induced cell death via ferroptosis in HNSCC cells, ferrostatin-1 and DFO were used to inhibit ferroptosis. The results revealed that both DFO and ferrostatin-1 had a significant mitigating effect on the increase in MDA levels, lipid peroxidation, and Fe^2+^ levels observed in TRIB3-knockdown cells (Fig. 4A–H). In addition, the TRIB3-overexpression cells were exposed to Erastin (a canonical ferroptosis inducer). It was observed that Erastin effectively reversed the low levels of lipid peroxidation, MDA, and Fe^2+^ in TRIB3-overexpressing cells (Fig. S1g–i).

The role of TRIB3 in the tumor-initiating capacity of HNSCC cells was examined, and the impact of TRIB3 silencing-induced ferroptosis was further validated in vivo. Subcutaneous injections of TRIB3-knockdown FaDu cells or control cells were administered to nude mice. After 12 days, the mice were subsequently subjected to treatment with liproxstatin-1 (Lipro-1), a ferroptosis antagonist, or left untreated (Fig. 4I). TRIB3 knockdown was observed to mitigate tumor growth in a subcutaneous xenograft model. Notably, this effect was significantly reversed with the administration of Lipro-1 (Fig. 4J–L). Consequently, the proportion of ki67-positive malignant cells in the TRIB3-knockdown group was notably lower in comparison to the control group, and this outcome was subsequently reversed upon treatment with Lipro-1 (Fig. 4M). 4-hydroxynonenal (4-HNE) represents a pivotal byproduct arising from lipid peroxidation [14], which engenders cellular dysfunction through the formation of adducts upon binding to proteins. The induced production of 4-HNE by TRIB3-knockdown was later reversed in the treatment of liproxstatin-1 (Fig. 4M). Taken together, these results demonstrated that TRIB3 inhibited ferroptosis in HNSCC cells. The process of ferroptosis can be intensified through the utilization of pharmacological compounds recognized as ferroptosis agonists.

### TRIB3 interaction with the TCF4 and β-catenin to form a heterotrimeric complex

**Fig. 5:**
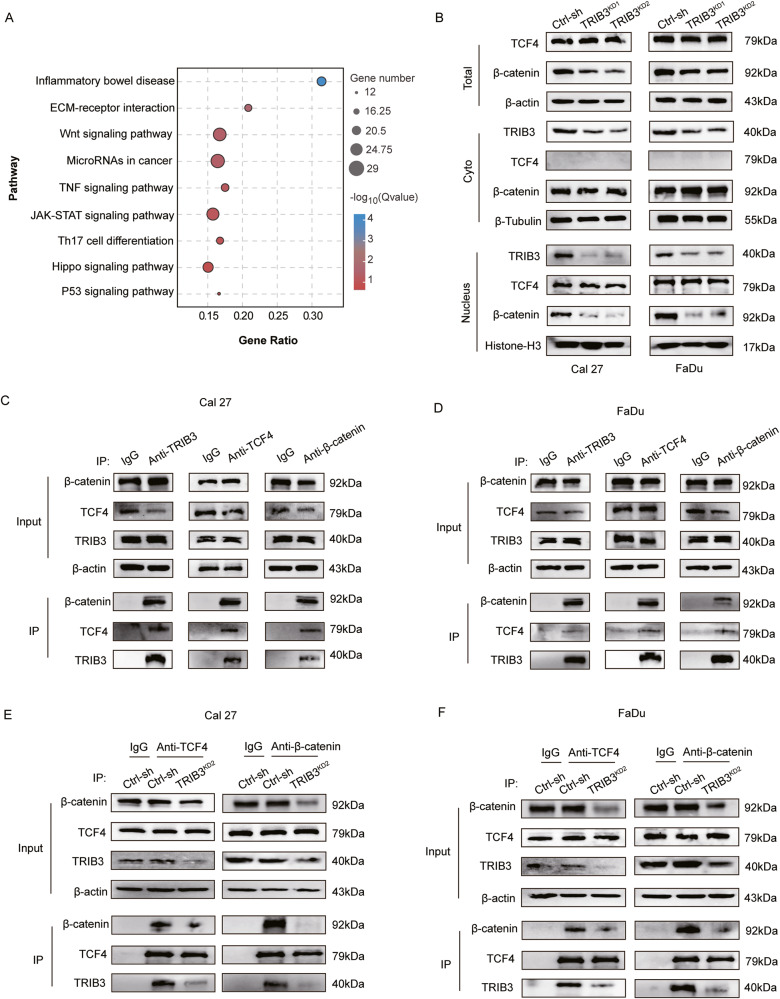
Interaction of TRIB3 with β-catenin and TCF4 to create a heterotrimeric complex. **A** KEGG analysis based on RNA-seq showed enrichment of DEGs in Wnt-signaling pathways. **B** Western blot to detect TCF4, β-catenin, and TRIB3 levels in the cytoplasm and nucleus of control and TRIB3-knockdown cells. **C**, **D** HNSCC cells extracts were immuno-precipitated and blotted with anti-TCF4, anti-TRIB3, or anti–β-catenin antibodies, respectively. Normal rabbit IgG was utilized as the control. **E**, **F** Co-IP of β-catenin and TCF4 in HNSCC cells with or without TRIB3 knockdown. ***P < 0.001, **P < 0.01, *P < 0.05.

The investigation into how TRIB3-knockdown induces ferroptosis was unknown. Initially, based on our RNA-seq results, the KEGG analysis indicated that differential expression genes (DEGs) were enriched in the Wnt-signaling pathway (Fig. 5A). Confirming the activation of the Wnt-signaling pathway was crucial in linking it to ferroptosis inhibition. The β-catenin-TCF4 complex can inhibit ferroptosis by promoting the transcription of glutathione peroxidase 4 (GPX4) [15]. Therefore, the possibility of TRIB3 influencing ferroptosis in HNSCC through the regulation of the β-catenin-TCF4 complex is worth exploring.

To delve deeper, we analyzed the levels of TCF4 and β-catenin in HNSCC cell lines. Interestingly, the TRIB3-silenced cell lines showed substantially lower levels of total and nuclear β-catenin, while the cytosolic β-catenin remained unchanged (Fig. 5B). Additionally, the level of TCF4 was unchanged in total and nuclear fractions, while its presence in the cytoplasm was almost negligible (Fig. 5B). Confocal analysis of HNSCC cells also showed that the nuclear expression of β-catenin decreased, while the cytosolic β-catenin remained unchanged in TRIB3 silencing cells compare to control cells, moreover, TCF4 mainly expressed in nuclear and its expression level remains almost unchanged in control or TRIB3 silencing cells (Fig. S4a). These results suggested that TRIB3 silencing effectively inhibited the expression of nuclear β-catenin but did not influence the level of TCF4.

The interaction between TRIB3, β-catenin, and TCF4 was further verified. The Co-IP assays revealed that TCF4, TRIB3, and β-catenin could be co-precipitated by every antibody in HNSCC cell lines (Fig. 5C, D). As the association between TCF4 and β-catenin is widely acknowledged, therefore, we performed GST Pull-Down to identify the interaction of the TRIB3 and β-catenin, TRIB3 and TCF4.The GST Pull-Down assay confirmed that the direct interaction between TRIB3, TCF4, and β-catenin (Fig. S4b). Interestingly, TRIB3-knockdown drastically decreased the β-catenin-TCF4 complex formation (Fig. 5E, F). Confocal analysis confirmed the co-localization of TRIB3 and TCF4 in the cell nucleus, while TRIB3 and β-catenin were found to co-localize in both the cell nucleus and cytoplasm in control group (Fig. S4a). These findings suggested that the formation of the β-catenin-TCF4 complex is reduced upon TRIB3-deletion. Collectively, these data strongly indicated that TRIB3-β-catenin-TCF4 can form a heterotrimeric complex.

### Negative modulation of ferroptosis via TRIB3‒β-catenin‒TCF4 heterotrimer by targeting ALOXE3

**Fig. 6:**
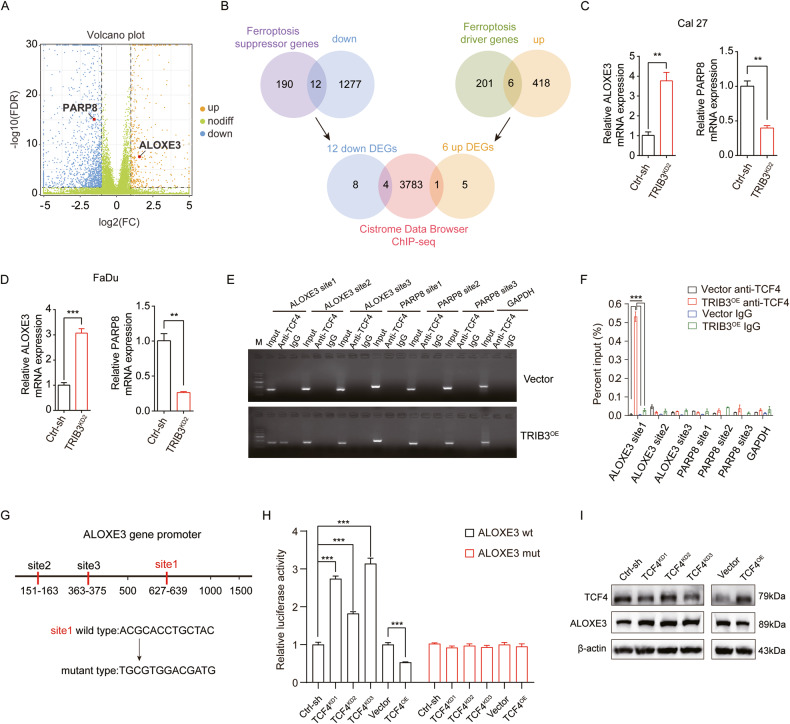
Inhibition of ALOXE3 activity by TRIB3‒β-catenin‒TCF4 heterotrimer complex. **A** Scatter plot of DEGs. **B** Overlay of downregulated genes (TRIB3-knockdown vs. control) with known suppressor genes in ferroptosis and upregulated genes (TRIB3-knockdown vs. control) with known driver genes in ferroptosis (up), respectively. Overlay of 12 down DEGs and 6 up DEGs with Cistrome Data Browser ChIP-seq (bottom). **C**, **D** qPCR to detect ALOXE3 and PARP8 levels in TRIB3 knockdown and control cells. **E**, **F** ChIP analyses of TCF4 binding on the ALOXE3 and PARP8 promoter in Cal27 TRIB3-overexpression cells. **G** Schematic illustration of wild-type (Wt) and mutant (Mut) sequences of one putative binding sites of TCF4 on ALOXE3 promoter are shown. **H** Transcriptional activity of ALOXE3 in Cal27 TCF4-knockdown or overexpression cells calculated by the luciferase reporter system. **I** Western blot to identify ALOXE3 and TCF4 protein level in Cal27 TCF4-knockdown or overexpression cells. ***P < 0.001, **P < 0.01, *P < 0.05.

The mechanism of TRIB3 inhibition in sensitizing ferroptosis was further investigated. RNA-seq and DEGs analysis were utilized for the exploration of the TRIB3 downstream signal (Fig. 6A). A total of 424 upregulated DEGs and 1289 downregulated DEGs met the criteria (|log2FC | ≥1 and FDR < 0.05) upon TRIB3-knockdown (Fig. 6B). To narrow down the focus on ferroptosis-related genes, the FerrDB database (http://www.zhounan.org/ferrdb/) was utilized to retrieve genes associated with ferroptosis. Subsequently, we proceeded to discern the intersection of DEGs upon TRIB3 knockdown and ferroptosis-related genes, resulting in the identification of 12 downregulated and 6 upregulated DEGs associated with ferroptosis (Fig. 6B).

As a transcription factor, TCF4 possesses both transcriptional activation and transcriptional repression functions [16, 17]. Hence, exploring downstream transcriptional targets associated with ferroptosis within the context of the TRIB3-β-catenin-TCF4 heterotrimeric complex becomes particularly intriguing. The ChIP-seq data, which were collected in Cistrome Data Browser (http://cistrome.org/db), was checked. It was finally identified that TCF4 may transcriptionally regulate the expression of the 4 down DEGs (NR5A2, TRIB2, PARP8, and TP63), as well as 1 upregulated DEG (ALOXE3) (Fig. 6B). To validate the impact of TRIB3 knockdown on these five DEGs, qPCR experiments were performed. The results demonstrated a significant upregulation of ALOXE3 mRNA level in TRIB3-knockdown cells, whereas only the mRNA level of PARP8 was found to be downregulated when compared to control cells (Fig. 6C, D).

**Fig. 7:**
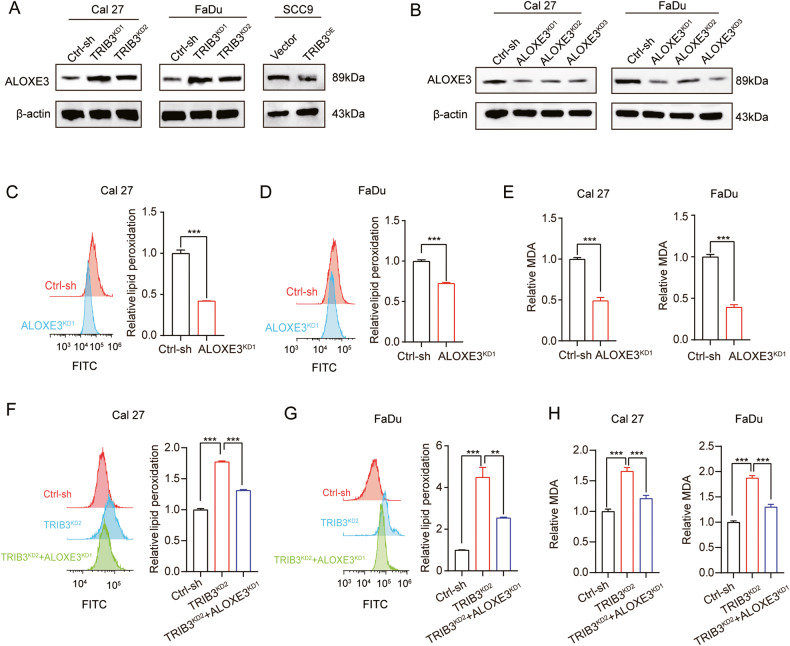
TRIB3‒β-catenin‒TCF4 heterotrimer complex negatively modulates ferroptosis by targeting ALOXE3. (**A**) Western blot to identify ALOXE3 protein level in TRIB3-knockdown or overexpression and control cells. (**B**) Western blot to measure ALOXE3 protein level in ALOXE3-knockdown and control cells. (**C**, **D**) Lipid peroxidation in ALOXE3-knockdown and control cells. (**E**) MDA in ALOXE3-knockdown and control cells. (**F**, **G**) Lipid peroxidation in HNSCC cells was transfected with TRIB3 and ALOXE3 shRNA. (**H**) MDA in HNSCC cells was transfected with TRIB3 and ALOXE3 shRNA. ***P < 0.001, **P < 0.01, *P < 0.05.

Subsequently, it was determined that the heterotrimeric complex could bind to ALOXE3 and PARP8 promoters for transcription regulation. By means of sequence analysis of the ALOXE3 and PARP8 promoter regions, three conserved TCF4-binding sites were identified within the central promoter region (Fig. 6E). To validate the direct binding of TCF4 to the promoter regions of ALOXE3 and PARP8, a ChIP-qPCR assay was conducted. This analysis was performed in TRIB3-overexpressing Cal27 cells using an anti-TCF4 antibody. The results indicated that TCF4 could specifically bind to the ALOXE3 one promoter region, but no such binding was observed in the case of the PARP8 promoter region (Fig. 6E, F). Next, a series of luciferase reporter vectors containing the full-length wild-type (WT) or mutant (Mut) ALOXE3 promoter sequences were constructed (Fig. 6G). A luciferase reporter analysis was conducted for further demonstration of TCF4 regulatory action on ALOXE3 promoter. Remarkably, silencing TCF4 increased ALOXE3 promoter luciferase activities, which was attenuated in the TCF4 overexpression cells (Fig. 6H). The findings were further confirmed by Western blot analysis, which showed that silencing TCF4 and TRIB3 increased the expression of ALOXE3, while TCF4 and TRIB3-overexpression attenuated its expression (Figs. 6I and 7A). These results suggested that TRIB3‒β-catenin‒TCF4 heterotrimer complex binds directly with and exerts inhibitory effects on ALOXE3 promoter activation.

ALOXE3 belongs to the family of lipoxygenases (including ALOX15B, ALOX15, ALOX12B, ALOX12, ALOX5, and ALOXE3 in mammalian cells) [18]. It can increase the lipid peroxides levels by enzymatic enhancement [19]. Next, we explored the role of ALOXE3 in ferroptosis. ALOXE3-knockdown HNSCC cells were generated, as depicted in Fig. 7B. Additionally, it was first confirmed that ALOXE3-knockdown inhibited lipid peroxidation and MDA in HNSCC cells. Notably, Fe^2+^ levels showed no significant change (Fig. 7C–E and Fig. S5a). Further investigations were conducted to ascertain that TRIB3 inhibits ferroptosis by targeting ALOXE3. Detection of cellular lipid peroxidation and MDA level was conducted. The data showed that silencing TRIB3-mediated augment of MDA and lipid peroxidation were relieved by ALOXE3 deficiency, but the augment of Fe^2+^ has not changed (Fig. 7F–H and Fig. S5b). Simultaneously, we were curious about the association between the level of ALOXE3 and prognosis in each cancer. We conducted a pan-cancer Kaplan–Meier survival analysis for ALOXE3 and found that it serves as a better prognostic indicator in glioblastoma multiforme (GBM), while it is associated with unfavorable outcomes in colon adenocarcinoma (COAD) and uterine corpus endometrial carcinoma (UCEC) (Fig. S6). Together, these compelling findings strongly suggested that the TRIB3‒β-catenin‒TCF4 heterotrimeric complex may target ALOXE3 in mediating ferroptosis in HNSCC cells.

### Suppression of cell growth, migration in HNSCC by TRIB3 inhibitor Hesperidin

**Fig. 8:**
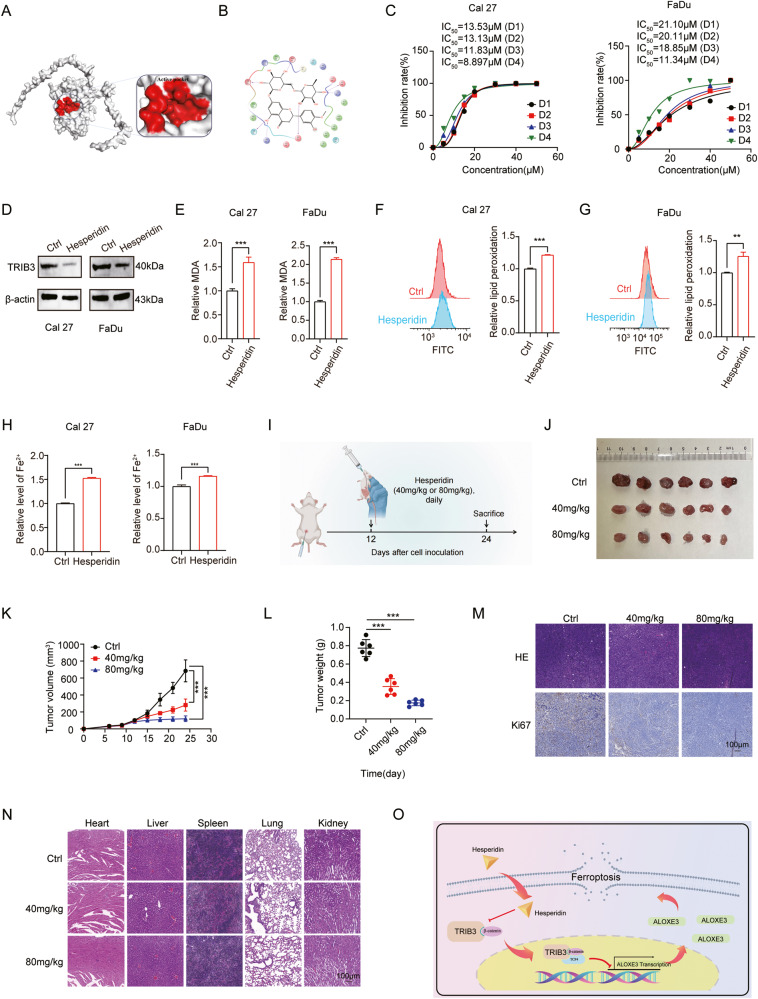
Hesperidin suppresses HNSCC initiation and progression. **A** The active pocket of TRIB3 protein. **B** The structure of hesperidin. **C** Cell viability of HNSCC cells treated with different Hesperidin concentrations. **D** Western blot to detect TRIB3 protein level in HNSCC cells treated with hesperidin (20 μm) for 48 h. **E** MDA in HNSCC cells treated with hesperidin (20 μm) for 48 h. **F**, **G** Lipid peroxidation in HNSCC cells treated with hesperidin (20 μm) for 48 h. **H** The level of Fe^2+^ in HNSCC cells treated with hesperidin (20 μm) for 48 h. **I** Schematic of FaDu subcutaneous tumors treated with hesperidin, **J**, **K** tumor growth and **L** weight in the xenograft model. **M** The expression of Ki67 using IHC assay. **N** H&E staining of the spleen, heart, kidney, lung, and liver tissues. Scale bar: 100 μm. **O** The schematic diagram illustrates the inhibition of ferroptosis in HNSCC by the TRIB3-β-catenin-TCF4 complex through the suppression of ALOXE3 transcription. ***P < 0.001, **P < 0.01, *P < 0.05.

From the aforementioned findings, it was deduced that TRIB3 may act as a ferroptosis-negative regulator and a carcinogenic gene. Therefore, the development of a TRIB3 inhibitor for targeting HNSCC treatment represents a potential novel advancement. Extensive research on compounds was conducted using molecular docking techniques, leading to the successful identification of the active site of the TRIB3 protein, which was defined as the docking pocket (Fig. 8A). Compounds were further screened based on PAINS structure and ADME. Ranked by their docking scores value, we selected hesperidin for further investigation in this study. The 2D structure diagram showed the force acting on Hesperidin (Fig. 8B).

Hesperidin, first isolated from orange peels in 1828, is abundantly present in various citrus fruits [20]. Indeed, it can influence different molecular targets associated with division, survival, and tumor cells death mechanisms. To evaluate the antitumor potential, different concentrations of hesperidin were treated with the HNSCC cells. Remarkably, hesperidin dose-dependently suppressed cell viability (Fig. 8C and Fig. S7a), induced cell apoptosis (Fig. S7b), and inhibited cell migration (Fig. S7c) in HNSCC cell lines. Additionally, it was confirmed that hesperidin contributed to a decrease in TRIB3 expression (Fig. 8D).

Moreover, hesperidin demonstrated the ability to promote ferroptosis in HNSCC cells (Fig. 8E–H). Next, the examination was conducted to find the antitumor effects of hesperidin in vivo. The results revealed that hesperidin significantly inhibited malignant growth in a concentration-dependent manner in a subcutaneous xenograft model (Fig. 8I–M). Importantly, there was no visible pathological damage in the liver, kidney, spleen, lung, or heart of the mice treated with or without Hesperidin (Fig. 8N). All these results revealed that hesperidin is a safe and promising lead compound for treating HNSCC.

## Discussion

The prediction of the prognosis of patients with HNSCC remains inadequate despite substantial progress attained in tumor immunotherapy and selected targeted medicines, necessitating the development of new therapeutic strategies [1, 21]. Prior research have illuminated the tumor-promoting effect of TRIB3 in various tumors [8–12]. Nevertheless, the function of TRIB3 proteins in HNSCC remains undiscovered. As depicted in Fig. 8O, we demonstrated the potential of TRIB3 as a prognostic indicator for poor outcomes in HNSCC, unveiling a novel mechanism by which it promotes tumor growth through the inhibition of ferroptosis. TRIB3 potential as a poor prognosis factor for HNSCC was demonstrated, and unveiled a novel mechanism by which it promotes tumor growth by inhibiting ferroptosis. TRIB3 recruits TCF4 and β-catenin to produce a heterotrimer complex in HNSCC cells, leading to reduce the transcriptional activity of ALOXE3 to attenuate ferroptosis in HNSCC cells. This discovery illuminates a hitherto unknown pathway influencing ferroptosis regulation in HNSCC. Additionally, a novel TRIB3 inhibitor, hesperidin, was further developed as an HNSCC therapeutic agent. Hesperidin inhibited cell growth and migration in a concentration-dependent manner, exhibiting effective antitumor properties. Treatment with hesperidin in HNSCC cells displayed a tendency to promote ferroptosis, akin to the effects observed with TRIB3 knockdown. Overall, our study reveals TRIB3 as a potential prognostic marker and a therapeutic target for HNSCC.

As a stress sensor, TRIB3 is highly expressed as a response to the endoplasmic reticulum, inflammatory, metabolic, and hypoxic stresses [6]. Resultantly, TRIB3 acts as a determinant of cell fate in numerous cancer types. Moreover, it influences the malignant characteristics of tumor cells by impacting multiple oncoproteins. Despite these insights into its role in cancer, the specific effect of TRIB3 on HNSCC has remained unclear. The data collected in our study revealed that low expression of TRIB3 in HNSCC cells leads to the inhibition of cell proliferation. Remarkably, consistent with these findings, the knockdown of TRIB3 significantly suppressed cancer development in a subcutaneous HNSCC xenograft tumor model.

Ferroptosis is a regulated cell death process hallmarked by the lethal peroxidation of lipids [22]. At the core of ferroptosis initiation is the reaction between H_2_O_2_ and labile iron through the Fenton reaction, leading to the peroxidation of phospholipids (PUFA-PLs) containing polyunsaturated fatty acids. This accumulation of lipid peroxidation on cellular membranes eventually results in membrane rupture and ferroptotic cell death [22, 23]. Recent studies have highlighted ferroptosis participation in the pathogenesis of multiple diseases [2, 24, 25]. These include ischemic injury, neoplastic and neurodegenerative diseases. Among them, the tumor-suppressor role of ferroptosis emerges as a promising therapeutic avenue for the treatment of malignancies. Interestingly, a recent study unveiled a significant finding that interleukin-6 (IL-6) has the potential to inhibit HNSCC ferroptosis while simultaneously promoting tumor progression [26]. This effect is achieved through the activation of xCT expression via the JAK2/STAT3 pathway. Surprisingly, our research revealed that TRIB3 attenuates the Fe^2+^ and cellular lipid peroxidation levels resulting in the inhibition of HNSCC cell ferroptosis. This observation implies that TRIB3 promotes HNSCC tumor growth by suppressing ferroptosis. Consequently, targeting TRIB3 to increase ferroptosis in HNSCC cells may effectively inhibit cancer progression.

The most attractive target of all for cancer therapy is the Wnt/β-catenin signaling [27–29]. After translocating from the cytoplasm to the nucleus, β-catenin binds to the TCF/LEF family, leading to the regulation of target gene transcription [30]. TCF4 plays a crucial role as the key transcription factor in the TCF/LEF family, serving as a pivotal switch in the Wntβ-catenin signaling pathway [31, 32]. The formation of the β-catenin-TCF4 complex represents the functional and ultimate stage in the activation of the canonical Wnt signaling to the β-catenin pathway. In our study, we found that TRIB3 can enhance the interaction by tethering the proteins of β-catenin and TCF4 together. We also observed that in control cells, the TRIB3 and TCF4 were co-localization in the nucleus and the nuclear expression of TCF4 does not decrease with the reduction of TRIB3, bur this does not alter their trimeric nature. This may be attributed to the formation of a complex between TRIB3 and β-catenin, which subsequently translocates to the cell nucleus and associates with TCF4, influencing the activation or inhibition of downstream gene transcription. When TRIB3 is reduced, the nuclear entry of the TRIB3 and β-catenin complex decreases, resulting in a corresponding reduction in the trimeric complex formed with TCF4. Additionally, following the reduction of TRIB3, both total and nuclear levels of β-catenin decreased, while the cytoplasmic level remained constant. We posit two potential explanations: (i) Silencing TRIB3 in HNSCC cells inhibits β-catenin expression and its nuclear translocation. Consequently, diminished nuclear translocation results in cytoplasmic accumulation, yielding cytoplasmic protein levels akin to those in the control group. (ii) β-catenin degradation primarily occurs via phosphorylation facilitated by the proteasome [33], we speculate that the gradual cytoplasmic accumulation of β-catenin may prompt an increase in its phosphorylation levels, leading to the degradation of excess β-catenin to maintain homeostasis. Further investigation is warranted to elucidate the underlying reasons for the consistent levels of cytoplasmic β-catenin.

Studies have shown a strong negative correlation between β-catenin and ferroptosis in cancer cells [15, 34, 35]. Consistently, our finding revealed that the TRIB3-β-catenin-TCF4 transcription complex directly associates with the promoter region of ALOXE3, inhibiting its expression and conferring resistance to ferroptosis. ALOXE3 belongs to the lipoxygenase family and modulates the cell sensitivity and PUFA status to ferroptosis by facilitating the cellular accumulation of lipid hydroperoxide [19, 36]. It has been depicted in multiple studies that deficiency of ALOXE3 can promote cancer cell survival and render certain cancer cells resistant to ferroptosis, including colorectal cancer (CRC), Hepatocellular carcinoma (HCC), and GBM cells [18, 37–39], and in the follow-up project, we will delve into a more detailed exploration of ALOXE3. Our study reveals that the role of ALOXE3 as a tumor suppressor can trigger ferroptosis and the TRIB3-β-catenin-TCF4 trimeric complex decreased ferroptosis by inhibiting the function of ALOXE3. Interestingly, it should be noted that the knockdown of ALOXE3 inhibits the level of lipid peroxidation but has no effect on Fe^2+^, as ALOXE3 primarily functions to increase lipid peroxide levels enzymatically, and it is not directly involved in iron metabolism. This suggests that other pathways modulated by TRIB3 may also inhibit ferroptosis in these models, requiring further investigation.

TRIB3 is critically involved in cell proliferation, ferroptosis, and cell survival, making it a promising drug target in HNSCC. However, thus far, no TRIB3 inhibitors have been proven successful for clinical HNSCC therapy. Thus, the development of effective and functional TRIB3 inhibitors for HNSCC patients is warranted. In this regard, a novel TRIB3 inhibitor, hesperidin, was developed as a potential therapeutic agent for HNSCC. Hesperidin, a natural ingredient with effectiveness against different cancer types and a history of use in traditional Chinese medicine, demonstrated efficacy against tumor progression in both in vitro and in vivo studies [40–43]. When treated with hesperidin, HNSCC cells exhibited a pro-ferroptosis phenotype, closely resembling TRIB3-knockdown effects. However, the bioavailability of hesperidin may affect its effectiveness as a phytochemical in vivo, as its weak water solubility can restrict absorption. Combining hesperidin with chemotherapeutic drugs may enhance its effectiveness. Additionally, the safety profile and long-term usage of hesperidin require further investigation, although multiple research studies have demonstrated its low risk and safety. The experimental work in this study showed hesperidin to be safe, with no toxic effects observed on the liver, heart, kidney, lung, or spleen. Nonetheless, comprehensive preclinical toxicity investigations are necessary before considering its application in clinical settings and drug development.

In conclusion, the study identifies TRIB3 as a tumor promoter in HNSCC via attenuating ferroptosis. Mechanistically, TRIB3 can form a trimeric complex by interacting with β-catenin-TCF4, suppressing ALOXE3 transcriptional activity and inhibiting ferroptosis. Furthermore, the investigation unveiled a novel potent TRIB3 inhibitor, hesperidin, capable of inducing ferroptosis and demonstrating antitumor effectiveness in HNSCC. The exploration of targeting ferroptosis through TRIB3 inhibition introduces new possibilities for treating HNSCC, warranting further investigation and the development of targeted therapies.

### Supplementary information

supplementary file

Original data file

aj-checklist

## Data Availability

The original contributions presented in the study are included in the article/Supplementary Material; further inquiries can be directed to the corresponding author.
