# Supplementary material for: TRIB3 promotes malignancy of head and neck squamous cell carcinoma via inhibiting ferroptosis
Source: Cell Death Dis. 2024 Mar 1;15(3):178. doi: 10.1038/s41419-024-06472-5 (PMC10907716; doi:10.1038/s41419-024-06472-5)
Supplement: Supplementary file 1 — supplementary file [file 41419_2024_6472_MOESM1_ESM.docx]

**
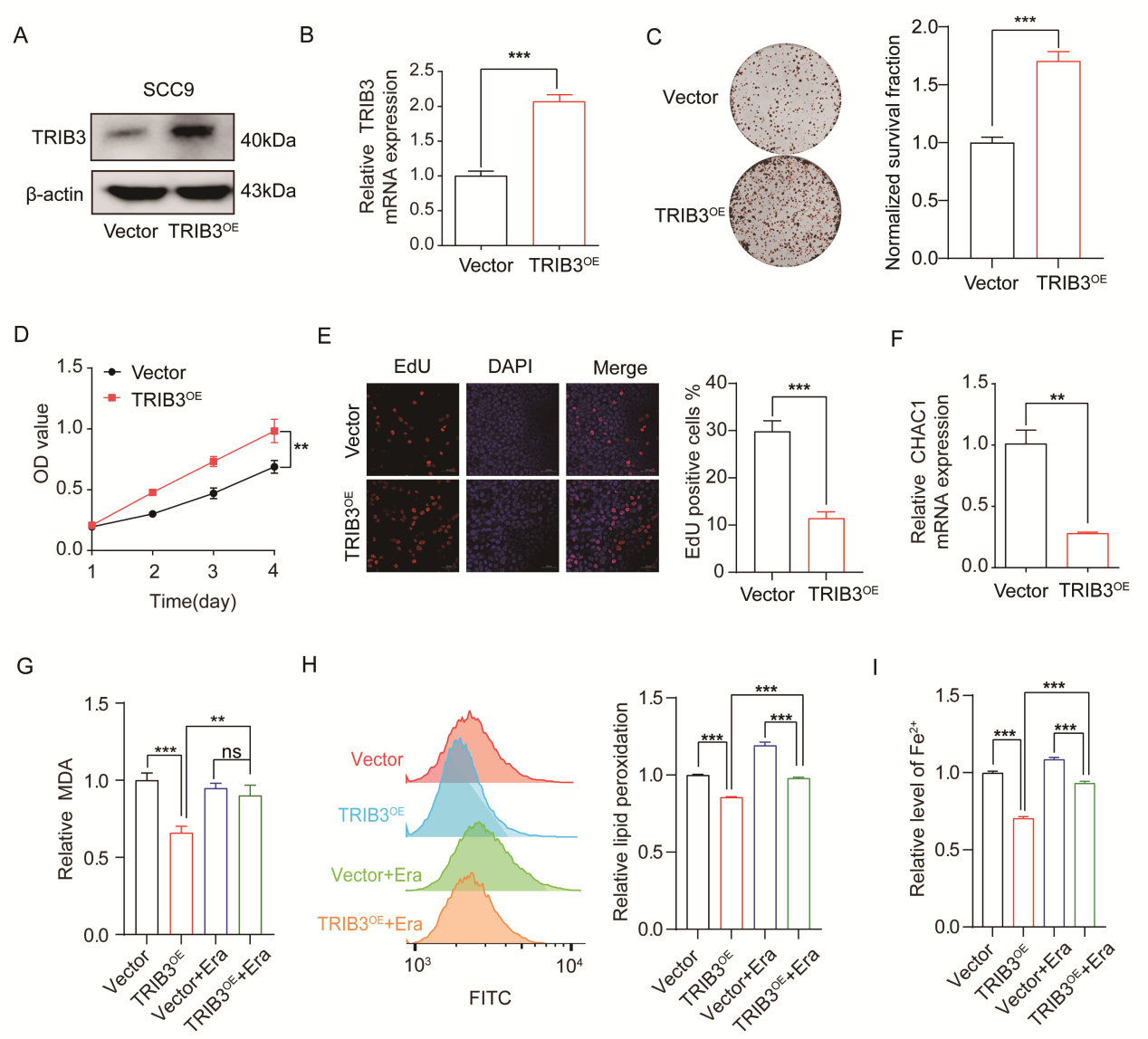
**

**Fig.S1.** Overexpressing of TRIB3 inhibits ferroptosis in HNSCC. (A) Western blot and qPCR (B) to detect TRIB3 levels in TRIB3-overexpression and Vector SCC9 cells. (C) Findings of a clonogenic assay. Red circles demonstrate the counted clones. (D) OD450 readings were plotted over time using the CCK8 assay. (E) Visualization of DNA replication using EdU. Red cell nuclei demonstrated DNA replication. Scale bar: 50 μm. (F) qPCR analysis of CHAC1 expression in Vector SCC9 cells and TRIB3-overexpression. (G) MDA in TRIB3-overexpression and Vector SCC9 cells treated with or without Erastin (10μm) for 48 hours. (H) Lipid peroxidation in TRIB3-overexpression and Vector SCC9 cells treated with or without Erastin (10μm) for 48 hours. (I) The level of Fe^2+^ in TRIB3-overexpression and Vector SCC9 cells treated with or without Erastin (10μm) for 48 hours. ****P*<0.001, ***P*<0.01, **P*<0.05.

**
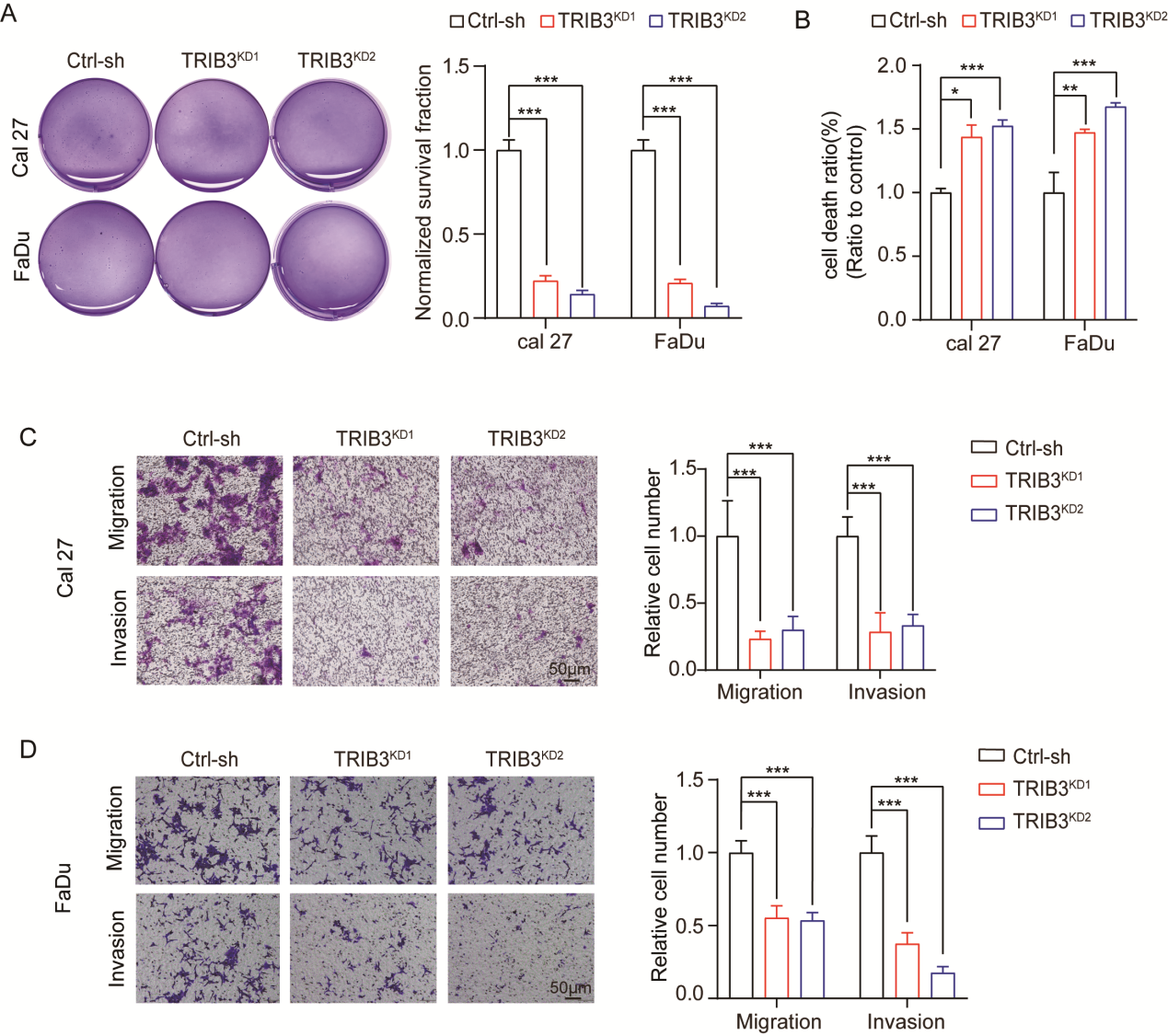
**

**Fig.S2.** TRIB3 silencing inhibits HNSCC cell proliferation and metastasis. (A) Soft agar colony formation assay was to detect cell proliferation. (B) PI staining was utilized to detect cell death. (C, D) Transwell assay was employed to assess cell migration and invasion. Scale bar: 50 μm. ****P*<0.001, ***P*<0.01, **P*<0.05.


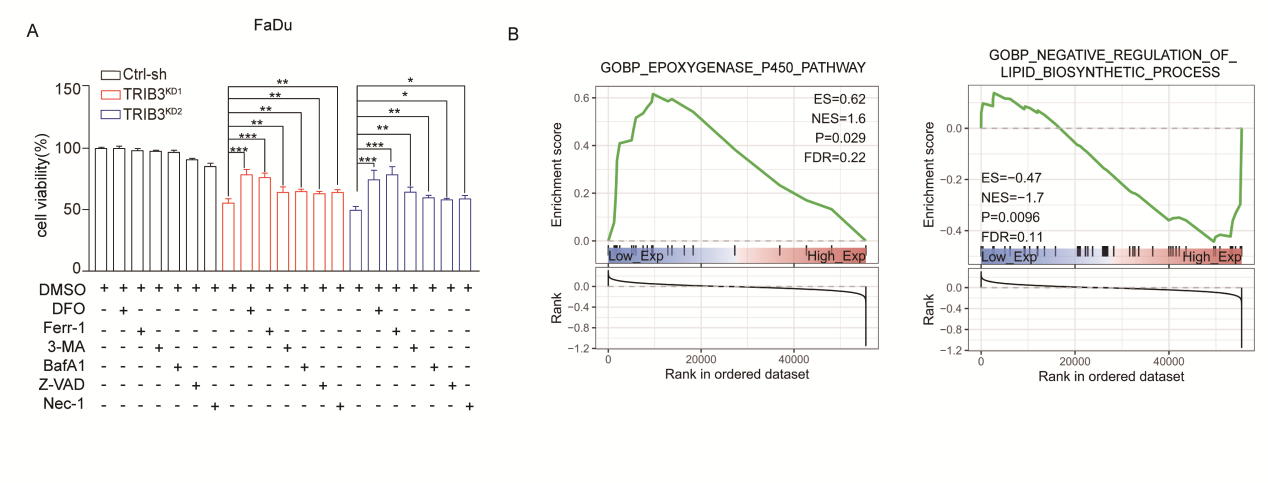


**Fig.S3.** TRIB3 silencing promotes cell death largely by inhibiting ferroptosis. (A) Detection of cell viability of control FaDu cells and TRIB3-knockdown, treated with or without cell death inhibitors, by CCK-8 assay. (B) GSEA enrichment plot revealing the enrichment of the gene linked to ferroptosis pathways. ****P*<0.001, ***P*<0.01, **P*<0.05.


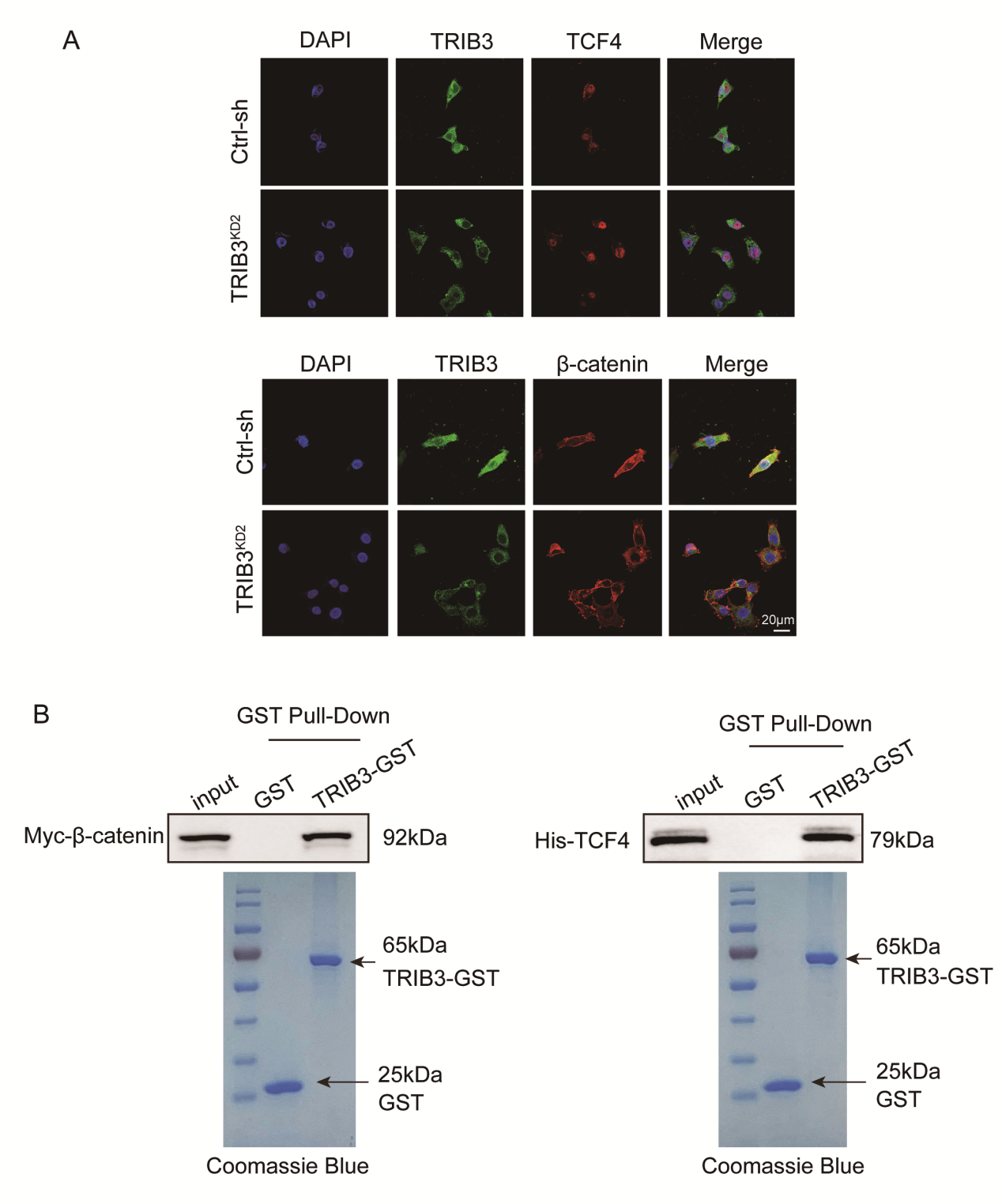


**Fig.S4.** Interaction of TRIB3 with β-catenin and TCF4 to create a heterotrimeric complex. (A) The co-localization of TRIB3 with TCF4 and β-catenin was examined using confocal microscopy (40x). (B) The in vitro interaction between TRIB3 and β-catenin (left), TRIB3 and TCF4 (right) were assessed through the GST Pull-Down assay. Scale bar: 20 μm. ****P*<0.001, ***P*<0.01, **P*<0.05.


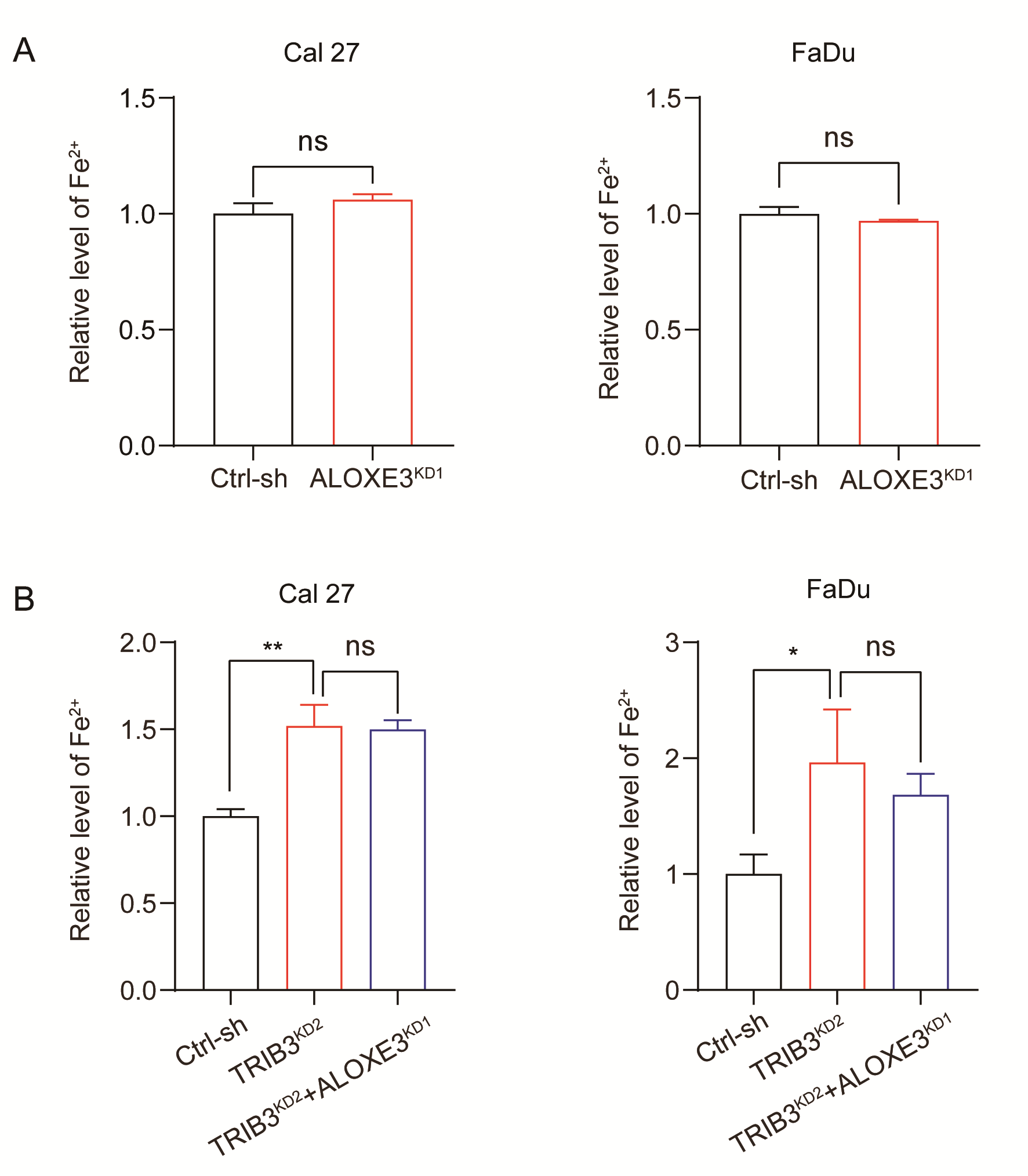


**Fig.S5.** ALOXE3 silencing has no effect on the level of Fe^2+^. (A) The level of Fe^2+^ in ALOXE3-knockdown and control cells. (B) The Fe^2+^ level in HNSCC cells was transfected with TRIB3 shRNA plus ALOXE3 shRNA. ****P*<0.001, ***P*<0.01, **P*<0.05.


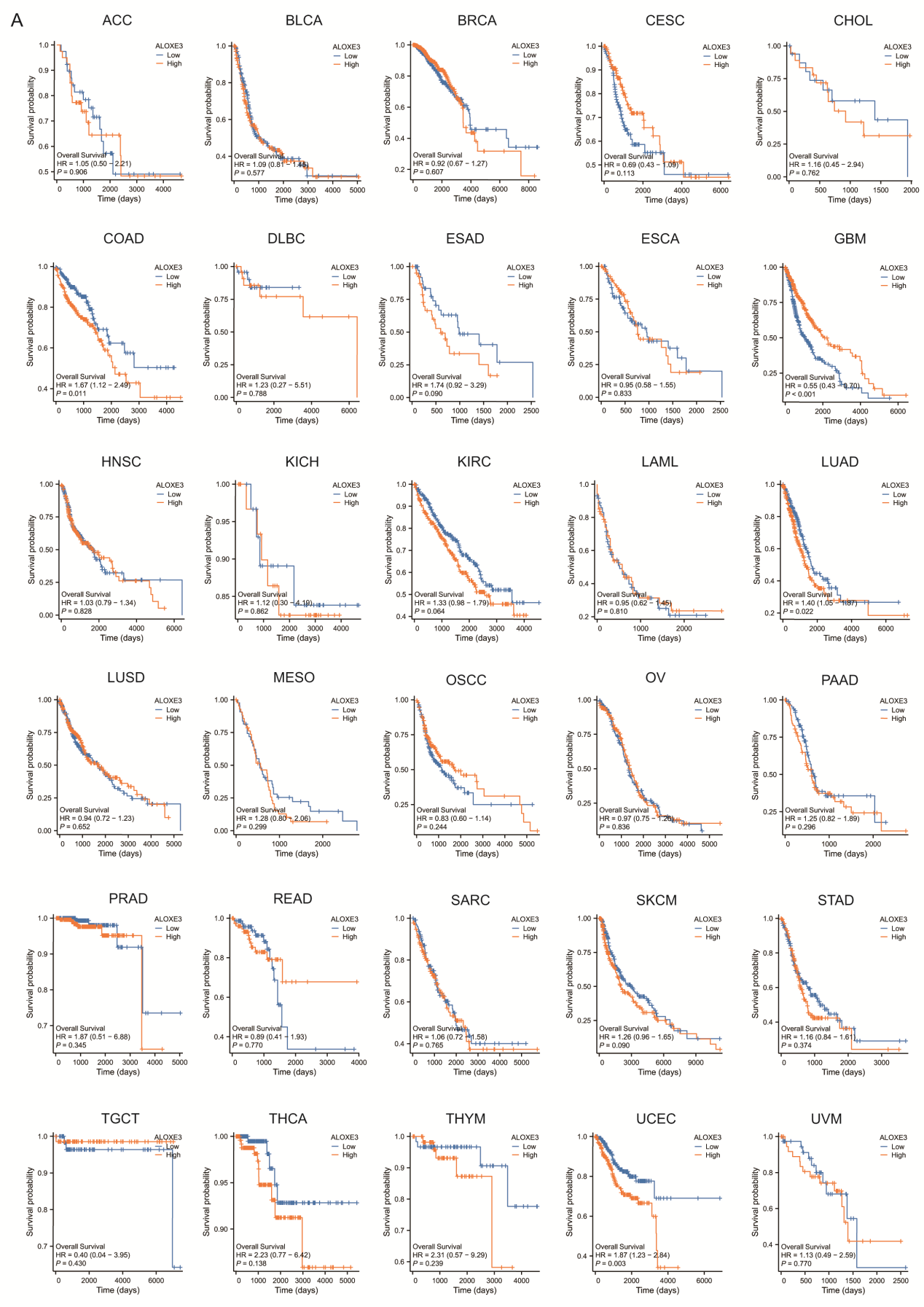


**Fig.S6.** Association between ALOXE3 expression and overall survival (OS). (A) Kaplan–Meier analysis of the association between ALOXE3 expression and OS.


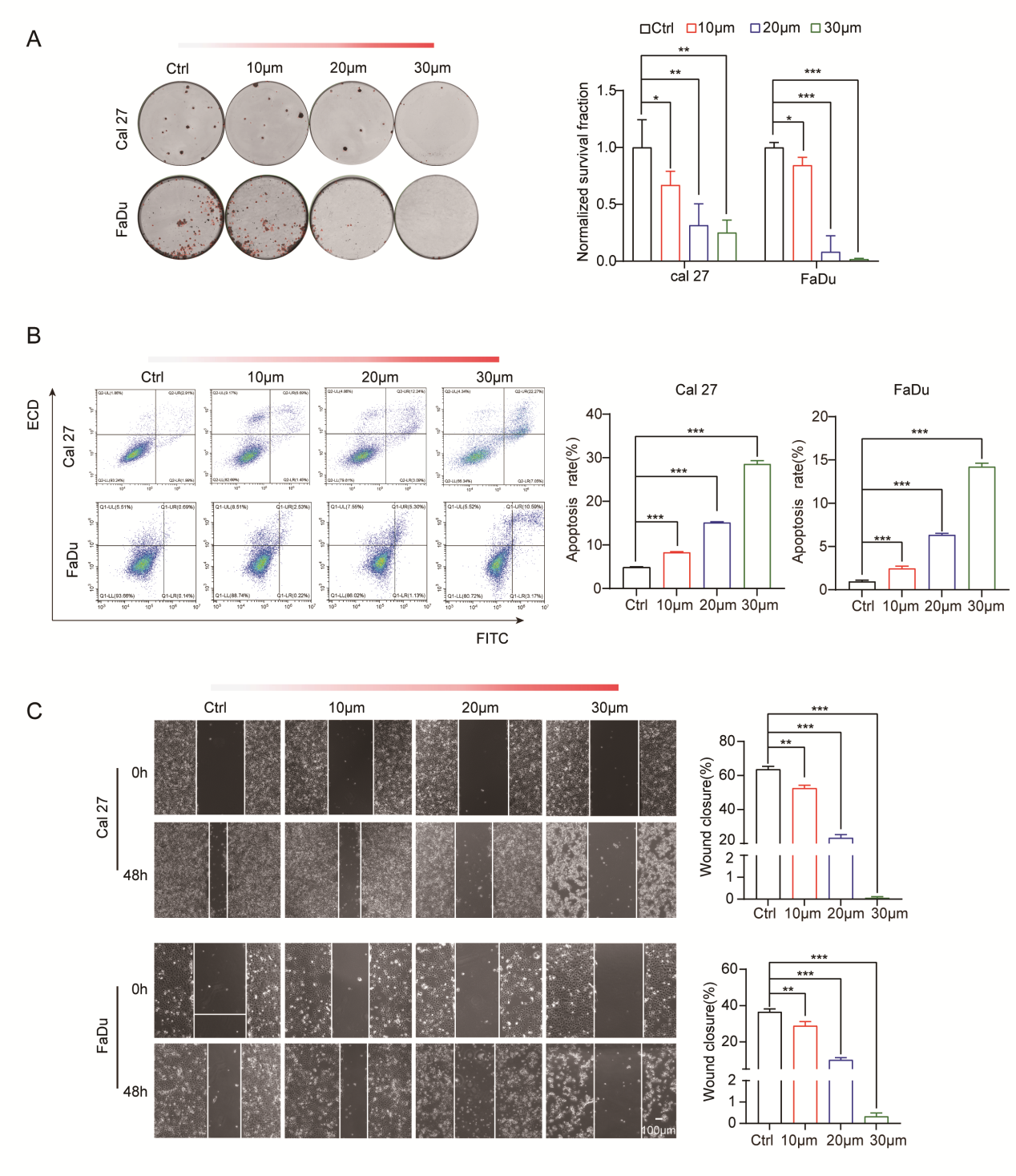


**Fig.S7.** Hesperidin suppresses HNSCC initiation and progression. (A) Findings of a clonogenic analysis. Red circles demonstrated the counted clones. (B) The apoptosis of HNSCC cells treated with different Hesperidin concentrations. (C) A scratch wound assay was utilized to detect the migratory capacities of HNSCC cells treated with different Hesperidin concentrations for 48 hours. Scale bar: 100 μm. ****P*<0.001, ***P*<0.01, **P*<0.05.
