# Supplementary material for: TRIB3 promotes malignancy of head and neck squamous cell carcinoma via inhibiting ferroptosis
Source: Cell Death Dis. 2024 Mar 1;15(3):178. doi: 10.1038/s41419-024-06472-5 (PMC10907716; doi:10.1038/s41419-024-06472-5)
Supplement: Supplementary file 2 — Original data file [file 41419_2024_6472_MOESM2_ESM.docx]

Fig.2a


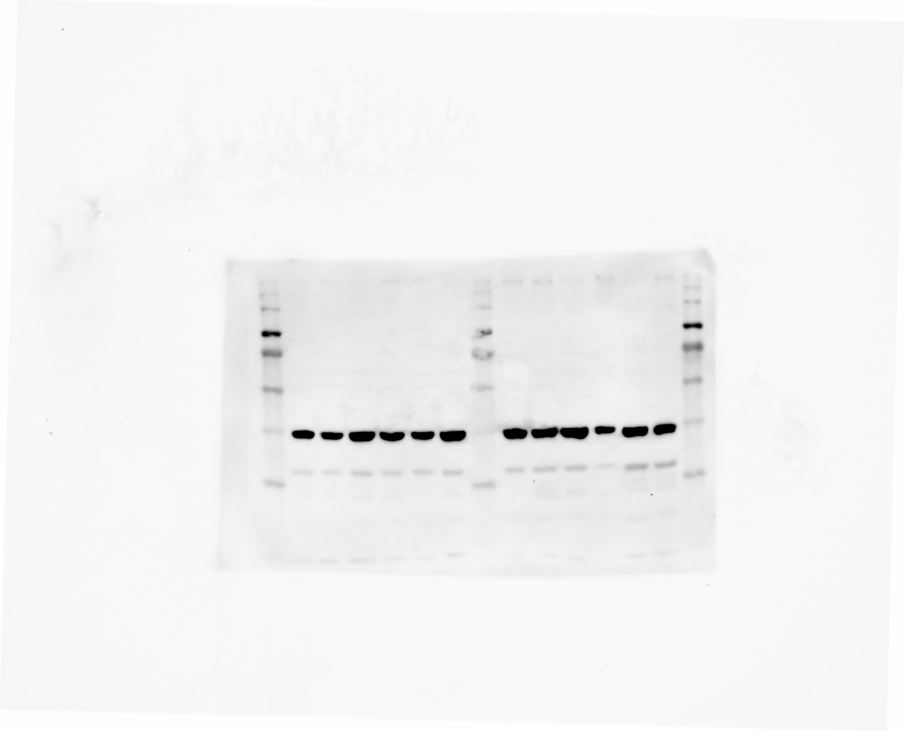

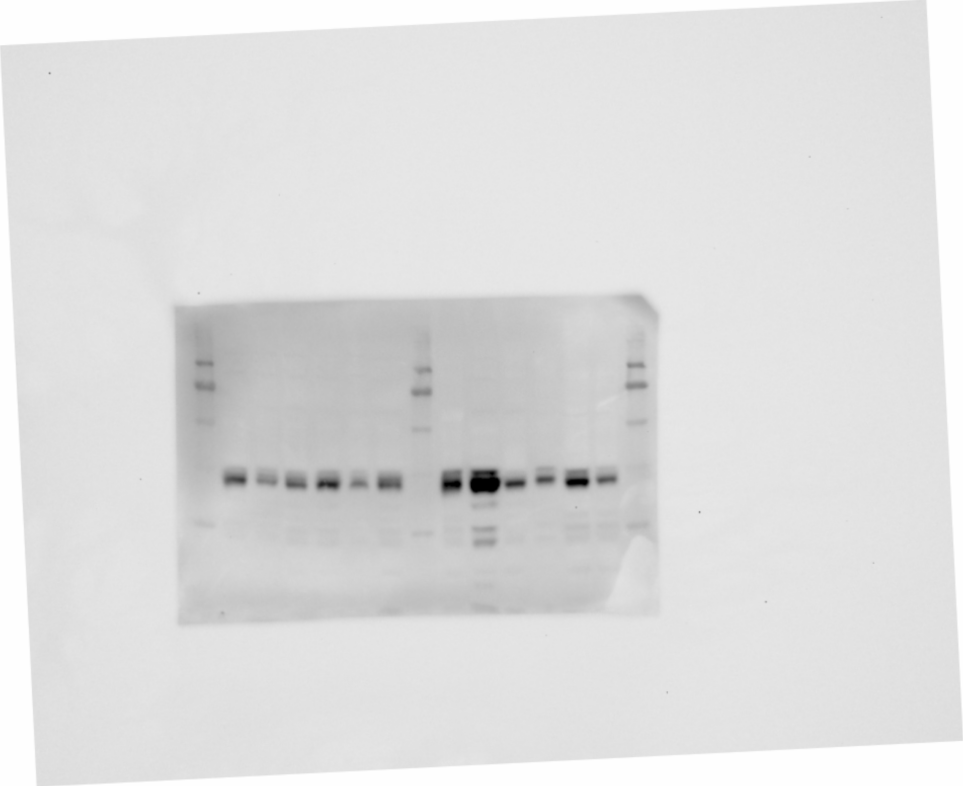


TRIB3

Trib3

β-actin

Fig.2b

FaDu

Cal 27


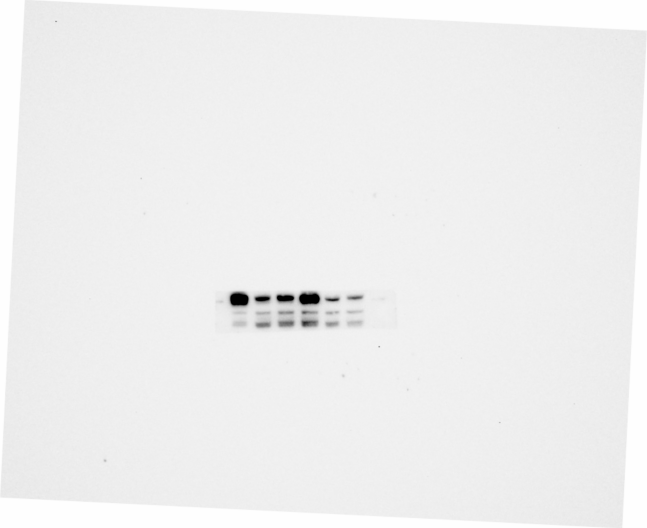

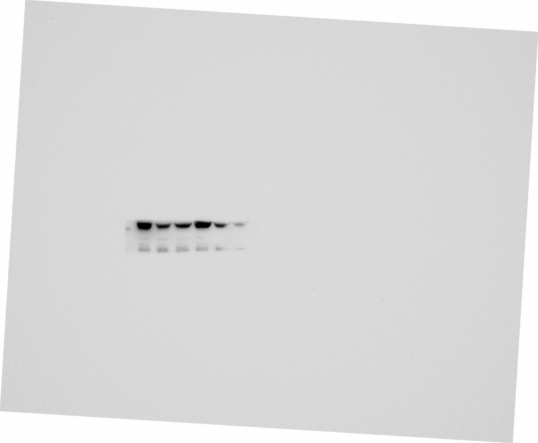

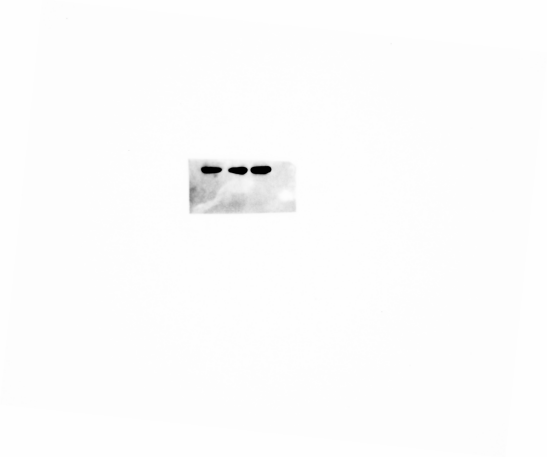

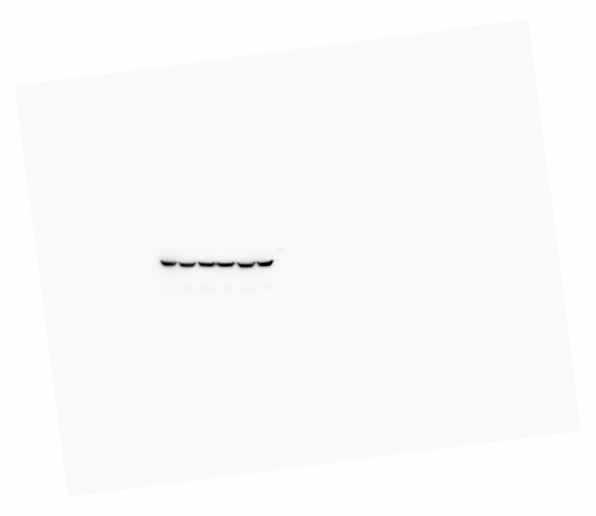


β-actin

Trib3

β-actin

Trib3

TRIB3

Trib3

TRIB3

Trib3

Fig.5b -Total

FaDu

Cal 27


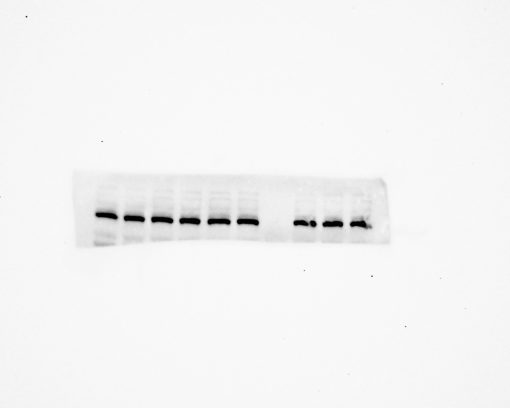

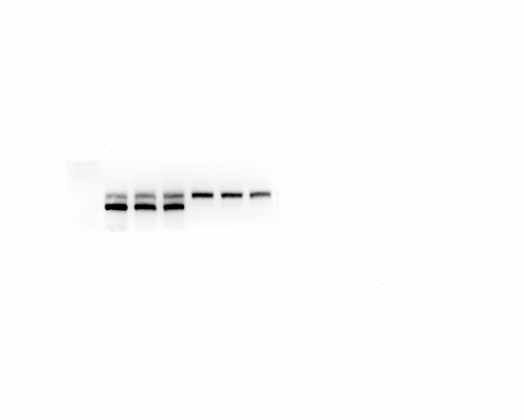


TCF4

TCF4


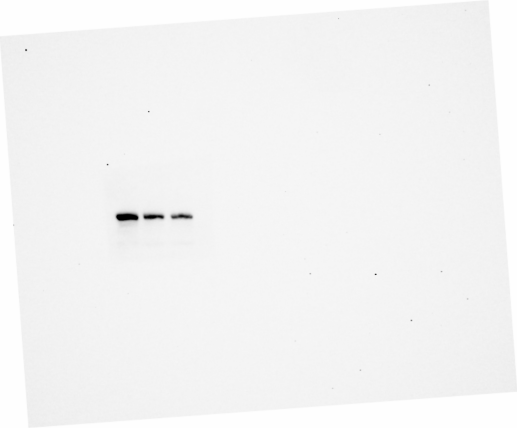

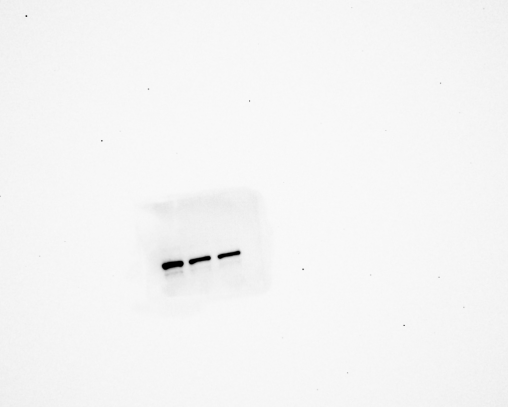


β-catenin

β-catenin


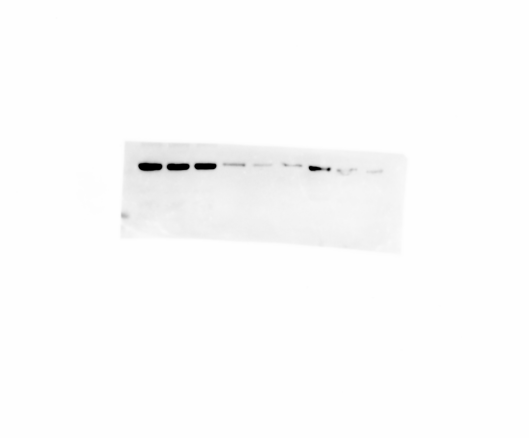

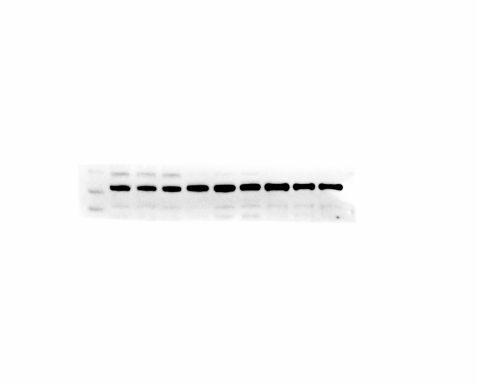


β-actin

β-actin

Fig.5b -cyto


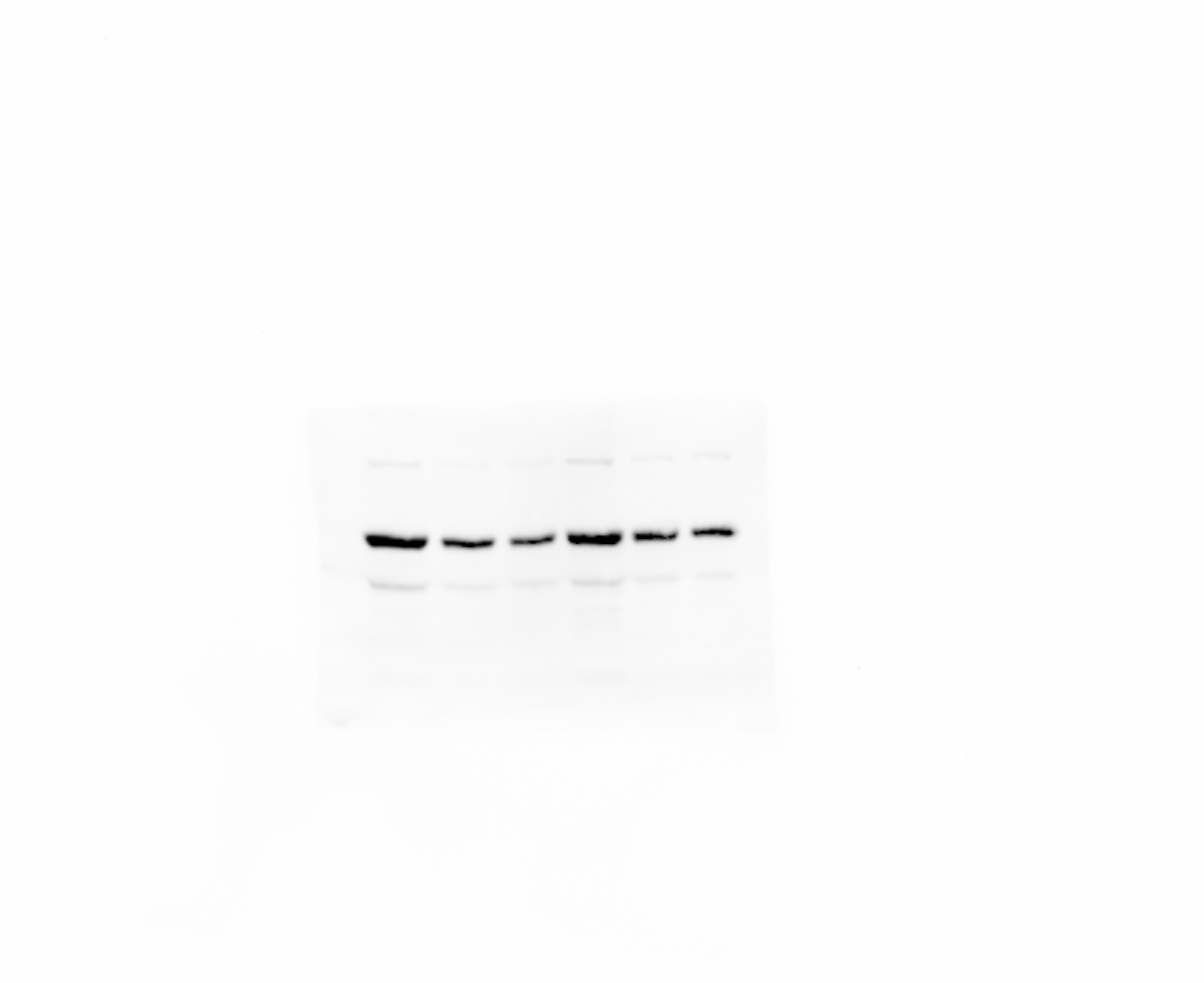

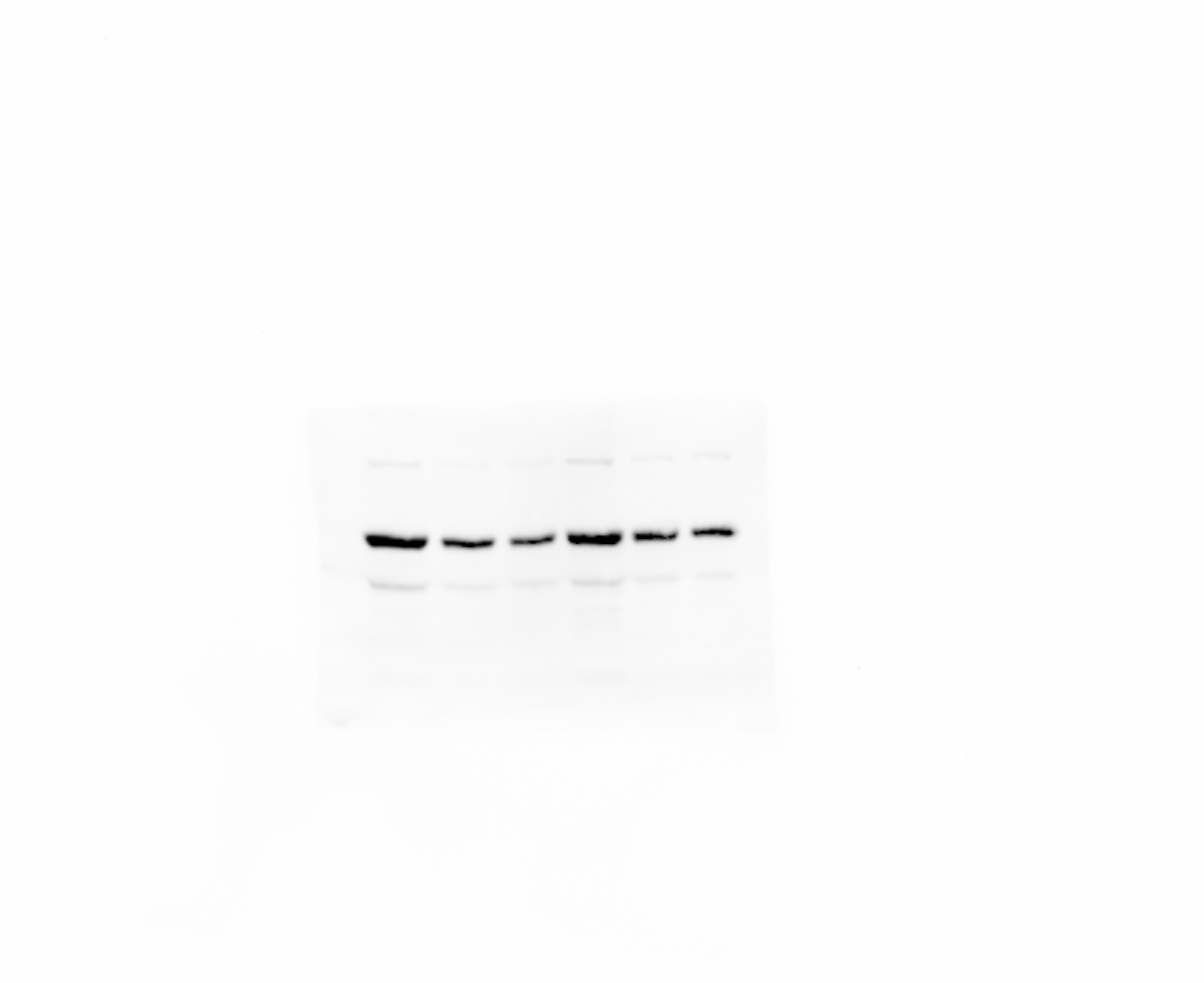


FaDu

Cal 27

TRIB3

TRIB3

TRIB3


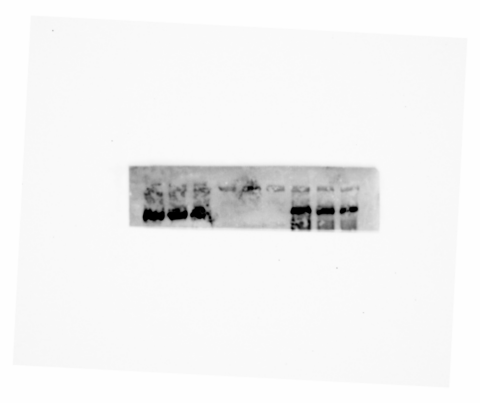

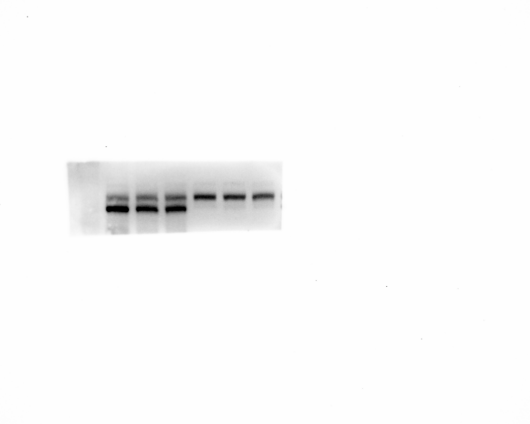


TCF4

TCF4


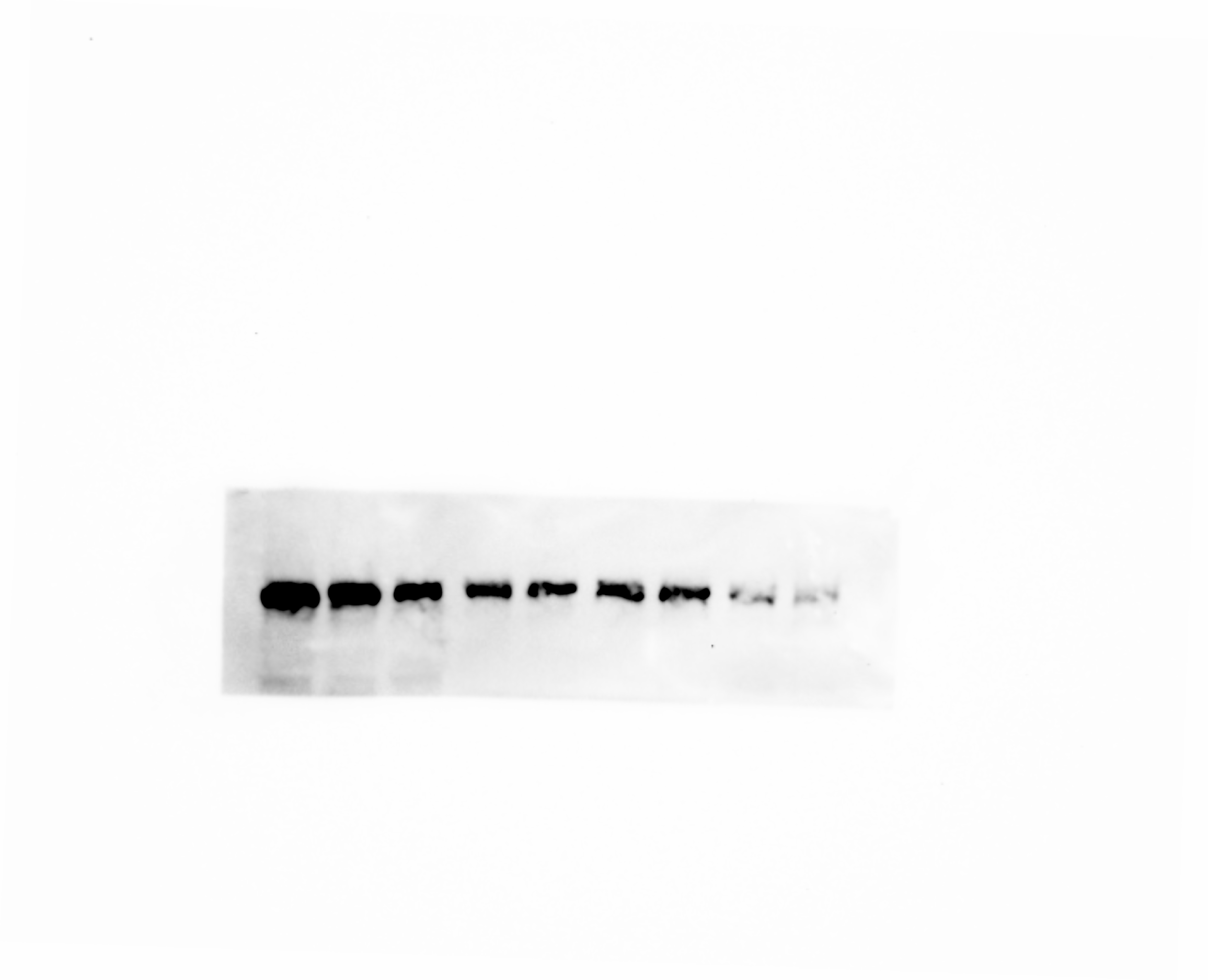

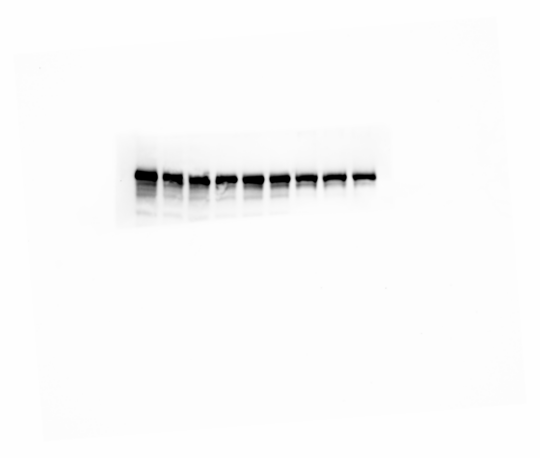


β-catenin

β-catenin


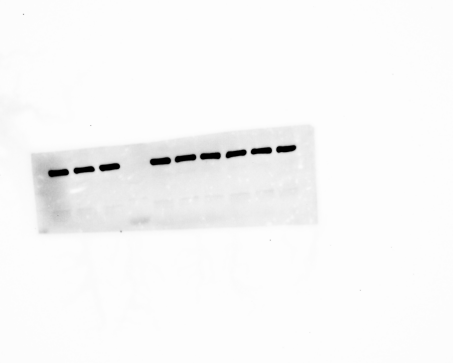

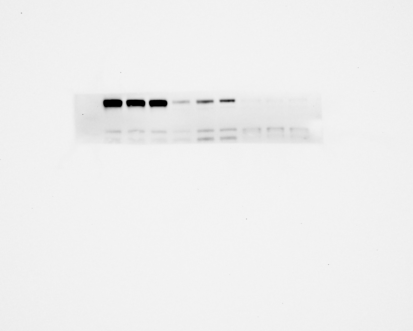


β-Tubulin

β-Tubulin

Fig.5b -nucleus

FaDu

Cal 27


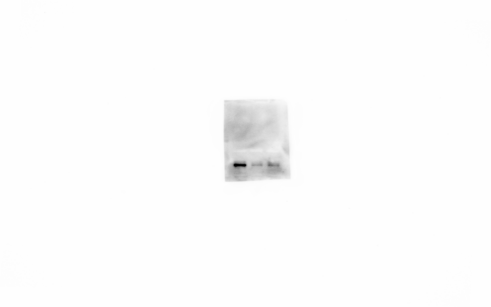

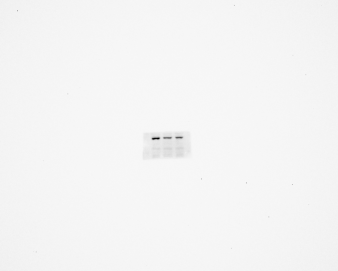


TRIB3

TRIB3


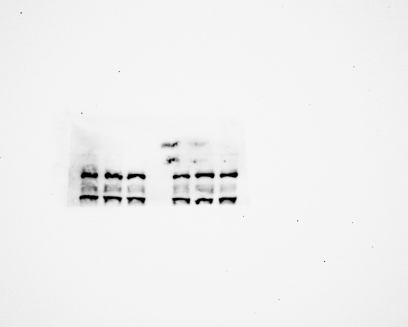

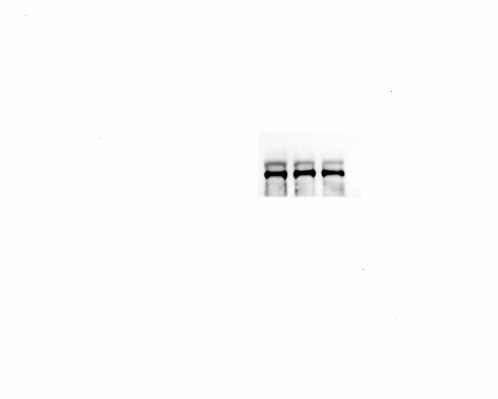


TCF4

TCF4


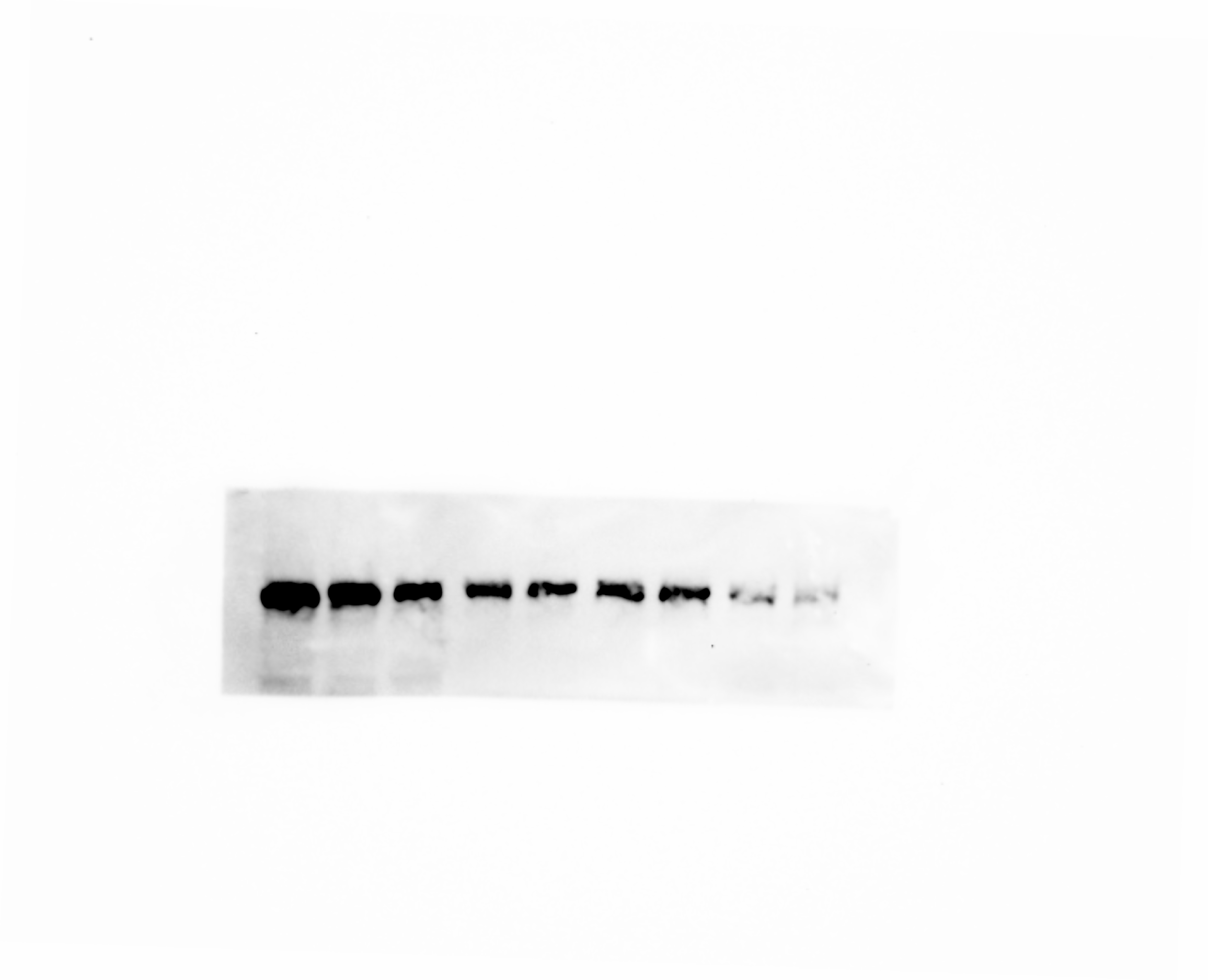

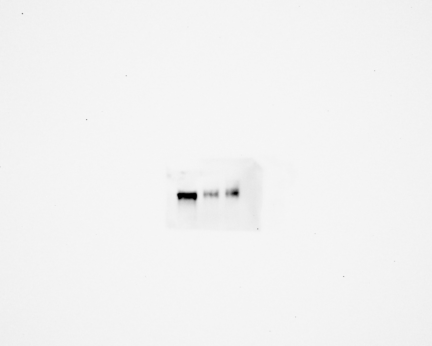


β-catenin

β-catenin

Histone-H3

Histone-H3

Fig.5c cal 27 Input


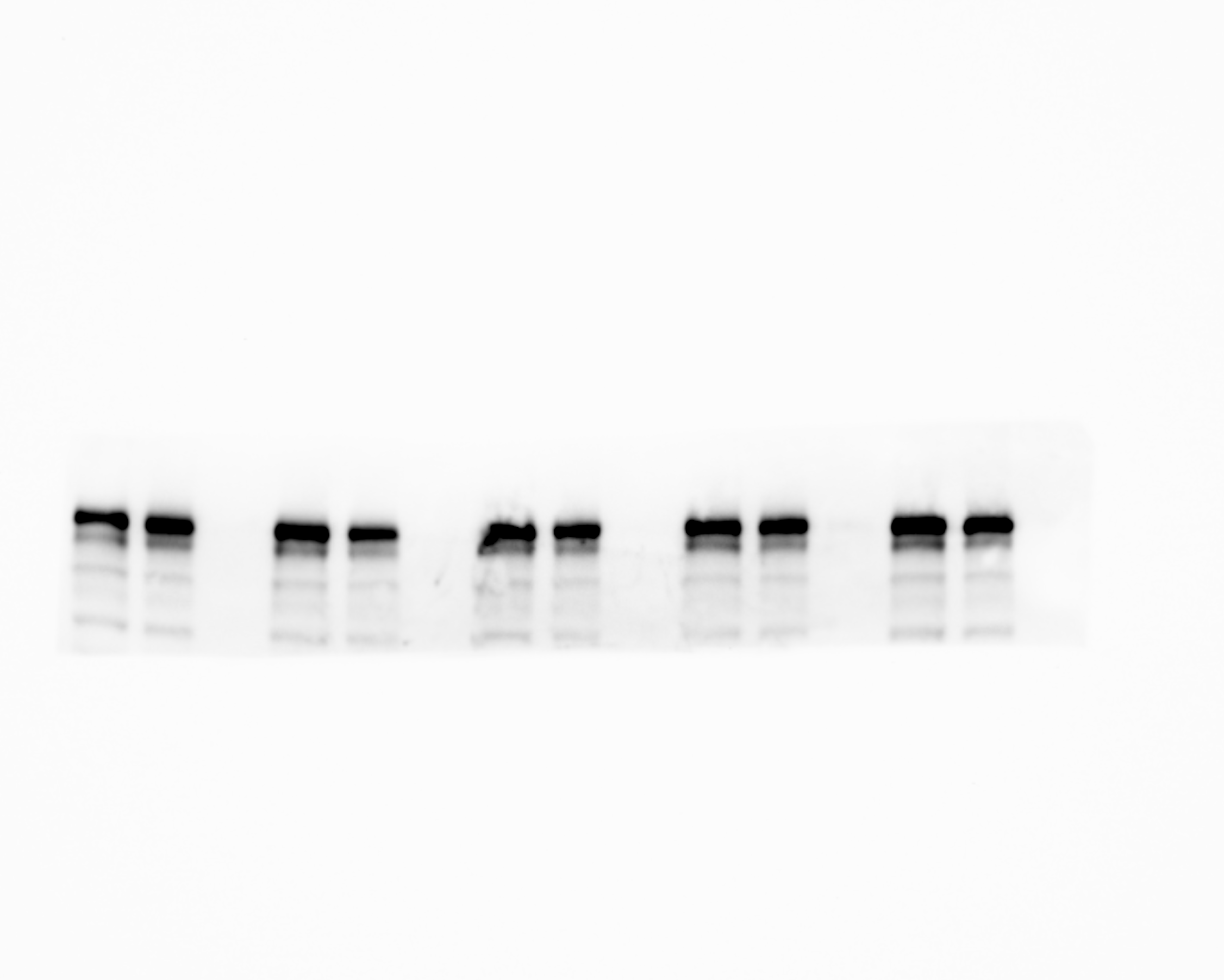

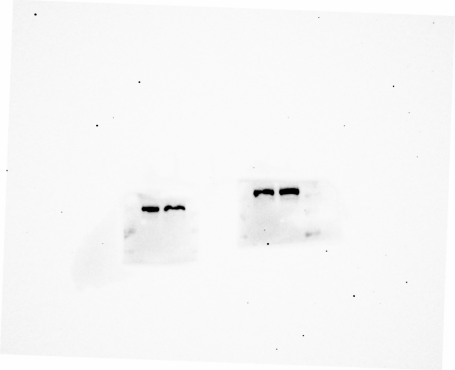


β-catenin

β-catenin


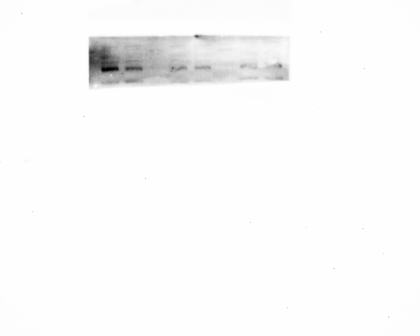

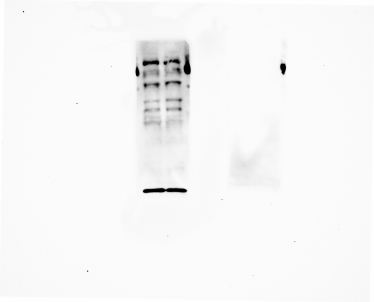

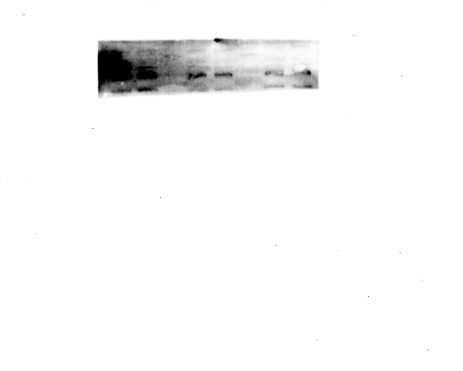


TCF4

TCF4

TCF4


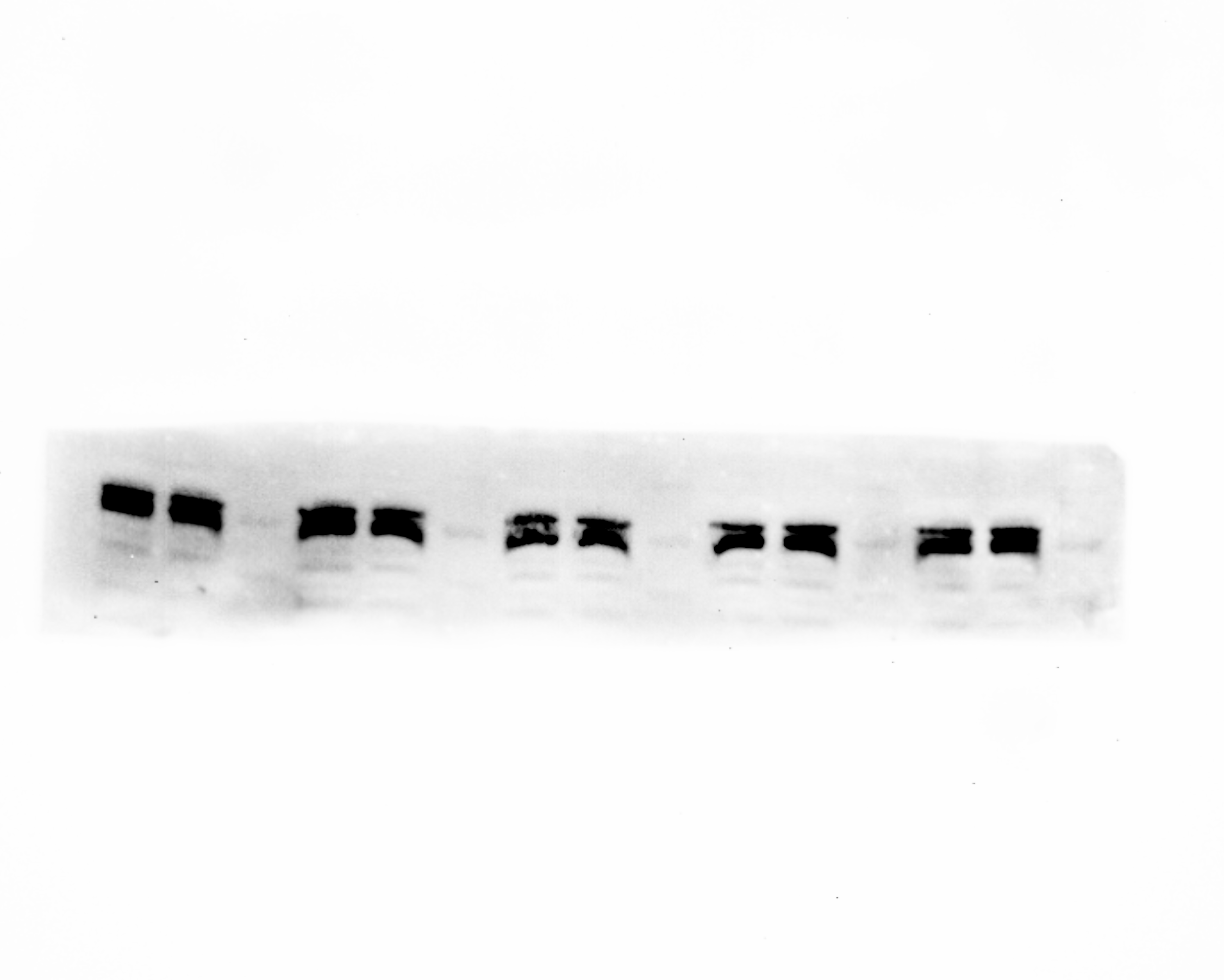


TRIB3


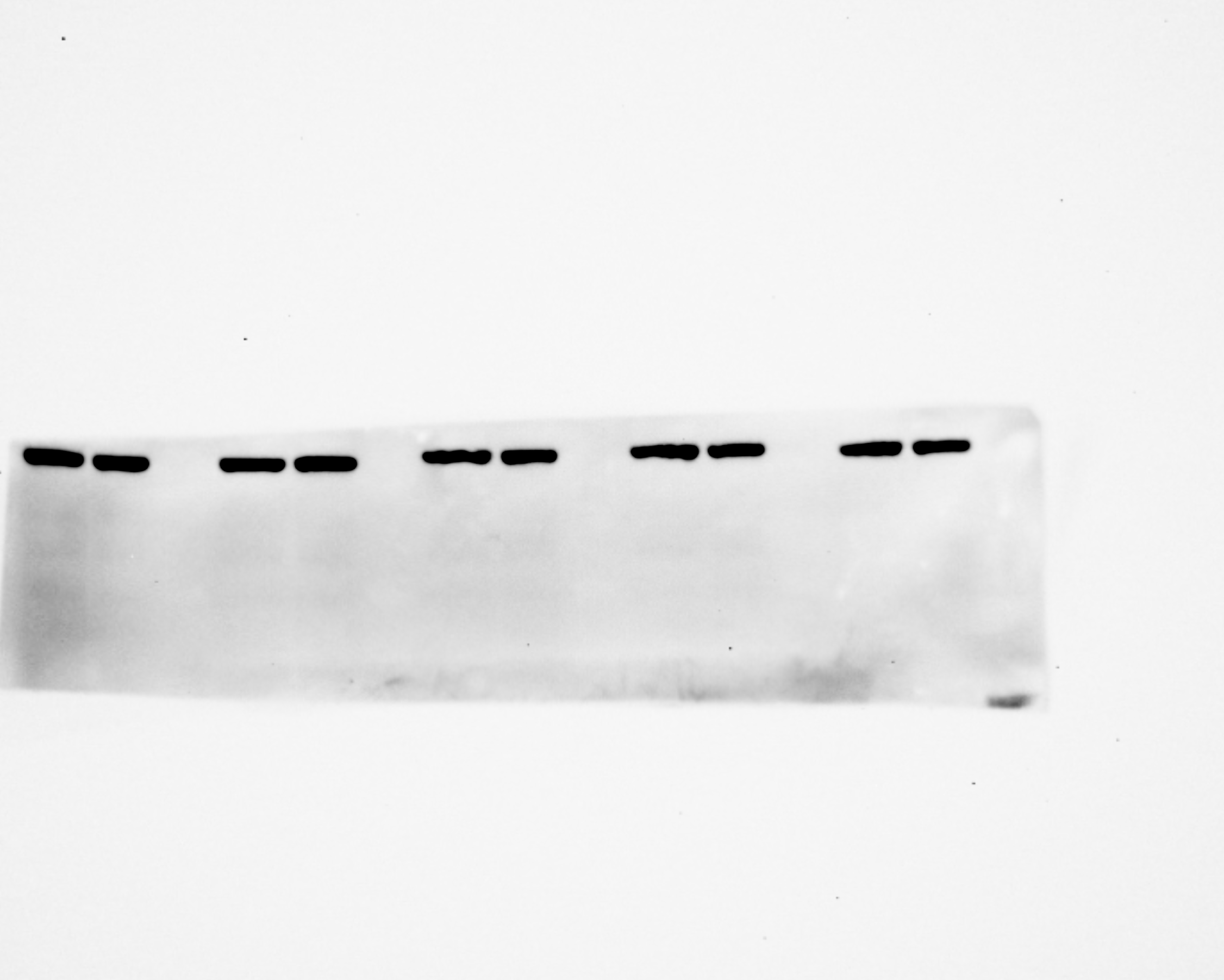

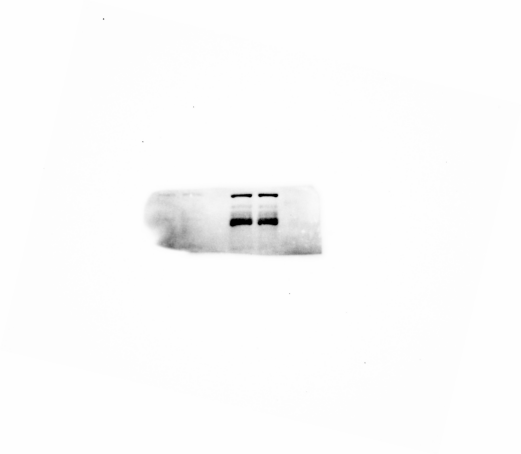


β-actin

β-actin

Fig.5c cal 27 IP


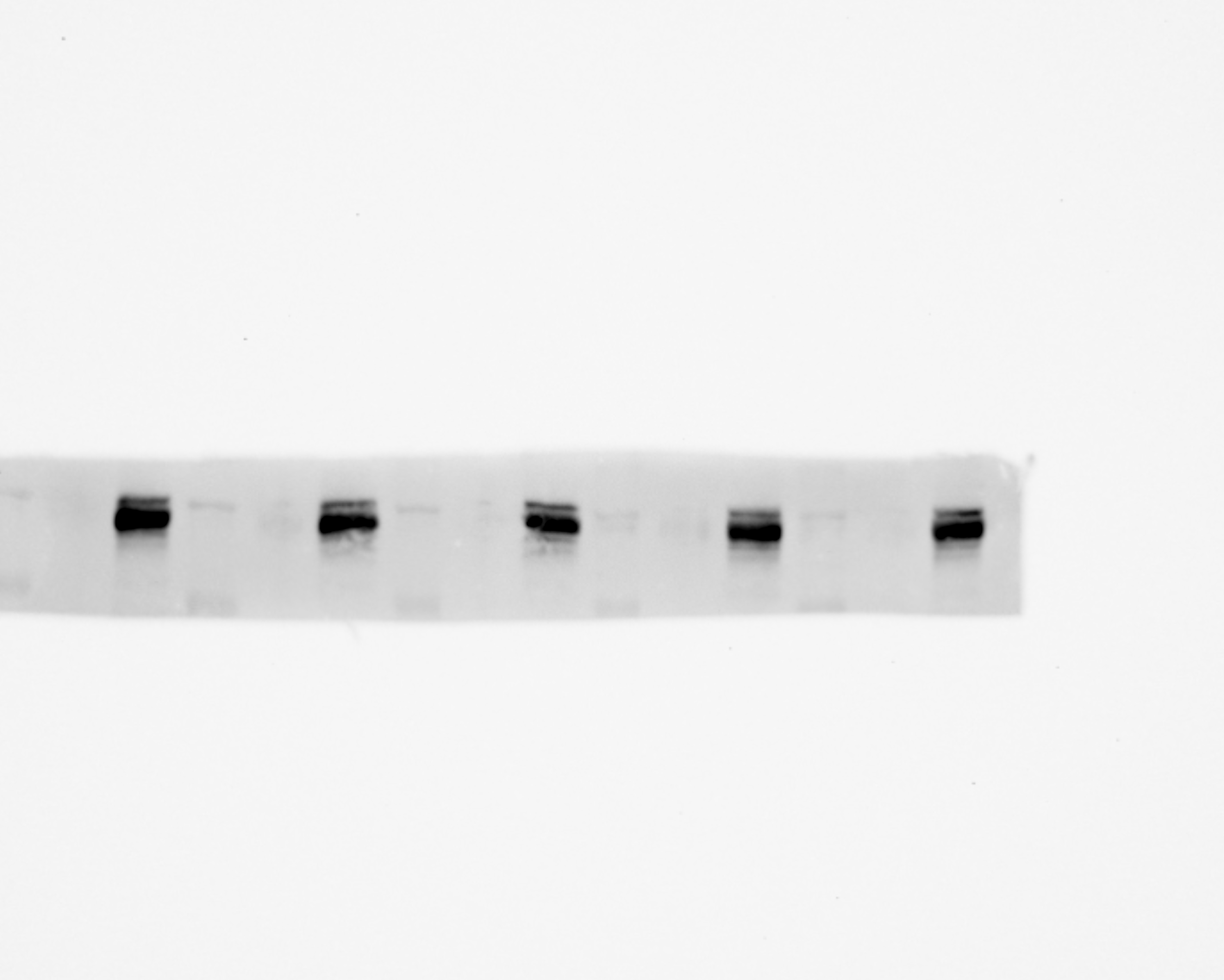


β-catenin


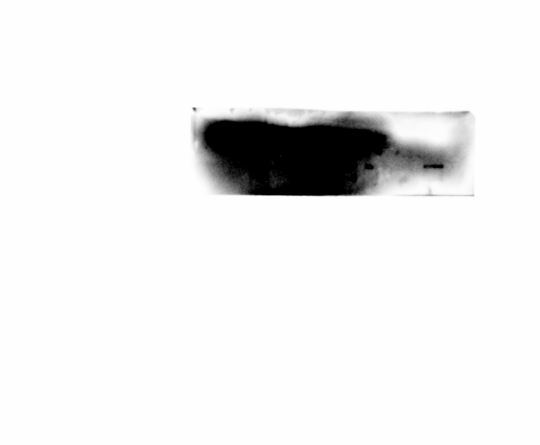

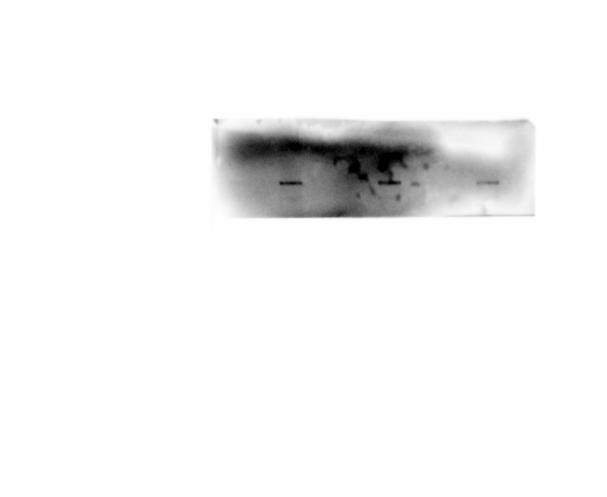


TCF4

TCF4


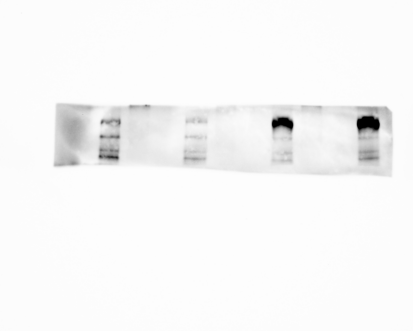

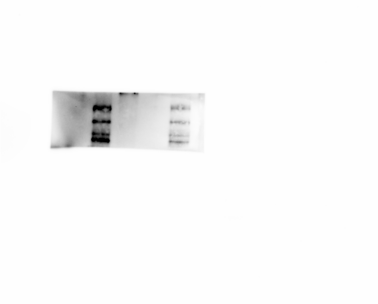

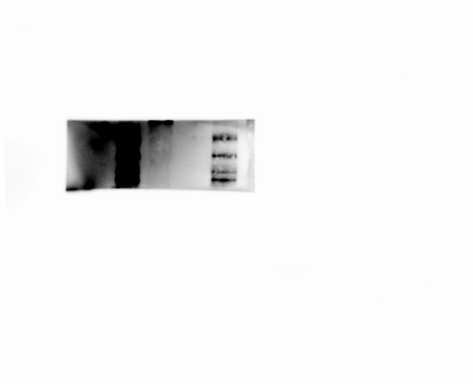


TRIB3

TRIB3

TRIB3

Fig.5d FaDu Input


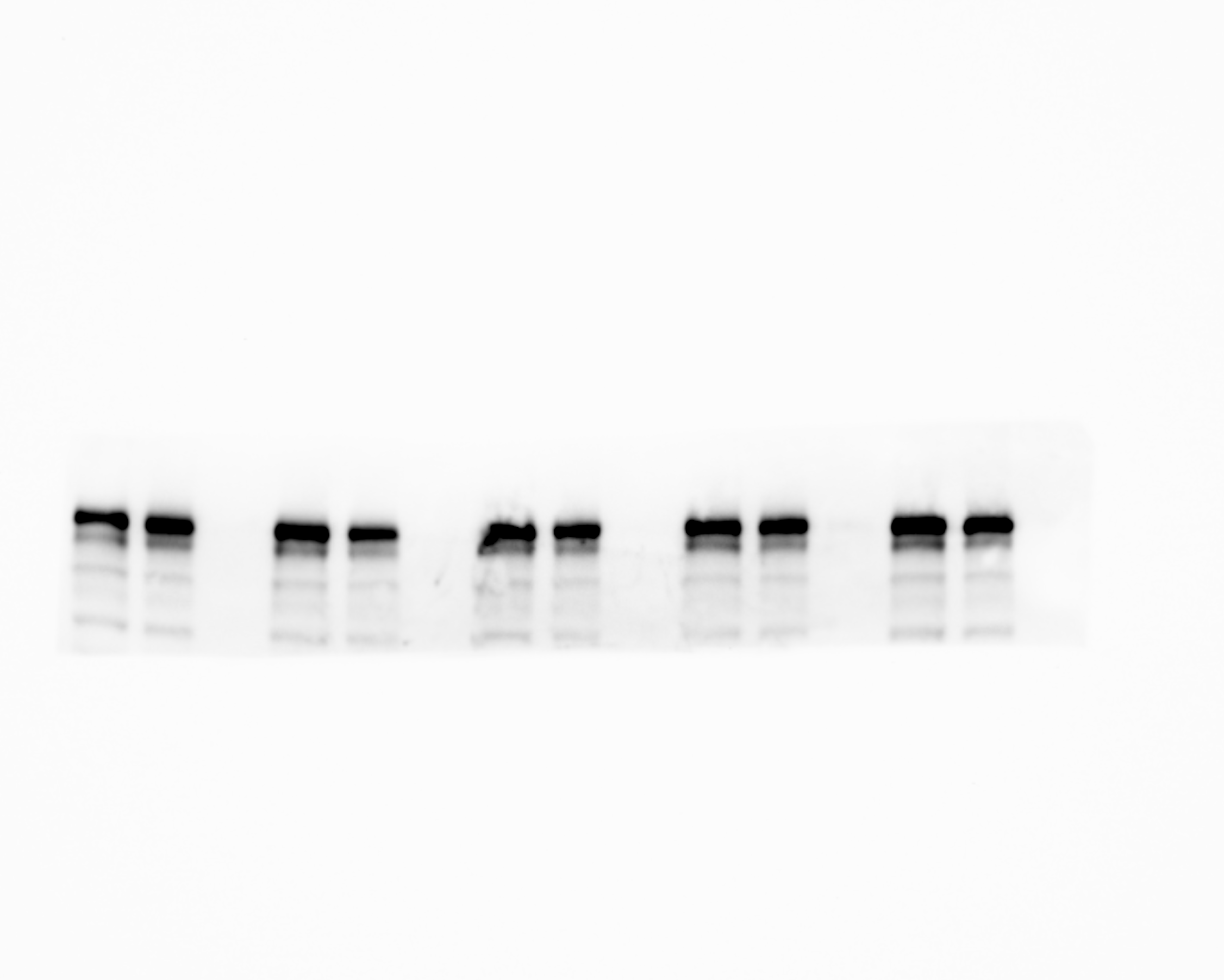


β-catenin


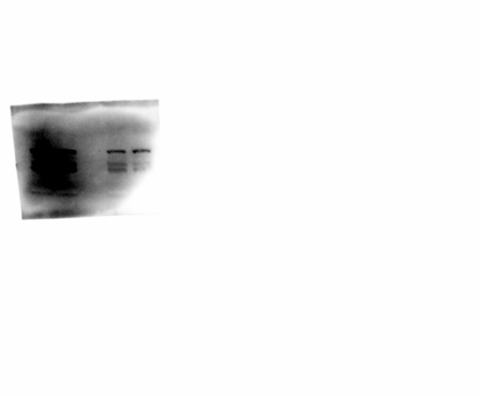

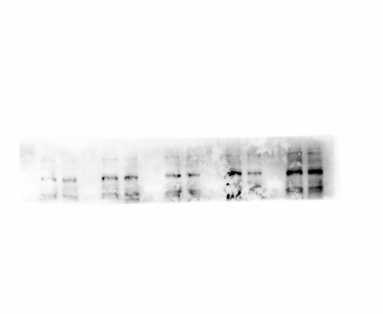

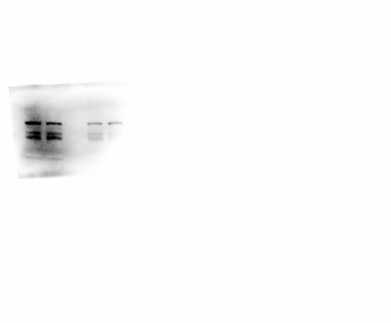


TCF4

TCF4

TCF4


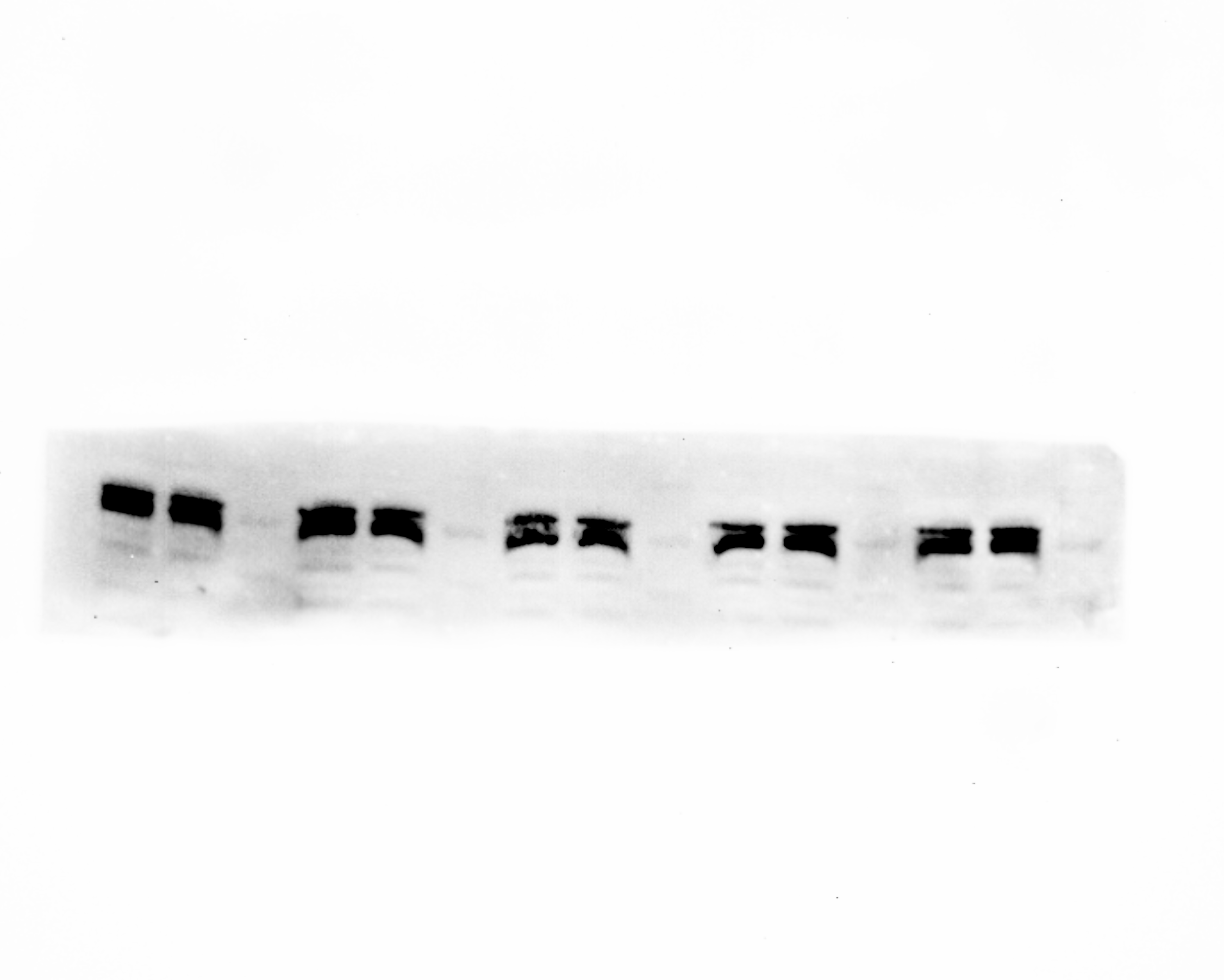

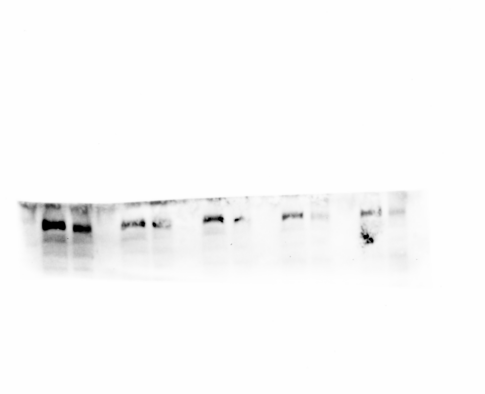


TRIB3

TRIB3


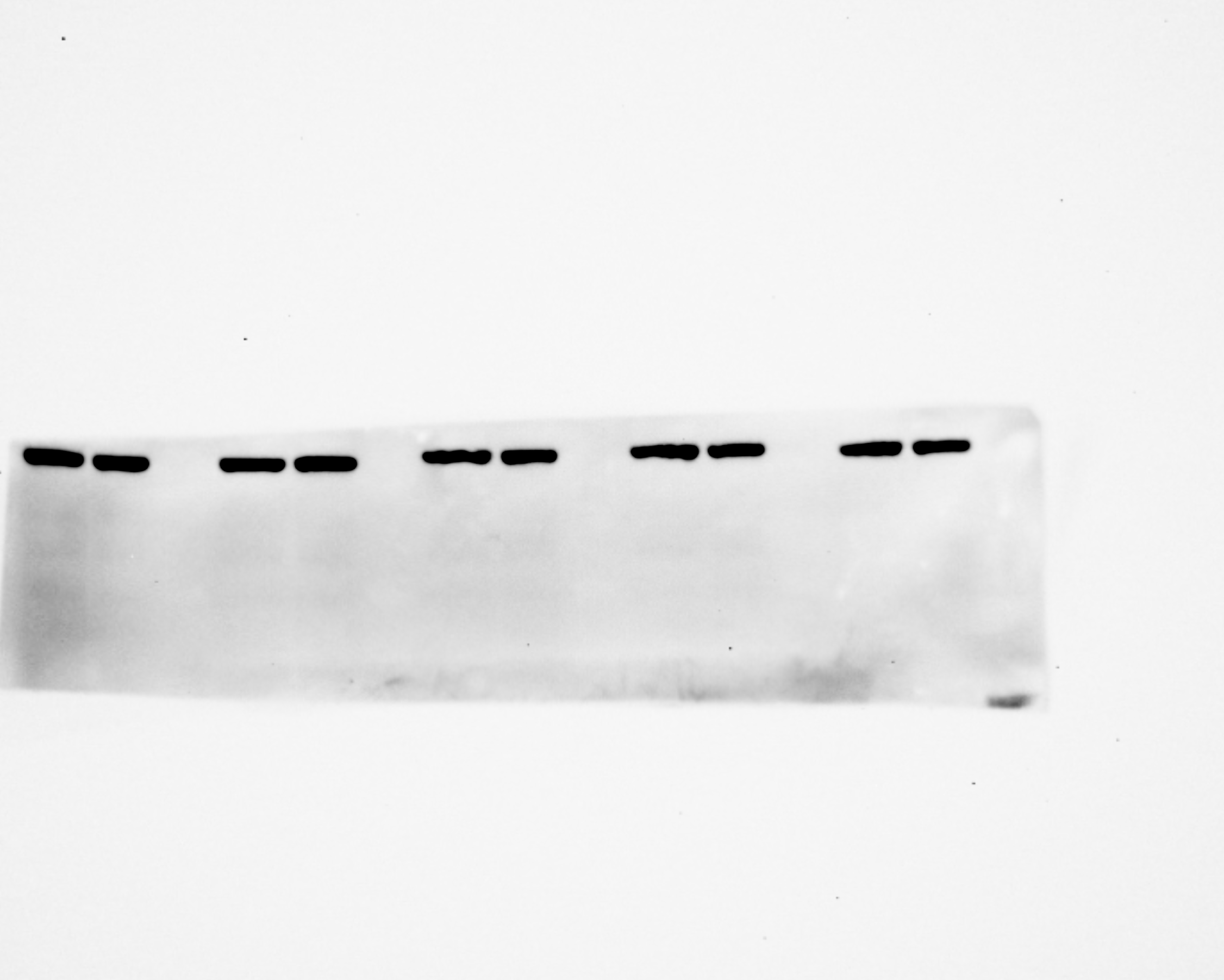


β-actin

Fig.5d FaDu IP


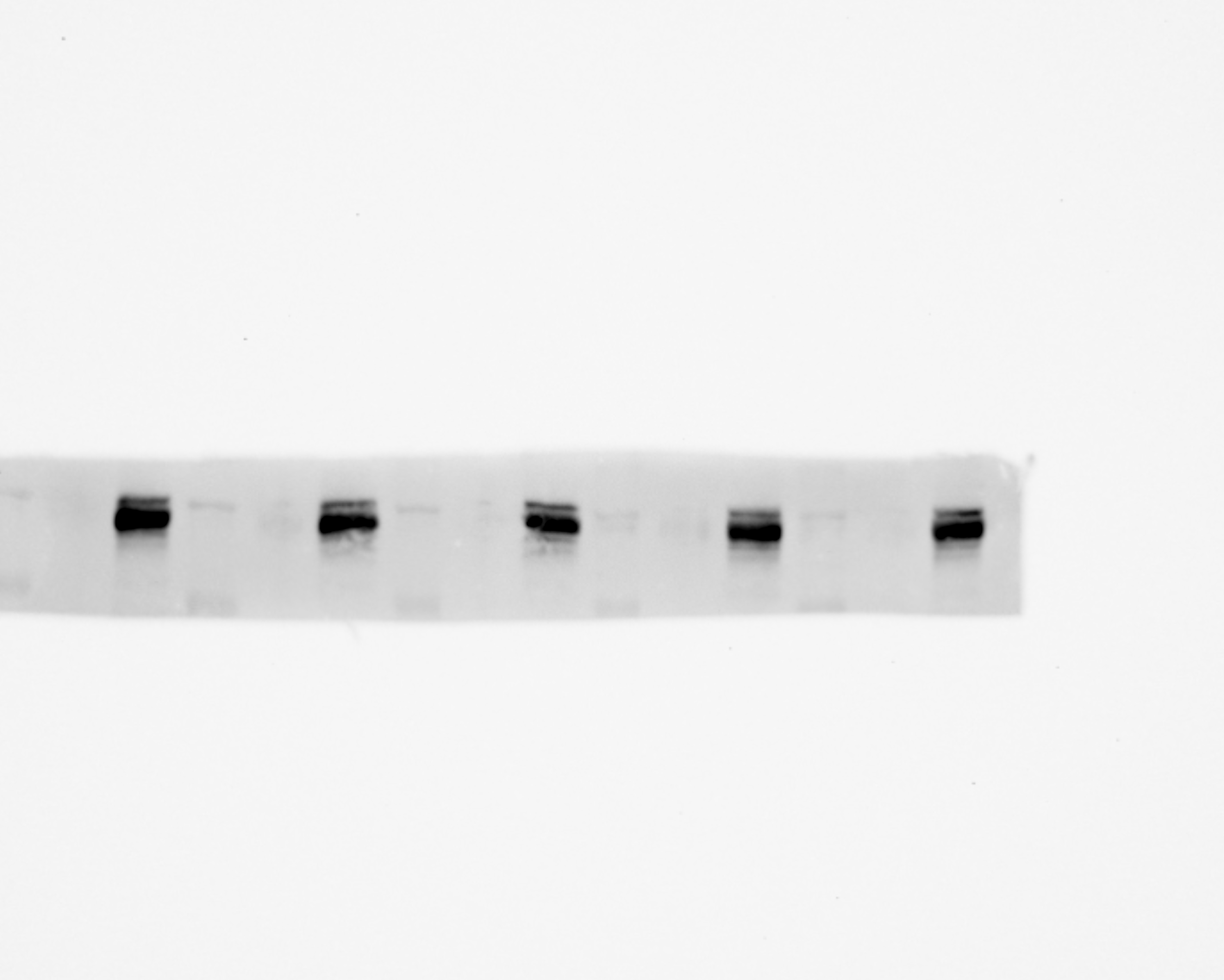

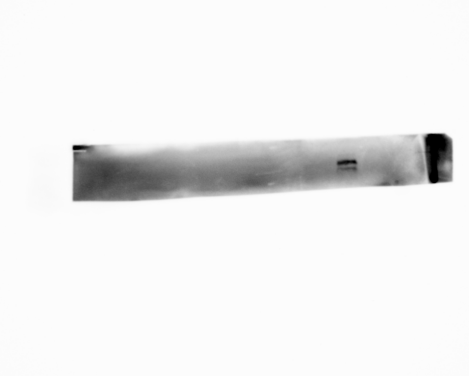


β-catenin

β-catenin


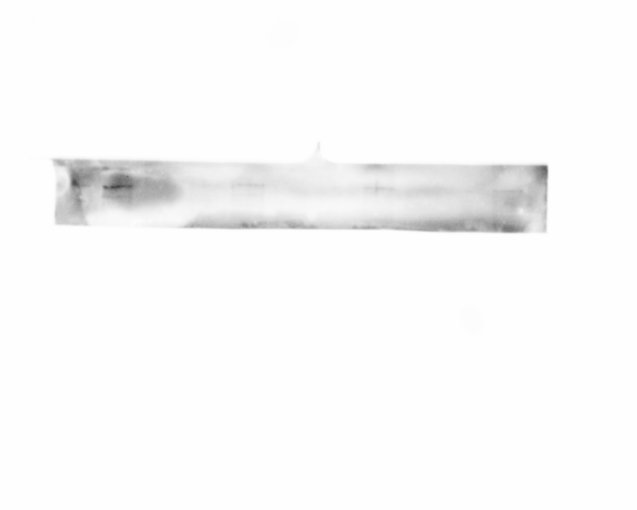

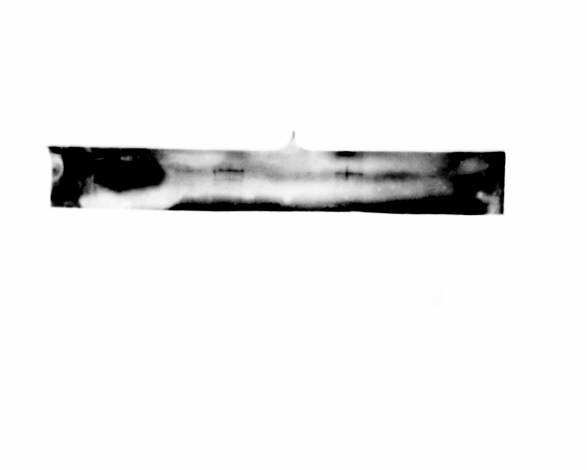


TCF4

TCF4


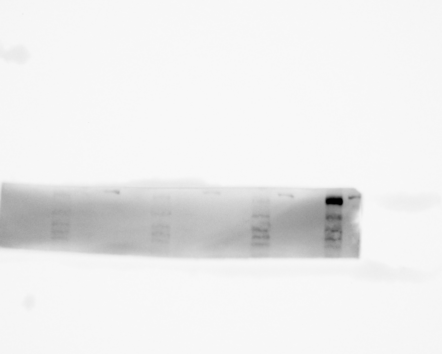

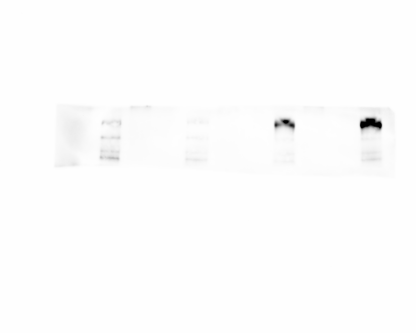

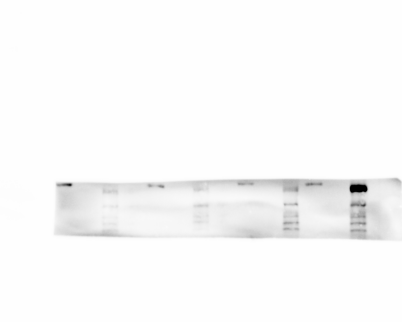


TRIB3

TRIB3

TRIB3

Fig.5e cal 27 Input


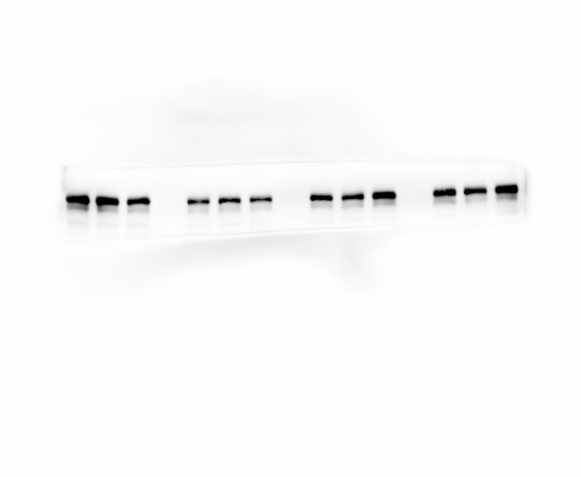

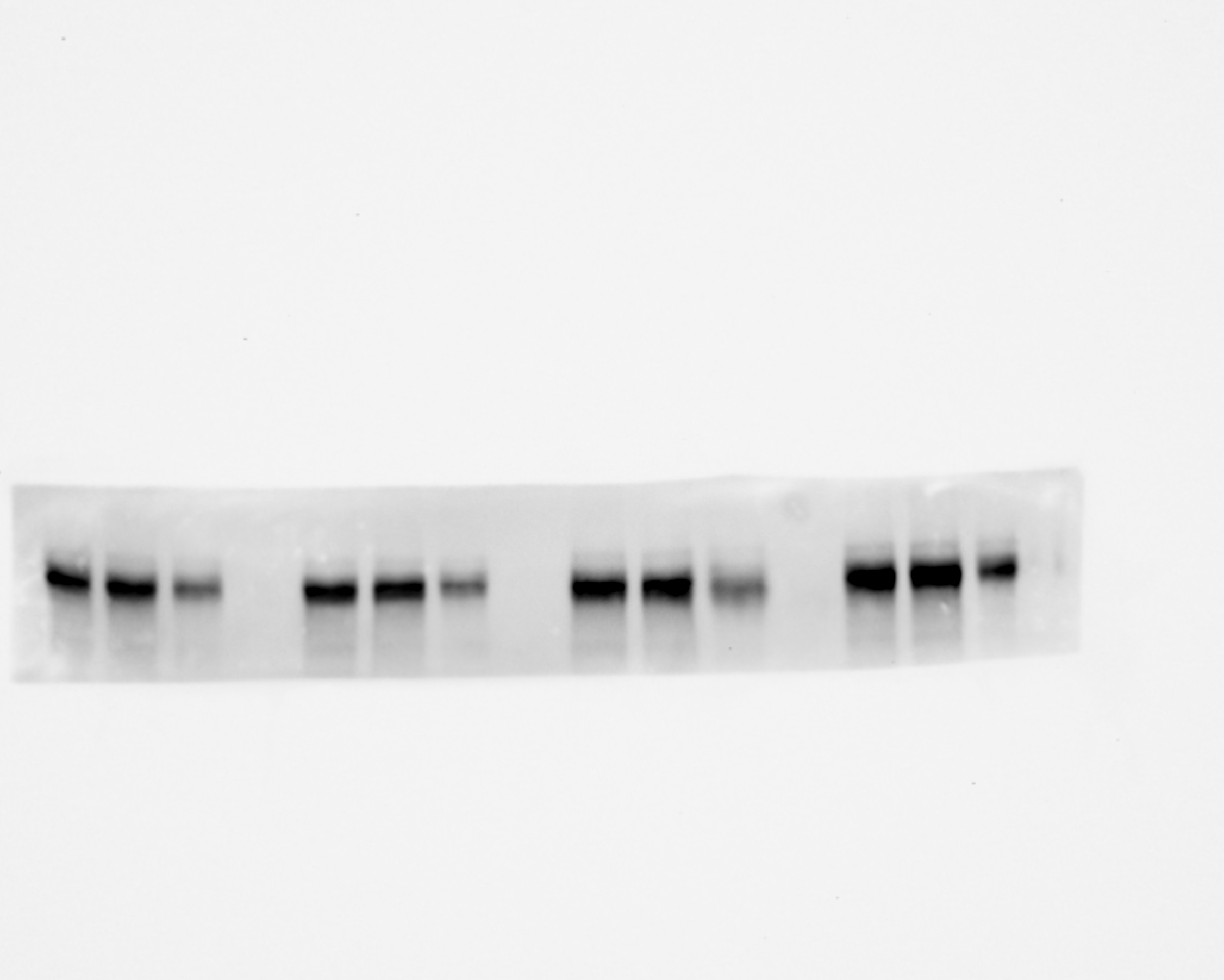


β-catenin

β-catenin


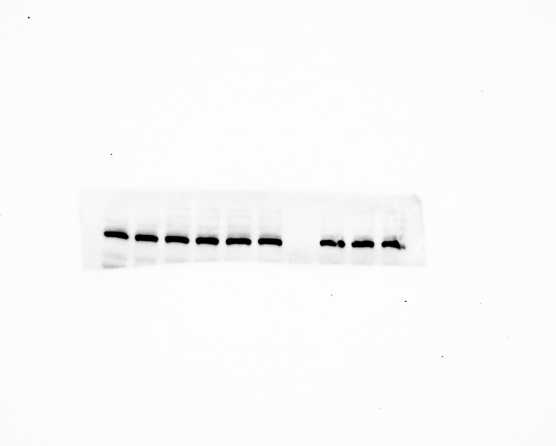


TCF4


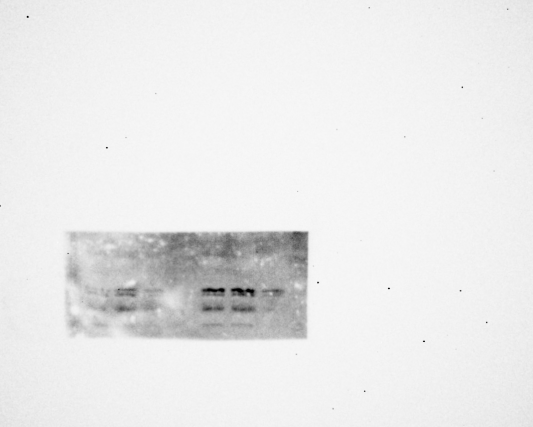

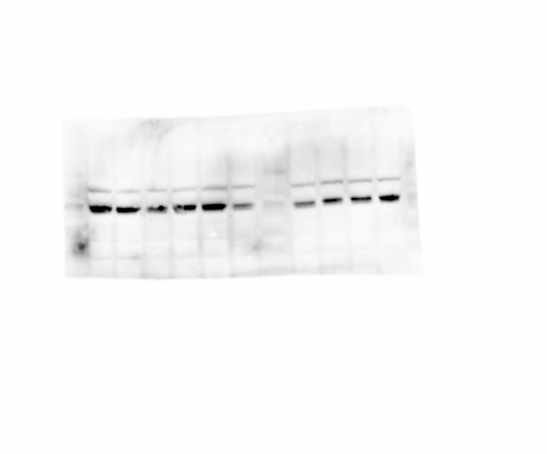


TRIB3

TRIB3


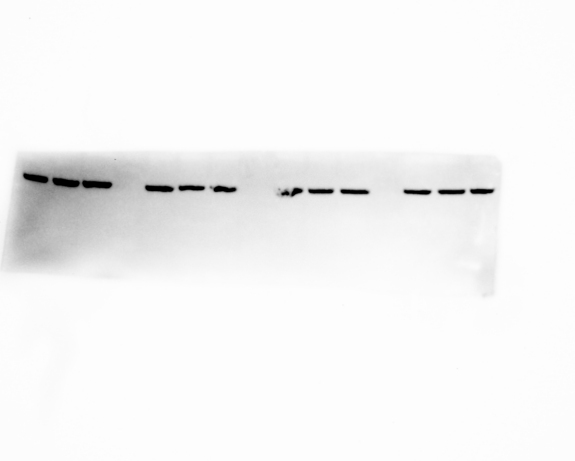


β-actin

Fig.5e cal 27 IP


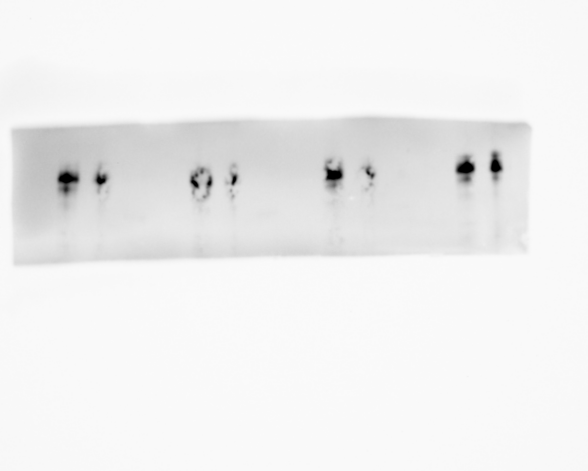

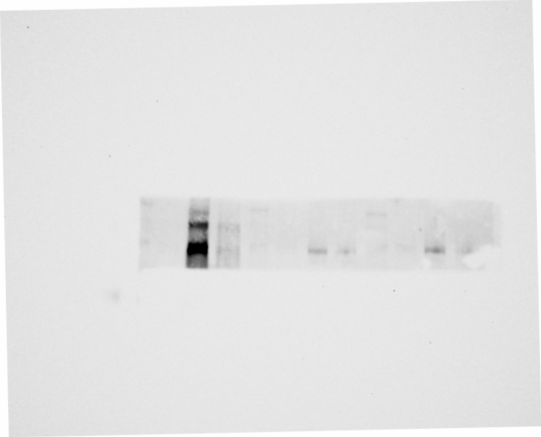

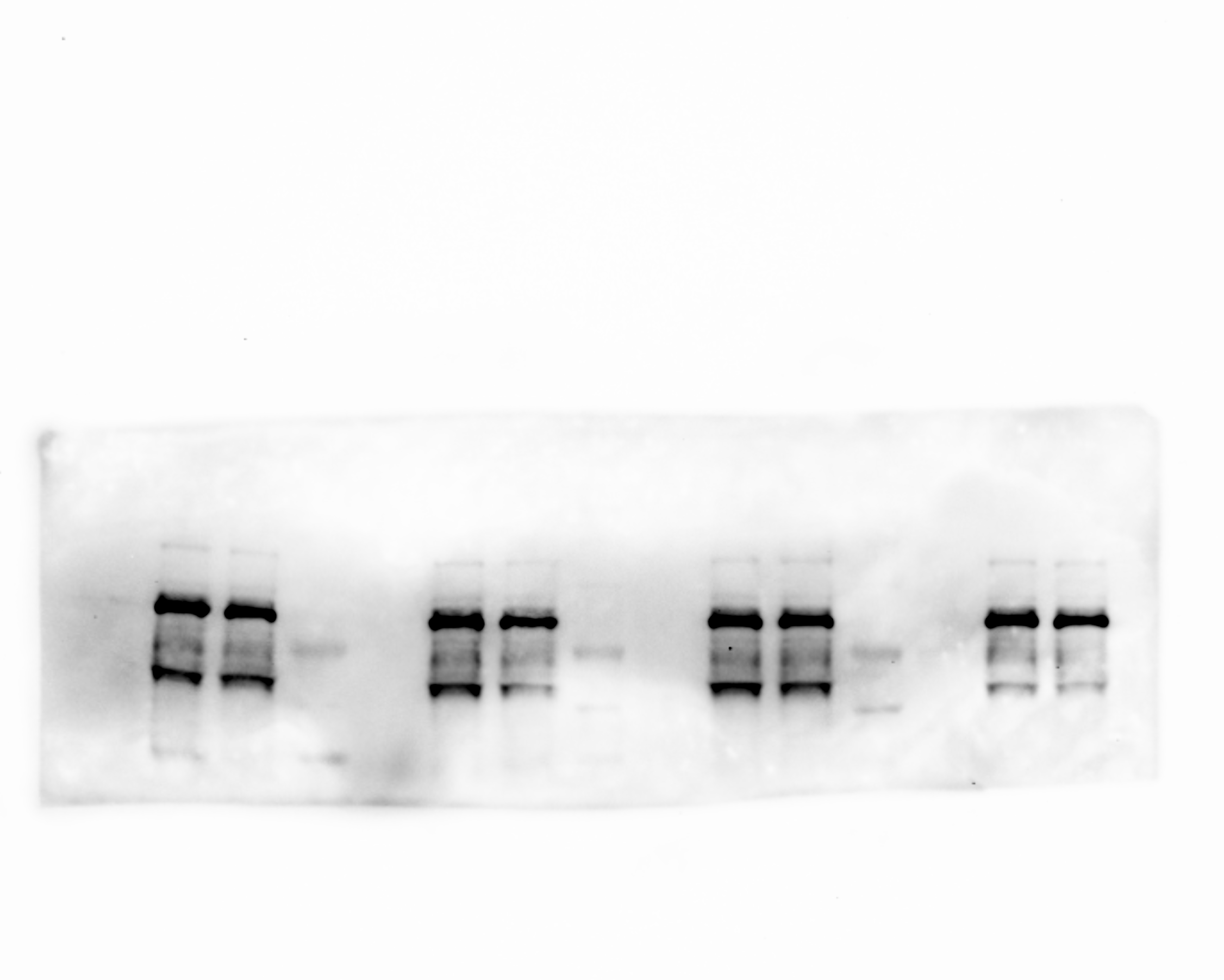


TCF4

β-catenin

β-catenin


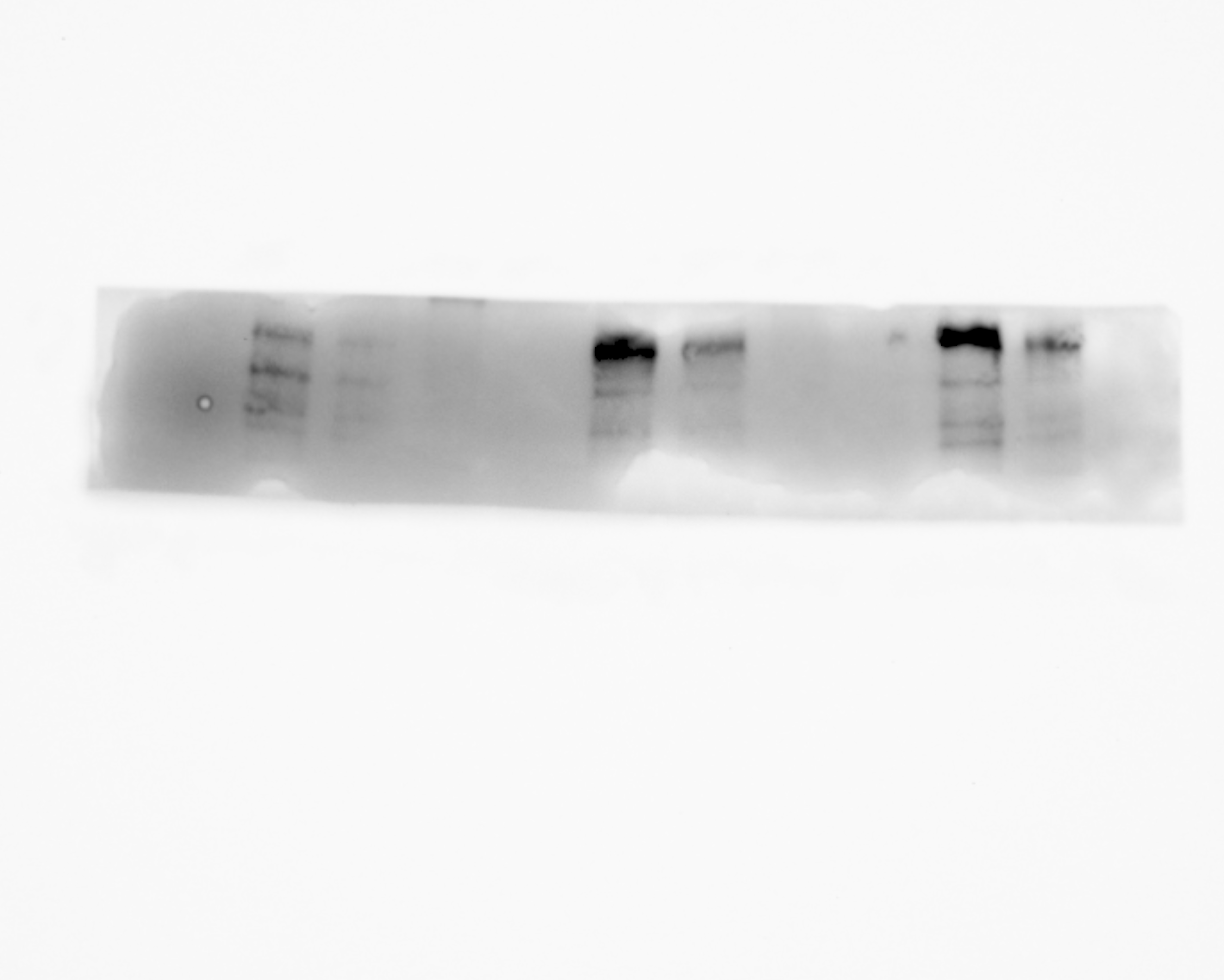

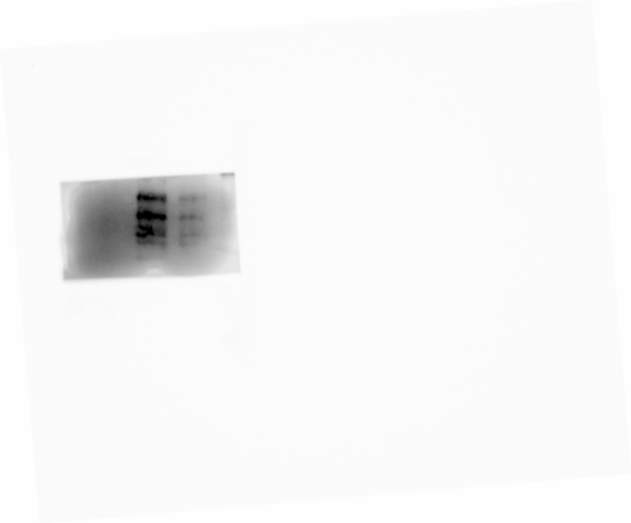


TRIB3

TRIB3

Fig.5f FaDu Input


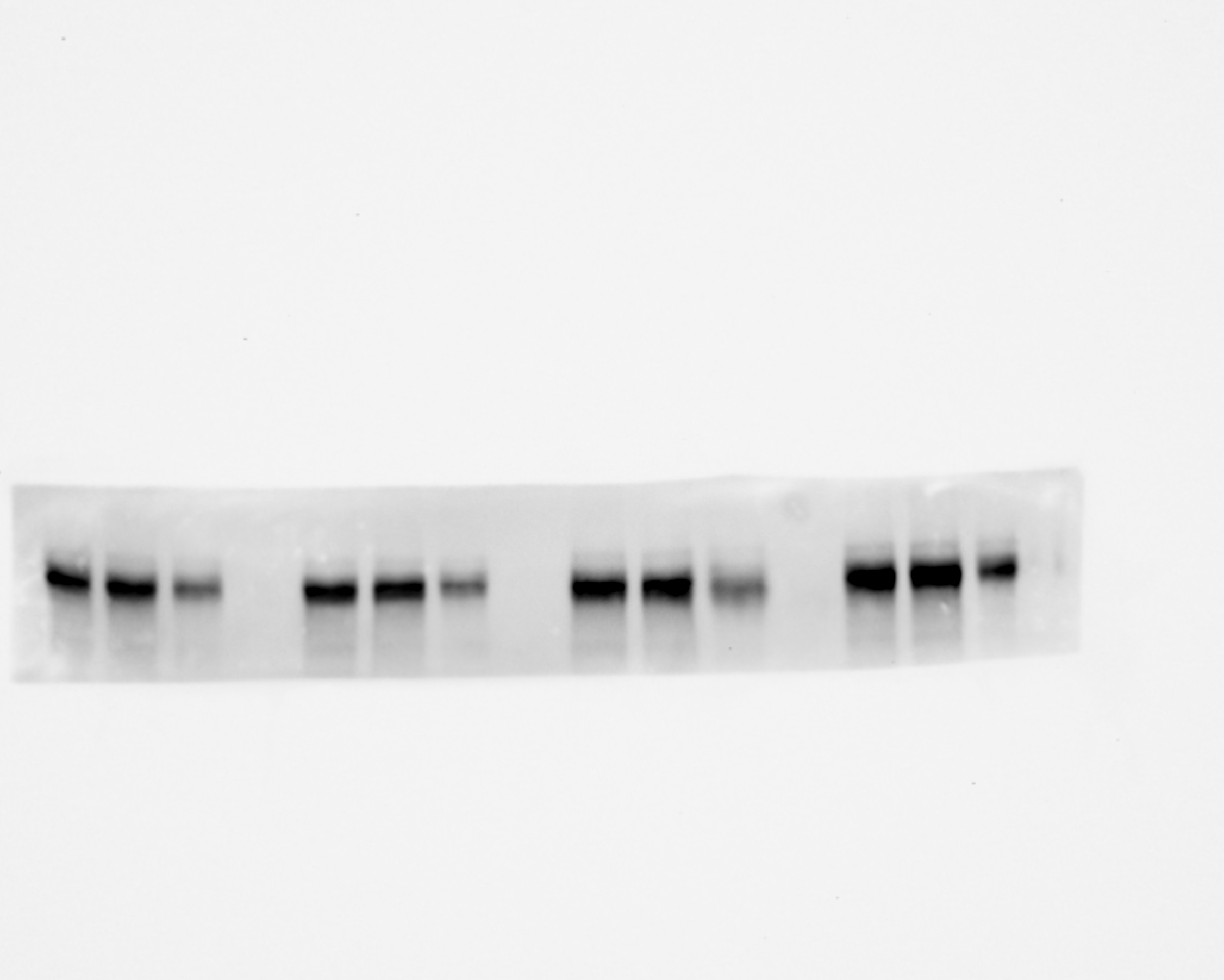

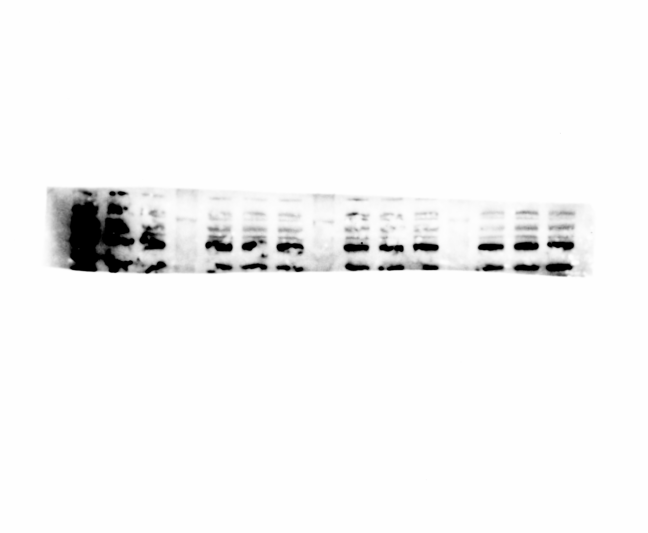


TCF4

β-catenin


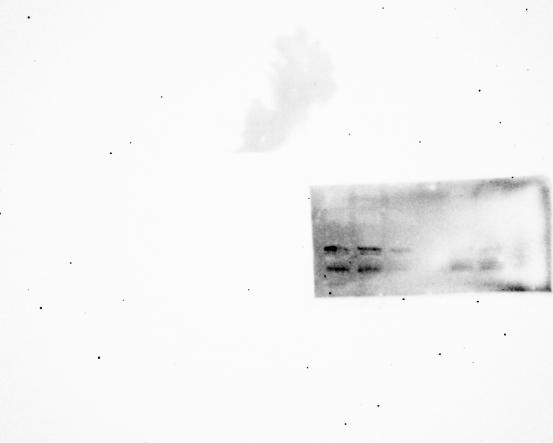

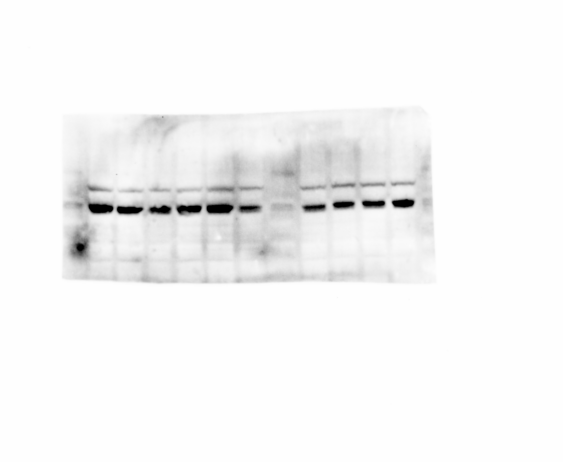


TRIB3

TRIB3


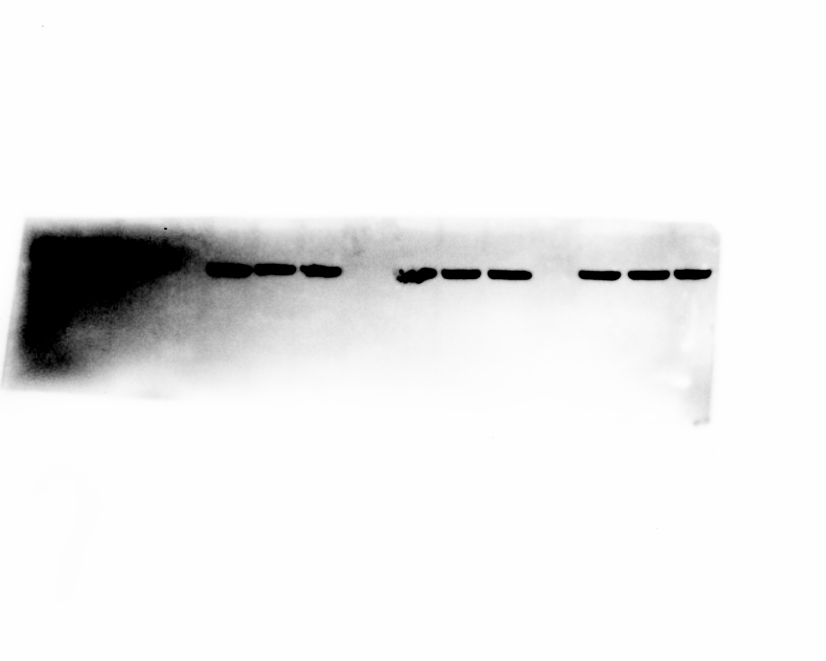


β-actin

Fig.5f FaDu IP


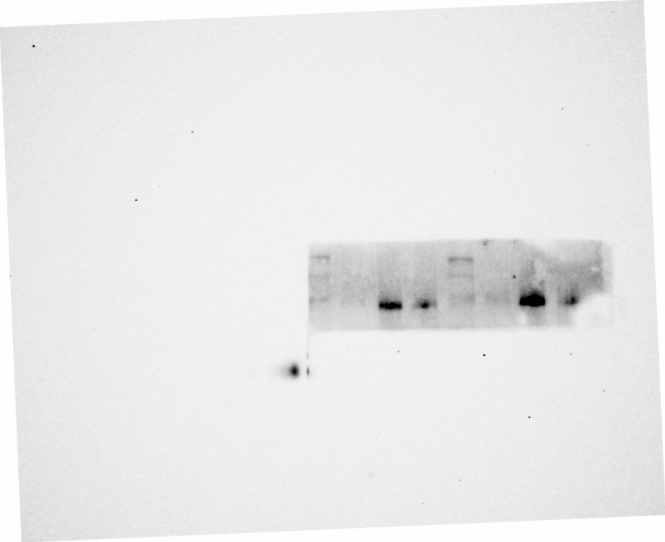


β-catenin


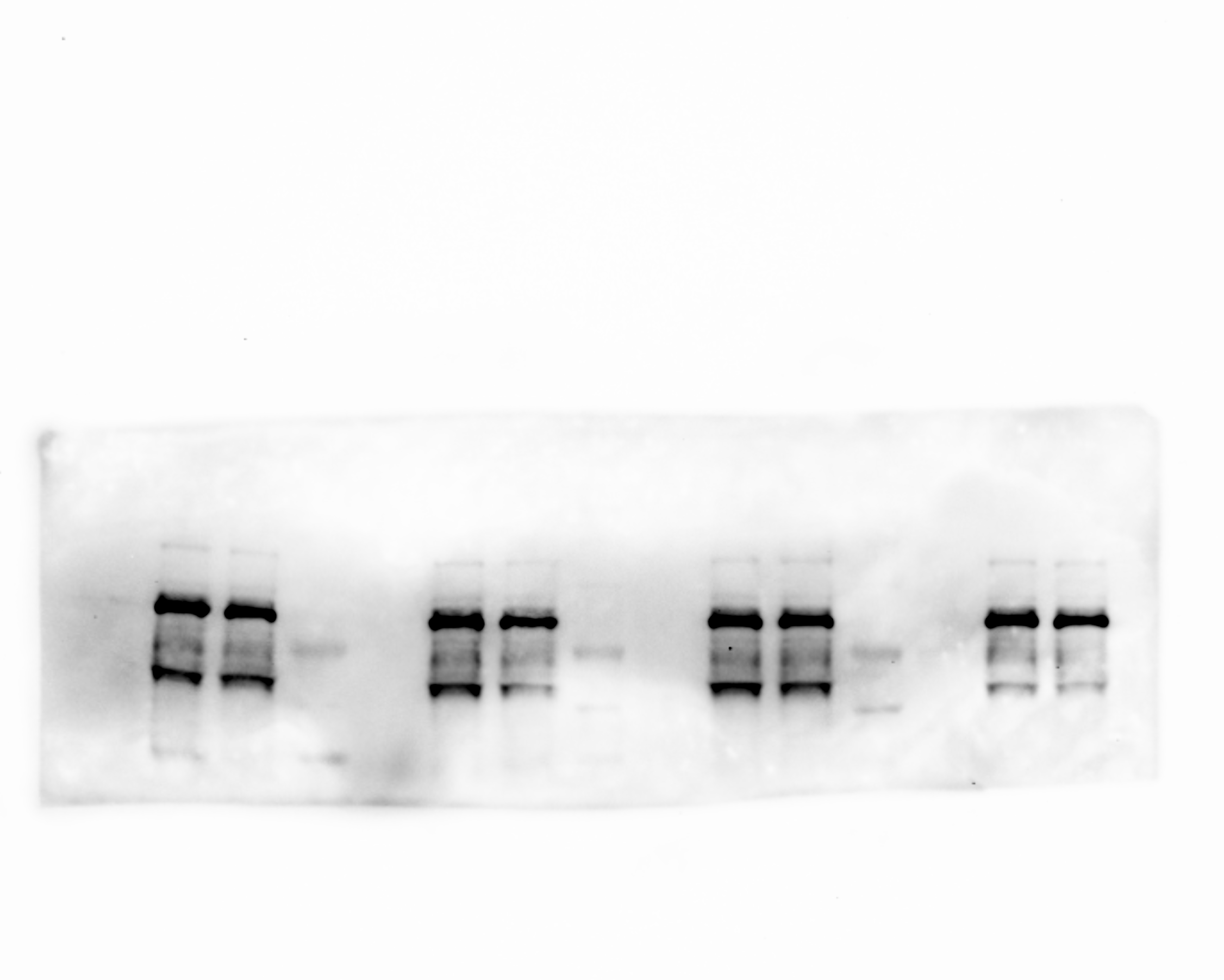


TCF4


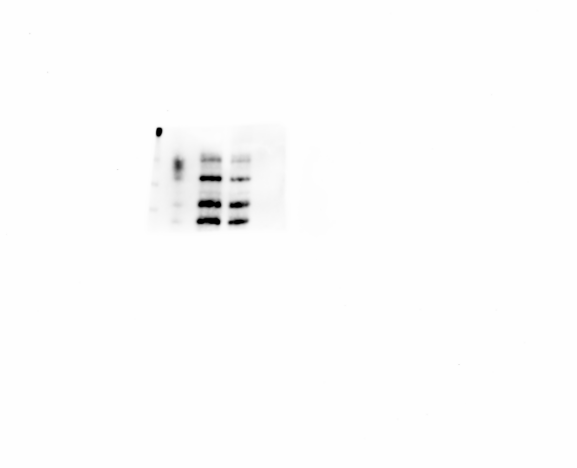

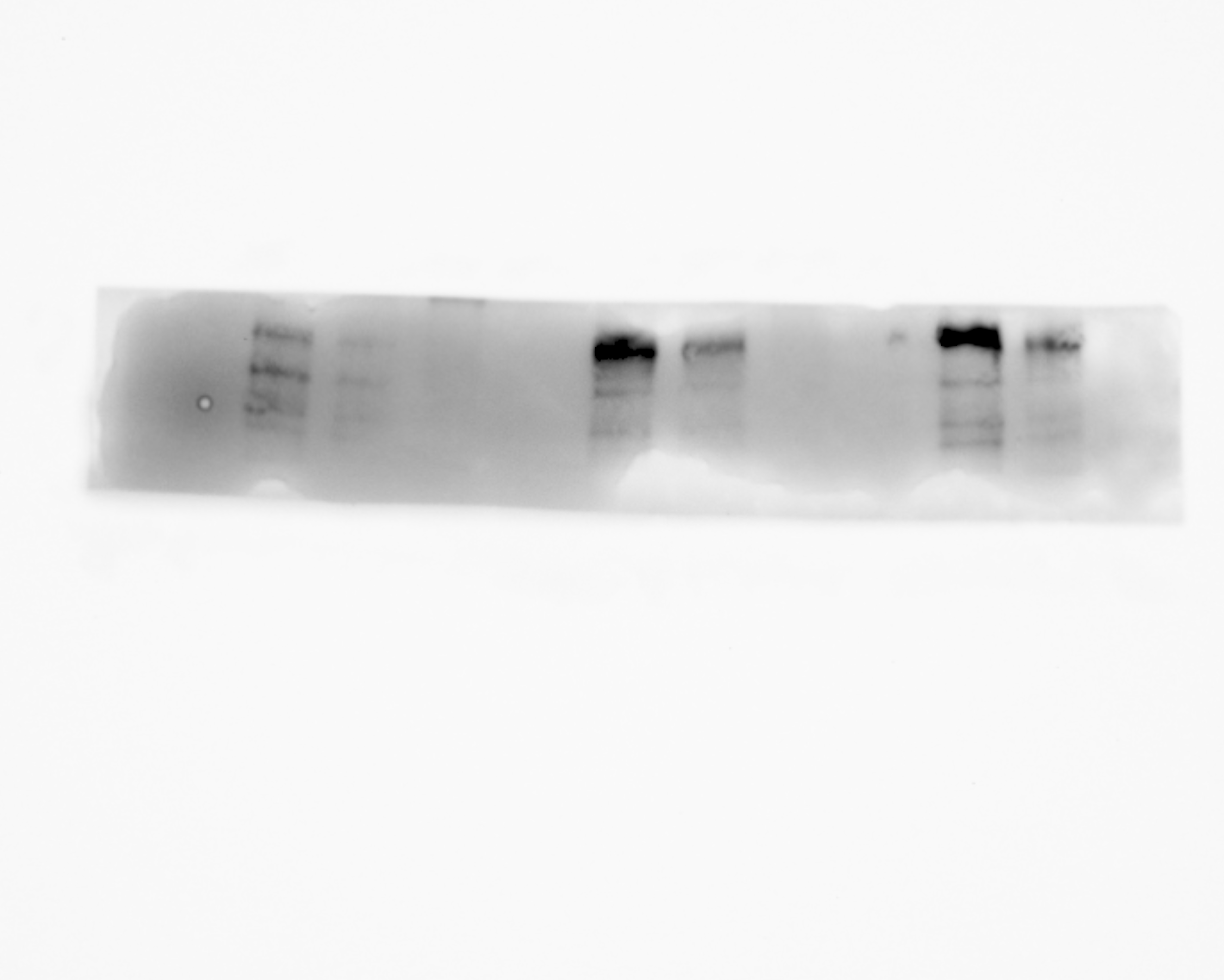


TRIB3

TRIB3

Fig.6i


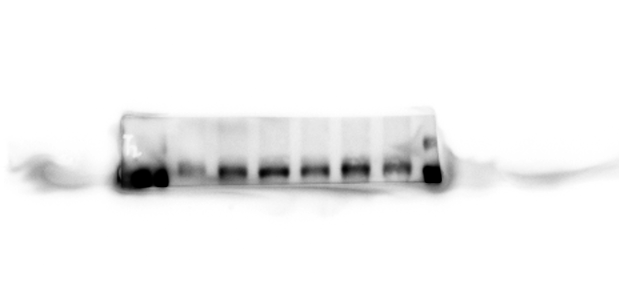


TCF4


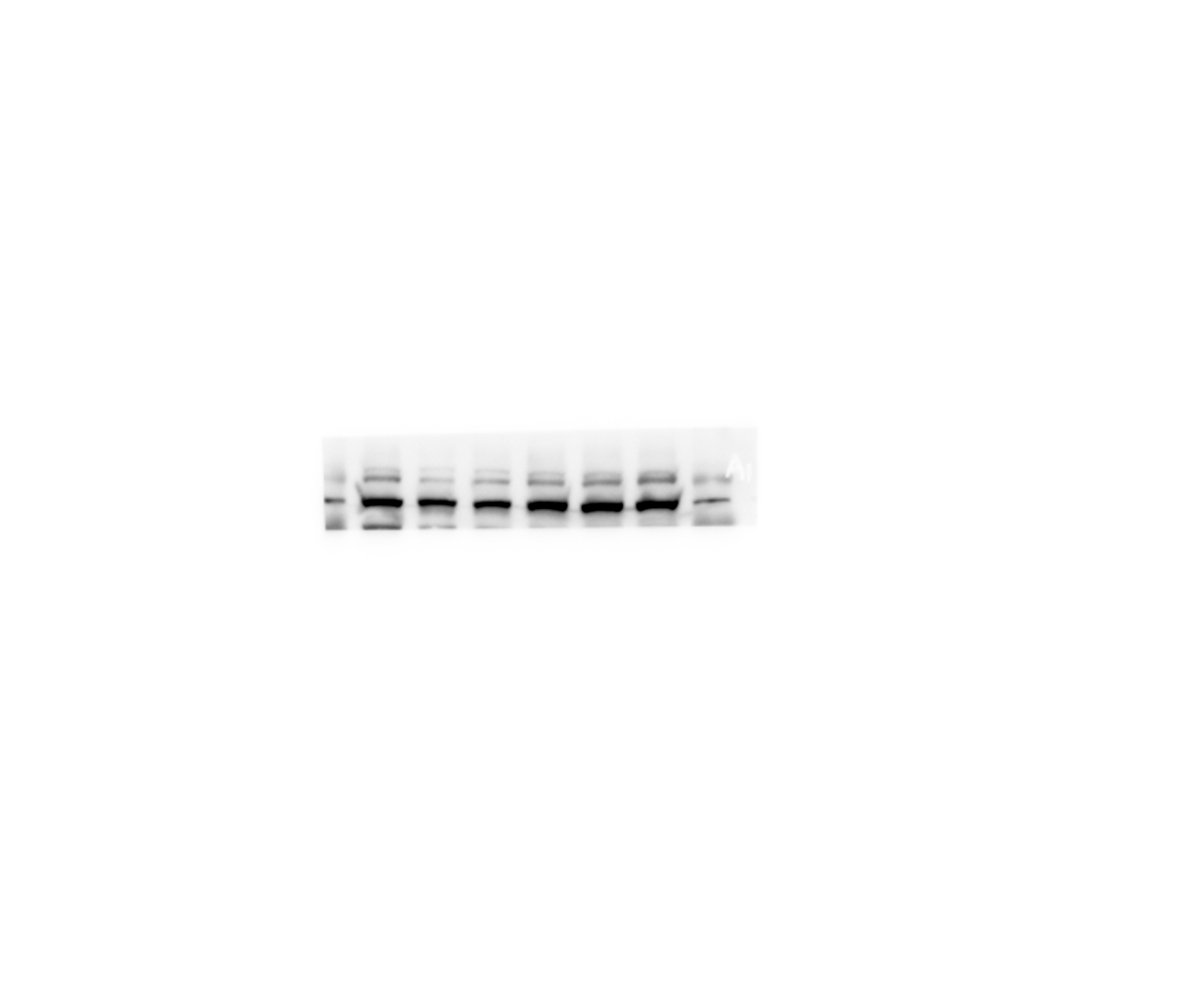

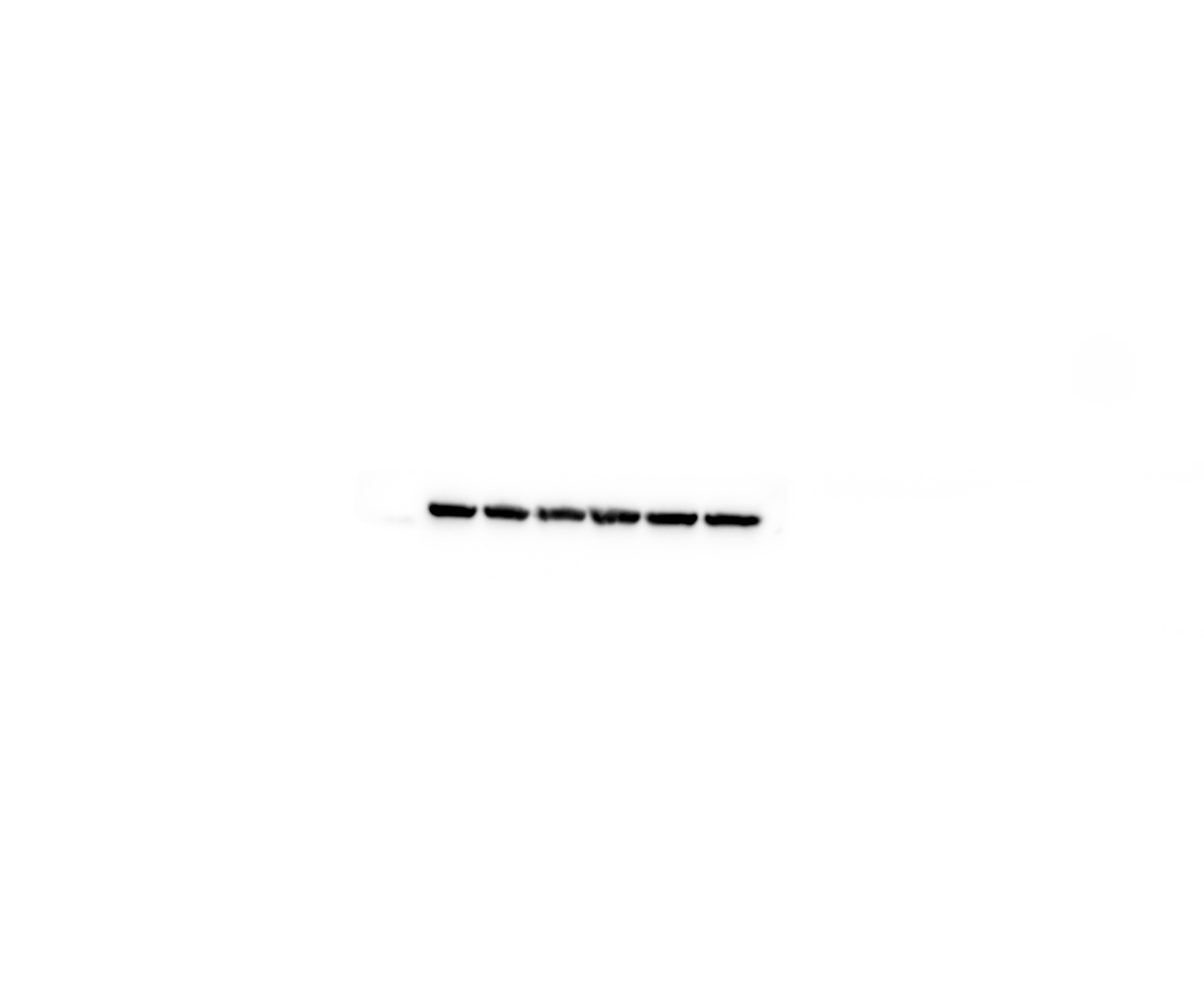


ALOXE3

β-actin

Fig.7a


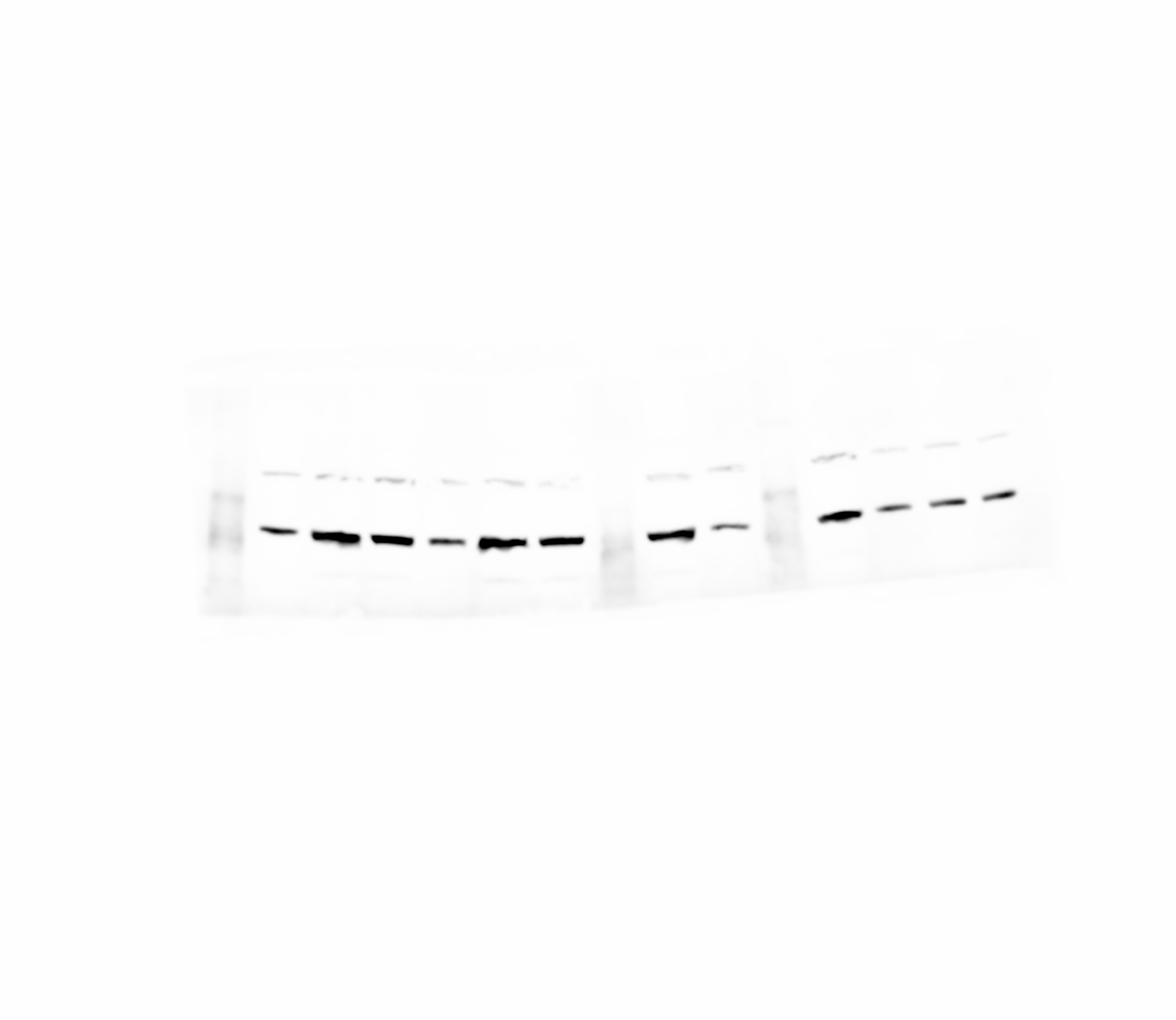

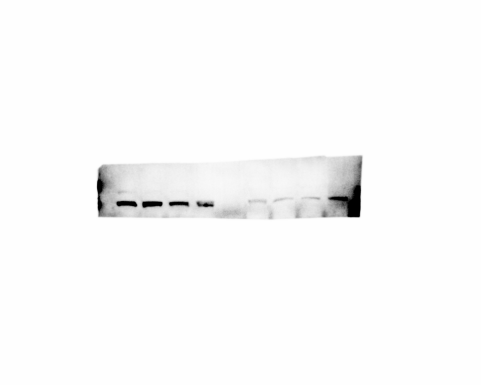


ALOXE3

ALOXE3


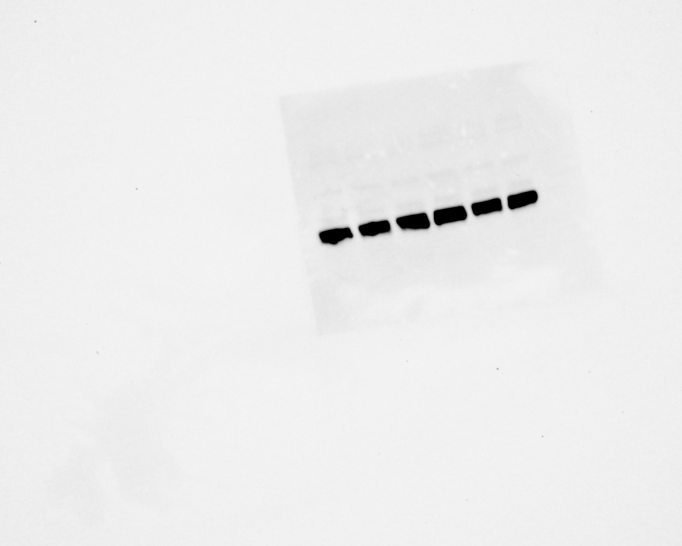

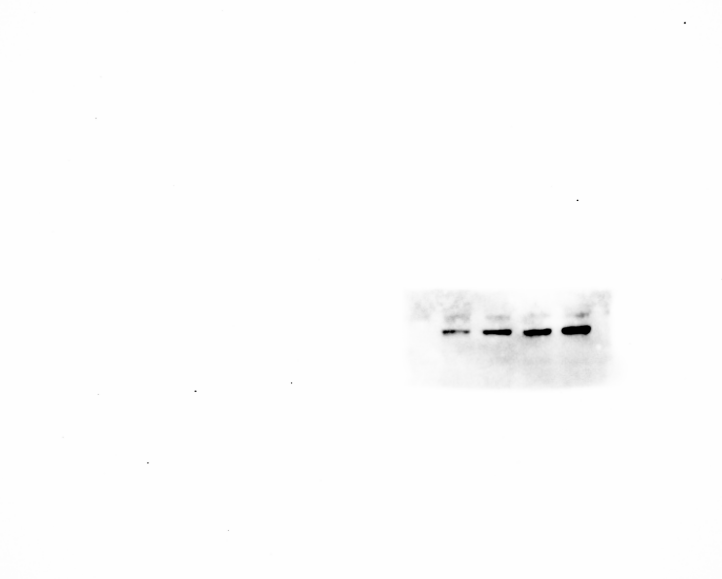


β-actin

β-actin

Fig.7b


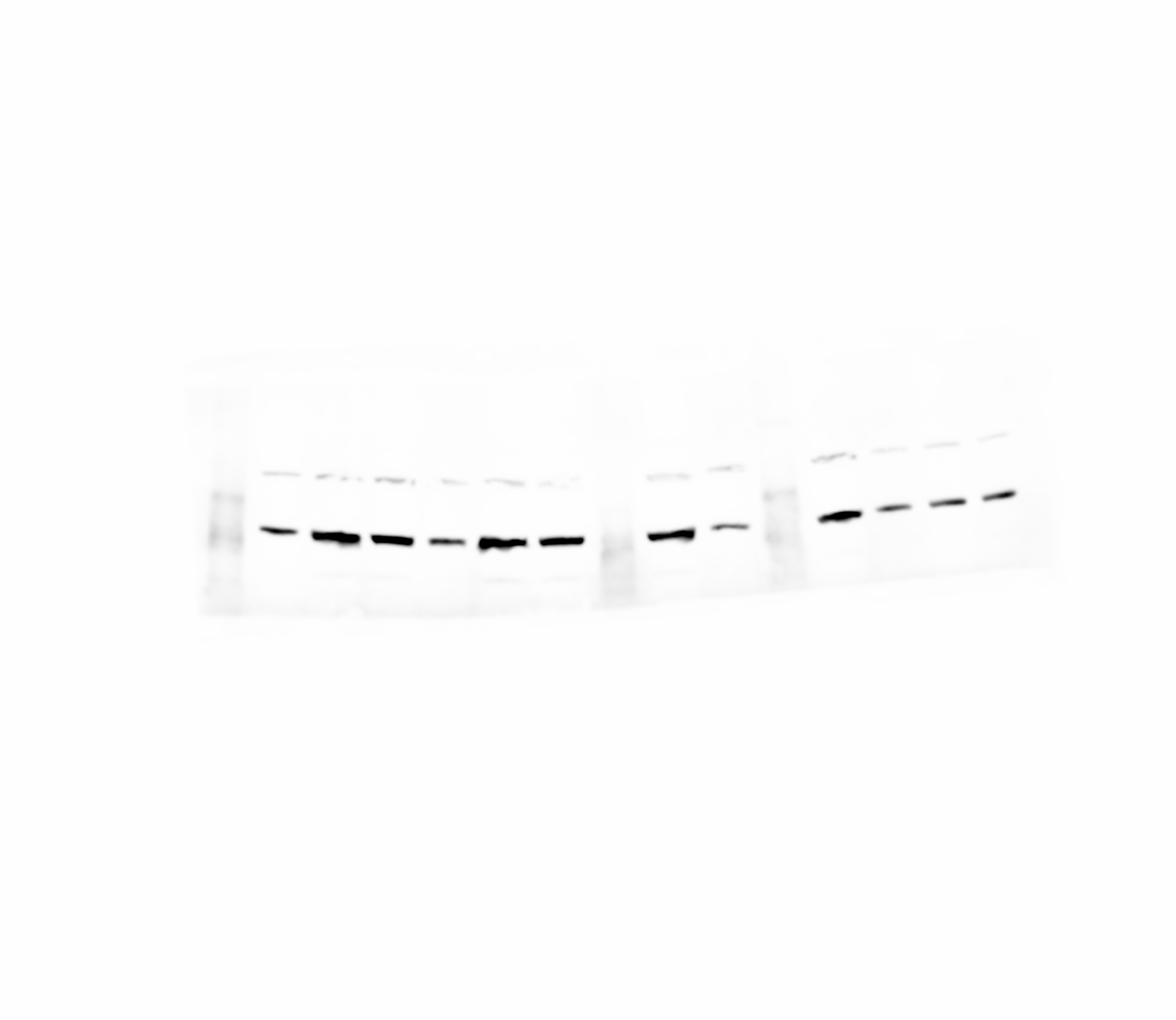

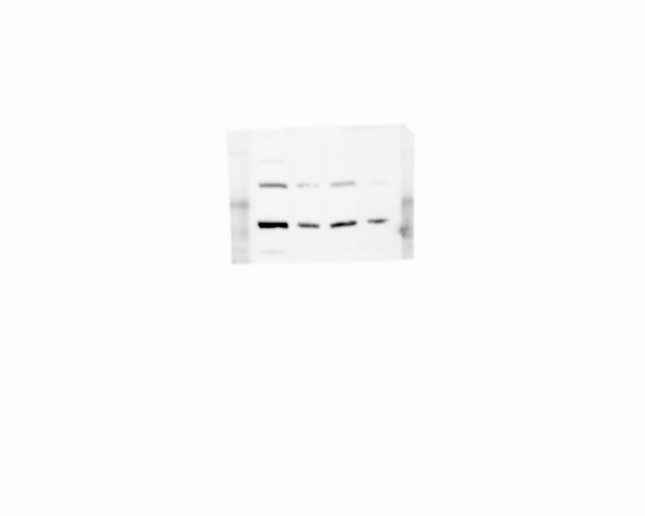


ALOXE3

ALOXE3


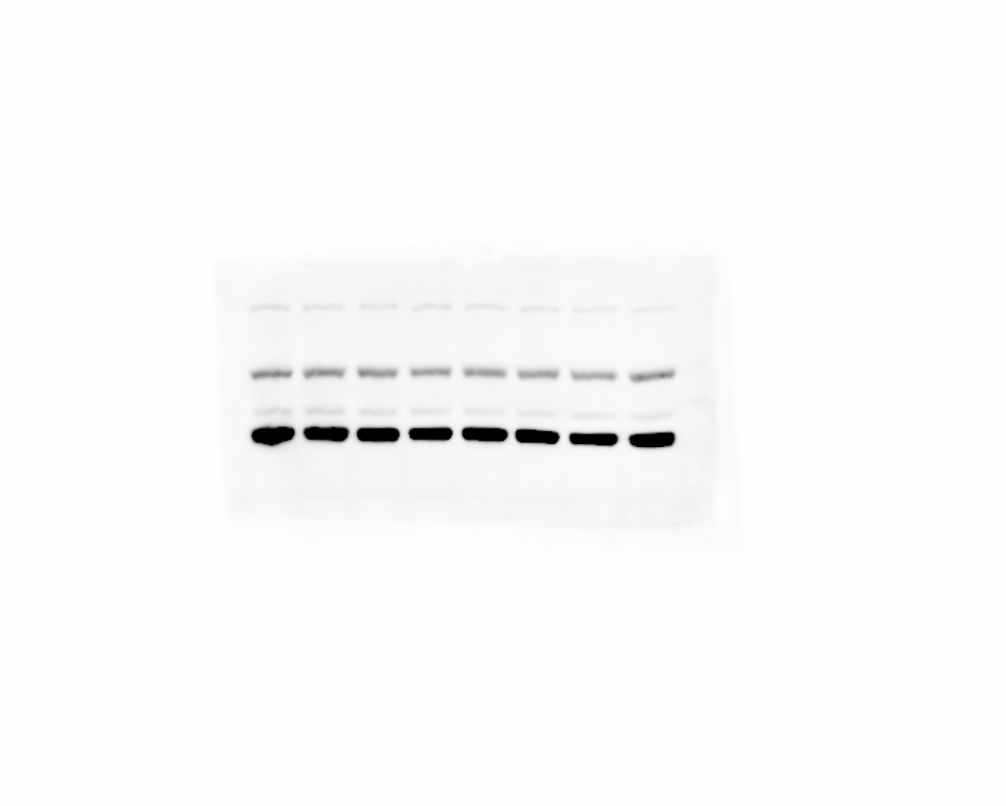


β-actin

Fig.8d


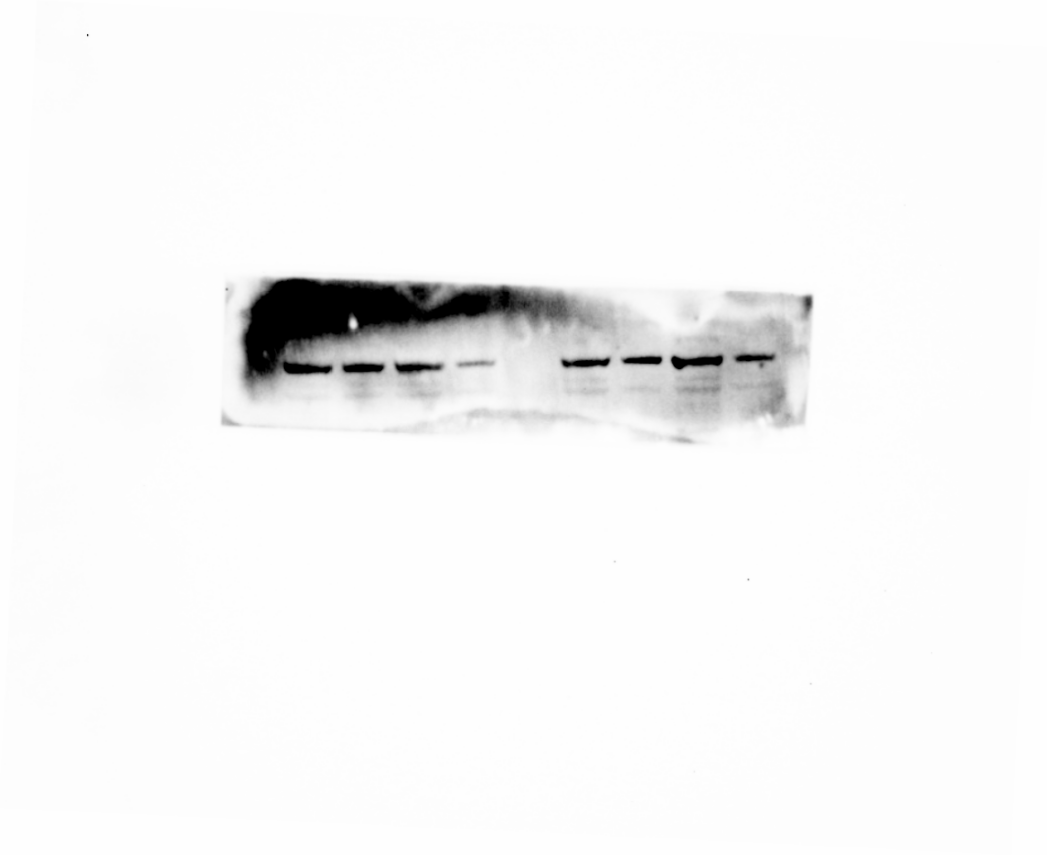

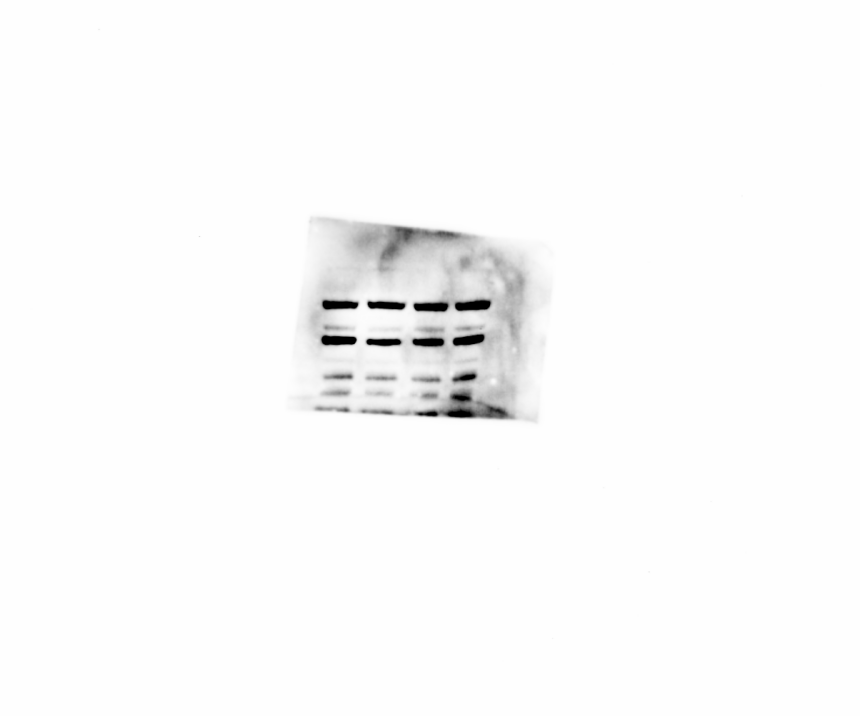


TRIB3

β-actin

Fig.S1a


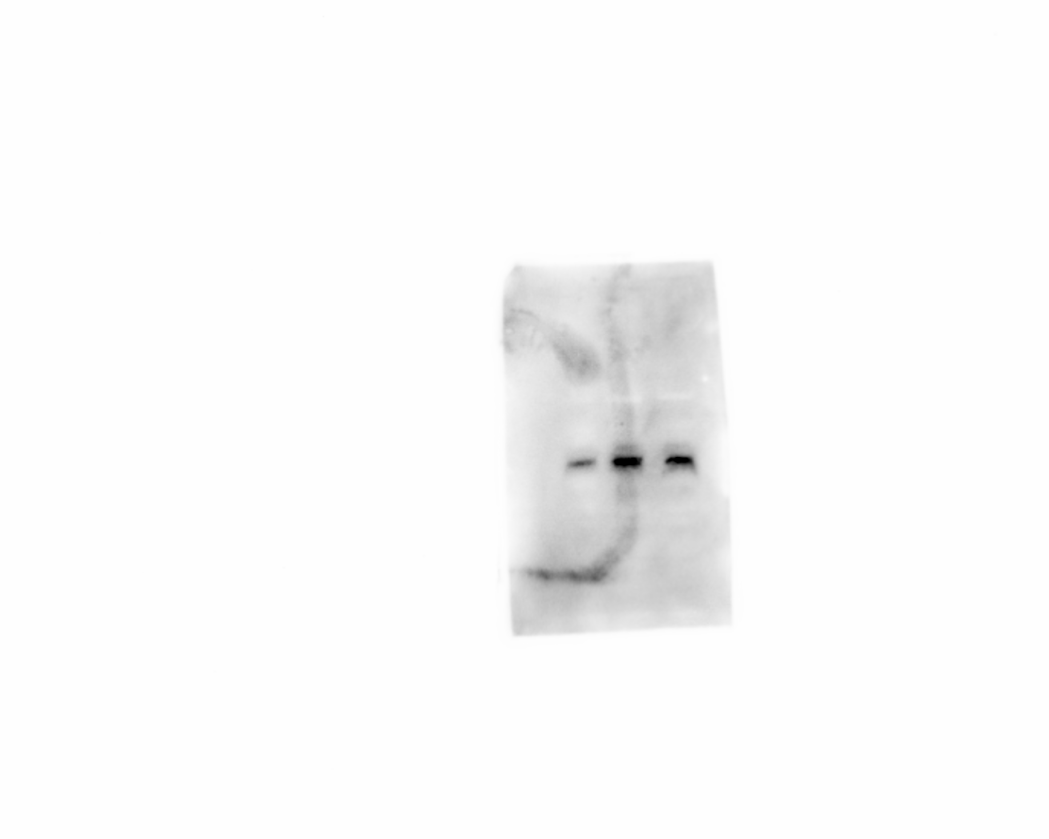

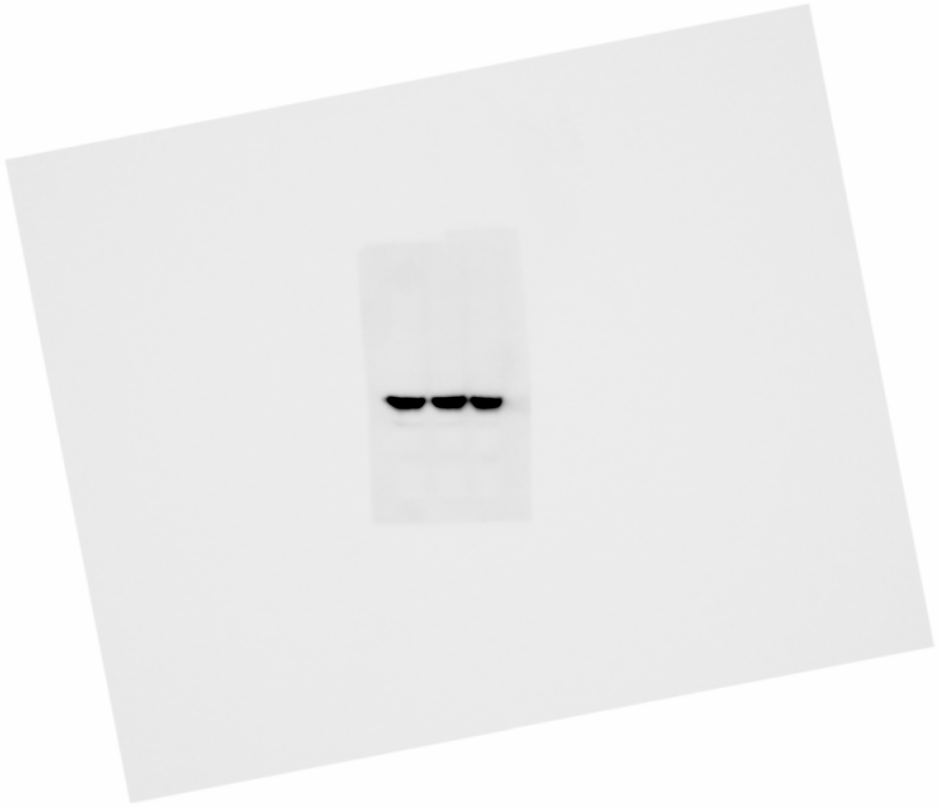


TRIB3

β-actin

Fig.S4b


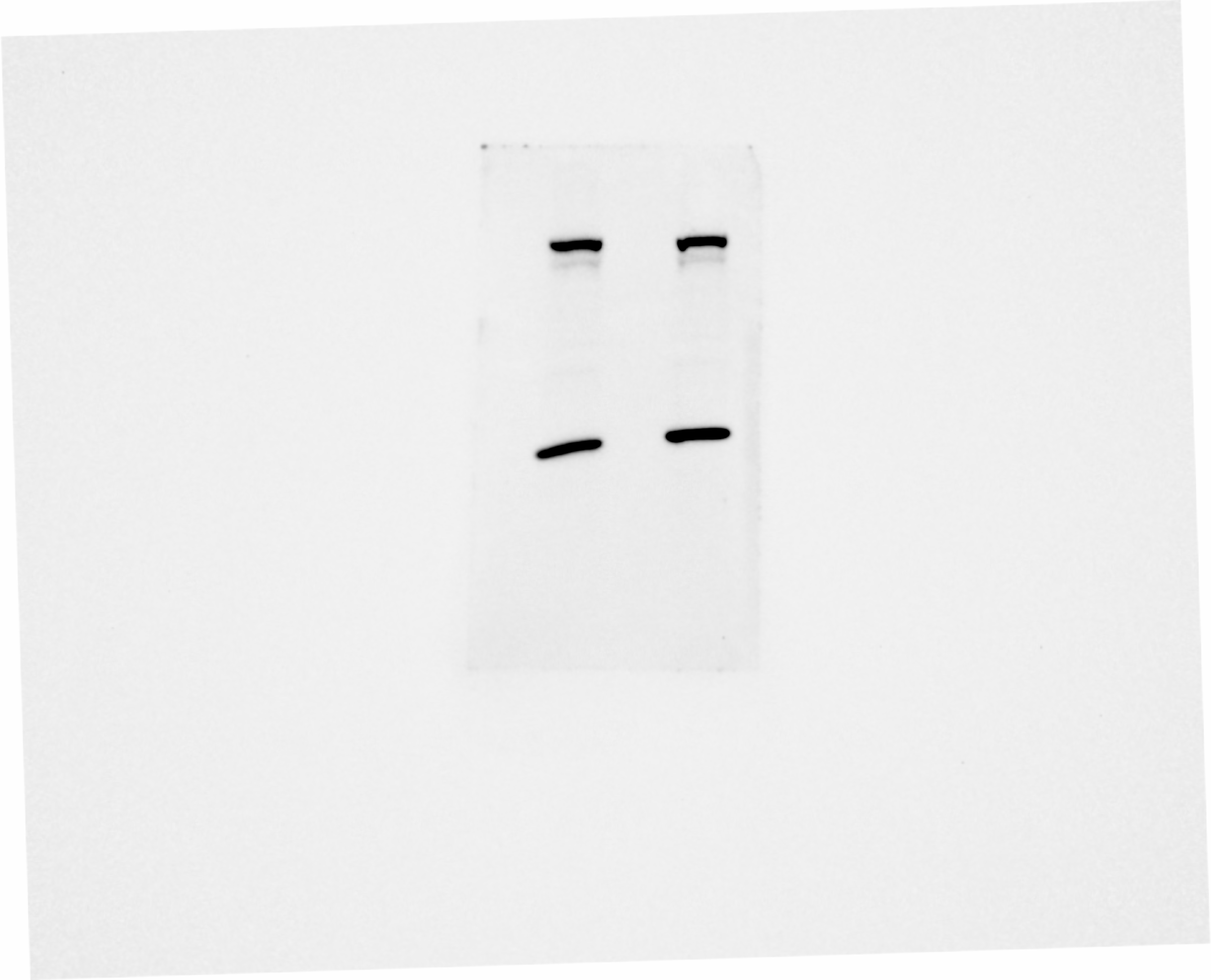

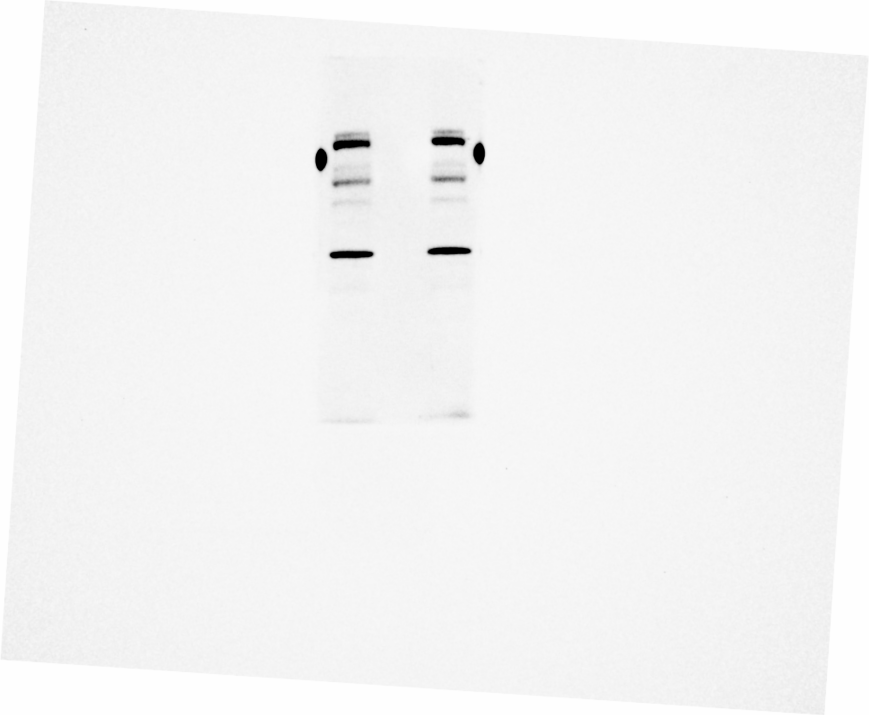


TCF4

β-catenin
